# Novel Roman domination-based graph energies for QSPR analysis of neuroprotective herbal compounds in Alzheimer’s disease treatment

**DOI:** 10.3389/fchem.2026.1731656

**Published:** 2026-03-13

**Authors:** A. Salini Jancy Rani, B. J. Balamurugan

**Affiliations:** Department of Mathematics, School of Advanced Sciences, Vellore Institute of Technology, Chennai, Tamil Nadu, India

**Keywords:** Alzheimer’s disease, graph energy, isomorphic molecular graph, physicochemical properties, QSPR analysis, Roman domination, Roman energy

## Abstract

Alzheimer’s disease (AD) is a progressive neurodegenerative disorder for which U.S. Food and Drug Administration (FDA)-approved drugs provide only temporary symptomatic relief and often cause adverse effects. Plant-derived bioactive phytochemicals are emerging as promising alternatives due to their multi-targeted neuroprotective properties and reduced toxicity. In this article, herbal anti-Alzheimer’s compounds are analyzed using a novel graph molecular modeling. In chemical graph theory, molecular structures are represented as isomorphic molecular graphs $G\left(V,E\right)$ , where V and E denote the set of vertices (atoms) and edges (chemical bonds) respectively. Classical graph matrices such as adjacency and Laplacian matrices capture the molecular connectivity but fail to account for hierarchical differences in atomic influence. To address this limitation, Roman domination is employed to represent the hierarchical dominance of atoms within molecular structures. A Roman domination function (RDF) on a graph $G\left(V,E\right)$ is a mapping $f:V\to \left\{0,1,2\right\}$ such that every atom v with $f\left(v\right)=0$ has at least one adjacent atom u with $f\left(u\right)=2$ , reflecting the hierarchical dominance within the isomorphic molecular graph. Based on this principle, the Roman domination-based matrices and corresponding graph energies are introduced in this article. Quantitative Structure-Property Relationship (QSPR) graph models are developed using the Roman energies through linear, quadratic and cubic regression analysis. The results demonstrate superior performance compared to classical approaches, with the quadratic regression showing the strongest correlations and lowest standard error. Internal validation through the Y-randomization and Leave-One-Out Cross-Validation methods confirmed the stability of the models, while external validation on the herbal compound Kaempferol ( r=0.993 ) further supported their predictive reliability. These findings underscore the robustness of Roman energies, establishing them as powerful molecular descriptors that offer enhanced accuracy in the QSPR analysis and hold promise for applications in drug design, materials informatics and computational chemistry.

## 1 Introduction

Alzheimer’s disease (AD) is a progressive neurodegenerative disorder. It is the most common form of dementia in aged people and is characterized by the accumulation of abnormal protein deposits in the brain, particularly beta-amyloid plaques and misfolded tau (Bruno et al., 2023). These pathological changes lead to the degeneration of brain cells, disrupting communication between neurons and causing cognitive decline. This severe brain ailment disrupts memory, thinking and daily functioning, resembling a puzzle where pieces of one’s memory and abilities slowly go missing over time. Although there is no cure for Alzheimer’s, scientists have developed some U.S. Food and Drug Administration (FDA)-approved drugs to assist in managing its symptoms. These medications (Alhazmi and Albratty, 2022; Grossberg et al., 2019), including atypical antipsychotic (Brexpiprazole), acetylcholinesterase inhibitors (Donepezil, Galantamine and Rivastigmine) and Glutamate inhibitor (Memantine) are designed to provide temporary relief from the cognitive issues associated with Alzheimer’s, such as memory loss and confusion.

Motivated by the limitations of these synthetic drugs, which provide only temporary symptomatic relief and are often associated with adverse effects (D'Souza et al., 2024), plant-derived bioactive phytochemicals are considered as promising alternatives due to their multi-targeted neuroprotective properties and reduced toxicity. Such natural compounds are believed to modulate key pathological pathways in AD with comparatively fewer side effects, making them valuable candidates for safer, long-term interventions (Yuan et al., 2019). In this context, a total of 21 plant-derived bioactive phytochemicals (Alhazmi and Albratty, 2022; Alsenani, 2024; Soni et al., 2024) has been examined for their therapeutic potential against Alzheimer’s disease. These include Huperzine A, Curcumin, Ginkgolide, Bilobalide, Ginsenoside Rg1, Resveratrol, Epigallocatechin-3-gallate, Quercetin, Baicalein, Asiaticoside, Catalpol, Ferulic acid, Salvianolic acid, Schisandrin, Rhynchophylline, Glycyrrhizin, γ-Linolenic acid, Crocin, Isorhamnetin, Allicin and Gingerol. These compounds, extracted from diverse herbal sources, exhibits one or more neuroprotective activities such as acetylcholinesterase inhibition, antioxidant, anti-inflammatory action, mitochondrial protection and amyloid-β modulation. A detailed summary of each phytochemical compound’s chemical formula, source herb, and anti-Alzheimer’s biological activity is provided in Table 1. These phytoconstituents form the basis for further Quantitative Structure-Property Relationship (QSPR) analysis using graph-theoretic descriptors, aimed at modeling and predicting their anti-Alzheimer’s potential with high reliability. The QSPR analysis based on various graph descriptors has been extensively discussed in the literature (Kalaimathi and Balamurugan, 2023; Kara et al., 2025a; Kara et al., 2025b; Kirana et al., 2024; Parveen et al., 2024; Rani and Balamurugan, 2025; Rauf et al., 2024; Raza and Munir, 2024; Tamilarasi and Balamurugan, 2025; Wazzan and Ozalan, 2023; Yogalakshmi and Balamurugan, 2025). Several studies have specifically addressed Alzheimer’s disease-related QSPR modeling. In this context, Ahmed et al. has developed QSPR regression models using degree-based (Ahmed et al., 2024) and eccentricity-based (Ahmed et al., 2025a) topological descriptors to find the physicochemical properties of anti-Alzheimer’s drugs. Moreover, they applied Artificial Neural Network and Random Forest approaches to further enhance predictive performance (Ahmed et al., 2025b).

**TABLE 1:** Source herb and biological activity of anti-Alzheimer’s herbal compounds.

S. no	Active compound	Source herb(s)	Biological activity
1	Huperzine A $C_{15}H_{18}N_{2}O$	*Huperzia serrata*	Natural inhibitor of acetylcholinesterase
2	Curcumin $C_{21}H_{20}O_{6}$	*Curcuma longa*	Anti-inflammatory, antioxidant, antitumor, antibacterial activities
3	Ginkgolide $C_{20}H_{24}O_{9}$	*Ginkgo biloba*	Neuroprotection, reduces oxidative stress, improves cognition
4	Bilobalide $C_{15}H_{18}O_{8}$	*Ginkgo biloba*	Enhances neuroplasticity, protects mitochondria
5	Ginsenoside Rg1 $C_{42}H_{72}O_{14}$	*Panax ginseng*	Neuroprotective, physical stress reliever and stamina enhancer
6	Resveratrol $C_{14}H_{12}O_{3}$	*Vitis vinifera*	Antioxidant, anti-neuroinflammatory and mitigating memory-related impairments
7	Epigallocatechin-3-gallate $C_{22}H_{18}O_{11}$	*Camellia sinensis*	Antioxidant activity
8	Quercetin $C_{15}H_{10}O_{7}$	*Buchanania axillaris and Clitoria ternatea*	Improves cognitive behavior
9	Baicalein $C_{15}H_{10}O_{5}$	*Fuzhisan*	Prevents neuronal death and promotes neurotrophic support
10	Asiaticoside $C_{48}H_{78}O_{19}$	*Centella asiatica* L.	Reduce oxidative stress levels and improves memory and learning
11	Catalpol $C_{15}H_{22}O_{10}$	*Rehmannia glutinosa*	Anti-oxidation, anti-inflammatory and anti-apoptotic
12	Ferulic acid $C_{10}H_{10}O_{4}$	*Ferula asafoetida and Oryza sativa*	Anti-amyloid, antioxidant, protects against memory loss
13	Salvianolic acid $C_{26}H_{22}O_{10}$	*Salvia miltiorrhiza* B.	Anti-oxidative and anti-apoptotic
14	Schisandrin $C_{24}H_{32}O_{7}$	*Alpinia oxyphylla- Schisandra chinensis herb pair*	Enhances mitochondrial function and protects against neurotoxicity
15	Rhynchophylline $C_{22}H_{28}N_{2}O_{4}$	*Uncaria rhynchophylla* M.	Prevents lipid peroxidation and reduce microglial activation
16	Glycyrrhizin $C_{42}H_{62}O_{16}$	*Glycyrrhiza glabra*	Improves spatial learning and memory-enhancing activities
17	γ-linolenic acid $C_{18}H_{30}O_{2}$	*Spirulina platensis*	Anti-inflammatory, reduces cellular toxicity
18	Crocin $C_{44}H_{64}O_{24}$	*Crocus sativus*	Neuroprotective activity targeting tau protein and memory enhancement
19	Isorhamnetin $C_{16}H_{12}O_{7}$	*Viscum album* L.	Reduces the neurotoxic effects
20	Allicin $C_{6}H_{10}OS_{2}$	*Allium sativum* L.	Decreases psychological stress through regulation of stress hormones and the brain’s oxidative stress response
21	Gingerol $C_{17}H_{26}O_{4}$	*Zingiber officinale* R.	Neuroprotective and anti-inflammatory

The field of chemical graph theory offers a powerful approach for understanding molecular structures and their implications in drug development. In this context, molecular graph $G\left(V,E\right)$ serves as visual blueprint, where V represents the set of atoms (vertices) and E represents the set of chemical bonds (edges), respectively (Gutman and Polansky, 2012; Trinajstic, 1992). Two atoms are said to be adjacent if they share a bond, in which case each is called a neighbor of the other. For any atom v∈V , the open neighborhood $N\left(v\right)$ consists of all atoms adjacent to v , while the closed neighborhood $N\left[v\right]$ includes v itself along with its open neighborhood. The degree of an atom v , written deg⁡(v ), is the number of bonds incident to v . Generally, in molecular graphs, double and triple bonds are represented as single edges, which does not affect distance-based graph theoretic metrics but does influence other metrics such as vertex degree. To address this issue, Tamilarasi and Balamurugan (2024) introduced the Isomorphic Molecular Graph, where the bond multiplicity is preserved, that is, single edge for single bond and multiple edges for double and triple bonds. Such graph representations allow molecular structures to be numerically characterized using graph energy (Gutman, 1978), a key spectral descriptor. Graph energy is the sum of the absolute values of the eigenvalues of the adjacency matrix of the graph and it serves as a spectral invariant that reflects the compound’s structural properties. Originating from Hückel molecular orbital (HMO) theory (Gutman, 1977) in quantum chemistry, where it was initially used to approximate the total π-electron energy of conjugated hydrocarbons, graph energy has evolved into a powerful tool in chemical graph theory.

Traditionally, the structural representation of molecular graphs relies on the standard adjacency matrix (Bapat, 2014), a $\left|V\right|\times \left|V\right|$ square matrix, in which each element $a_{ij}$ is 1 if there exists an edge between vertex i and vertex j , and 0 otherwise. For multigraphs, where multiple edges can exist between a pair of vertices as in the case of isomorphic molecular graphs, the adjacency matrix is extended such that $a_{ij}$ represents the number of edges connecting vertex i and vertex j , denoted by $E_{ij}$ ​. While this classical matrix captures structural connectivity, it fails to reflect dominance relationships among the atoms (vertices) in a molecular system. To incorporate this additional layer of information, a Roman domination-based adjacency matrix is proposed, integrating from the concept of Roman domination in graph theory. The concept of Roman domination was introduced by E. J. Cockayne et al. (Cockayne et al., 2004) and it models strategic resource allocation, originally inspired by the defense strategies of the Roman Empire (Henning et al., 2003; ReVelle and Rosing, 2000; Steward, 1999). Roman domination is a graph-theoretic concept that models optimal defense allocation by labeling the vertices with values 0, 1 or 2 such that every vertex labeled 0 is adjacent to at least one vertex labeled 2. A mapping that assigns such labels to the vertices is called a Roman dominating function (RDF). The weight of an RDF is defined as the sum of all vertex labels, and the minimum possible weight over all RDFs is called the Roman domination number, denoted by $\gamma_{R}$ . Throughout this article, only the Roman dominating function that attains minimum weight is considered for all the isomorphic molecular graphs.

Building on this idea, Roman domination-based adjacency matrix $A^{R}\left(G\right)$ is introduced in this paper, which not only retains the structural information of the isomorphic molecular graph but also embeds vertex importance based on the Roman domination function. Consequently, the Roman domination-based graph energy (simply referred to as Roman energy), computed from the eigenvalues of $A^{R}\left(G\right)$ ​, serves as a novel spectral descriptor that captures both connectivity and strategic dominance within the molecular structure. Like graph energy reformulated as Roman energy, other spectral descriptors such as Laplacian energy (Gutman and Zhou, 2006), Randić energy (Bozkurt et al., 2010), Harmonic energy (Hosamani et al., 2017), Atom-Bond Connectivity energy (Prakasha et al., 2024), and Geometric-Arithmetic energy (Hosamani et al., 2017) are also redefined in this study by modifying classical matrix formulations to incorporate Roman domination function values. Roman domination-based graph matrices, namely the Roman Laplacian matrix, Roman Randić matrix, Roman Harmonic matrix, Roman ABC matrix, and Roman Geometric-Arithmetic matrix are introduced along with their corresponding Roman energies. These extended formulations offer deeper insights into molecular topology by integrating both structural and functional aspects of isomorphic molecular graphs.

Motivated by the well-recognized limitations of synthetic drugs, this work benchmarks novel Roman energy descriptors against classical graph energies to demonstrate their superior predictive performance within the herbal compound-based QSPR framework, thereby addressing an existing research gap in Alzheimer’s disease-related QSPR modeling. In this research, the Roman domination number, six classical graph energies and six newly developed Roman energies are computed for 21 anti-Alzheimer’s herbal compounds. QSPR analysis is performed using linear, quadratic and cubic regression models to investigate the relationships between the computed graph energies and physicochemical properties, namely boiling point, enthalpy of vaporization, molar volume, molar refraction, polarizability, polar surface area, molecular weight and surface area. The results demonstrate strong correlations across all metrics using the three regression models. However, the quadratic regression models based on Roman energies show particularly high correlation coefficients accompanied by minimal standard error, indicating their superior predictive performance. The robustness of these significant models is assessed through internal validation methods, including Y-randomization and leave-one-out cross-validation (LOOCV). The best performing models were externally validated by predicting the physicochemical properties of Kaempferol, a phytochemical compound derived from *Corylus avellana*, known to enhance cognitive function, lower anxiety, and mitigate neuroinflammatory and programmed cell death processes in the nervous system (Alsenani, 2024). The prediction yields a high correlation ( r=0.993 ) and minimal root mean squared error ( RMSE=21.25 ) between the experimental and predicted values of Kaempferol’s physicochemical properties, confirming the model’s external reliability. These findings underscore the versatility of the proposed Roman domination-based graph energies, paving the way for their broader application in molecular property prediction, structural analysis and quantitative modeling. Future research may explore their integration into other domains such as materials informatics and complex network analysis, thereby enhancing their utility in theoretical chemistry and computational sciences.

## 2 Motivation

Classical graph matrices such as adjacency and Laplacian matrices, have long been used to represent molecular connectivity, providing a mathematical model for analyzing the structural features of molecules. However, these matrices fail to reflect the varying levels of influence or dominance that certain atoms exert within a molecular structure. While the domination matrices partially address this limitation by identifying the dominated atoms, they still overlook the hierarchical differences in atomic influence. In contrast, the Roman domination function captures an additional layer of information of hierarchical dominance among atoms, where the atoms with RDF value 2 strongly dominate the atoms with RDF value 0, while the atoms with RDF value 1 are self-dominating and are neither dominated by their neighbors nor required to dominate neighboring atoms. Thus, the Roman domination offers a natural way to incorporate functional importance into isomorphic molecular graphs. Motivated by this concept, the present work introduces the Roman domination-based graph energies, which extend the classical graph matrices including the adjacency, Laplacian, Randić, Harmonic, Atom-bond connectivity, and Geometric-arithmetic matrices, by integrating the Roman domination function (RDF) values. These enhanced spectral descriptors are designed to capture both connectivity and dominance-based information simultaneously, offering richer and more informative molecular representations. Ultimately, this approach provides a powerful tool for chemical graphs and molecular modeling, with potential applications in drug design, QSPR/QSAR analysis, and other pharmaceutical research areas where understanding both the structural and functional importance is critical.

## 3 Concepts and terminologies

This section presents the fundamental definitions essential to this research work.

Definition 3.1 (Tamilarasi and Balamurugan, 2024)

Let M be the chemical structure of a molecule. An isomorphic molecular graph $G=\left(V,E\right)$ of M is a graph in which V denotes the set of atoms (vertices) which includes the backbone carbon atoms and heteroatoms, along with the polar hydrogens and hydrogen atoms responsible for stereoisomerism and E denotes the set of chemical bonds (edges) with bond multiplicity preserved; that is, a single edge represents a single bond, two parallel edges represent a double bond and three parallel edges represent a triple bond.

**FIGURE 1:**
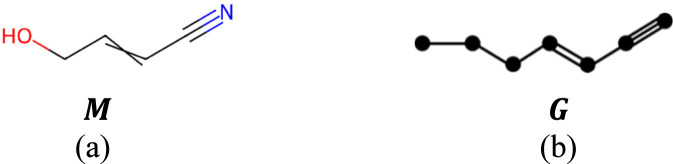
**(a)** Chemical structure and **(b)** isomorphic molecular graph of 4-Hydroxy-2-butenenitrile.

An example of isomorphic molecular graph of 4-Hydroxy-2-butenenitrile is shown in Figure 1.

Definition 3.2 (Cockayne et al., 2004)

Let $G=\left(V,E\right)$ be an isomorphic molecular graph. The Roman dominating function (RDF) on a G is a mapping f:V→0,1,2 that satisfies the condition that every atom (vertex) u∈V with $f\left(u\right)=0$ has at least one neighbor v∈V such that $f\left(v\right)=2$ . The weight of f is defined by $f\left(V\right)=\sum_{u\in V}f\left(u\right)$ . The Roman domination number of G , denoted by $\gamma_{R}\left(G\right)$ , is the minimum weight among all RDFs on G . An RDF f can equivalently be represented as an ordered partition $\left(V_{0},V_{1},V_{2}\right)$ of V , that is, $f=\left(V_{0},V_{1},V_{2}\right)$ , where $V_{i}=\left.\left\{v\in V\;\right.\right|\;f\left(v\right)=i\},i=0,1,2$ . In this notation, the weight of f can be expressed as $f\left(V\right)=\left|V_{1}\right|+2\left|V_{2}\right|$ .

**FIGURE 2:**
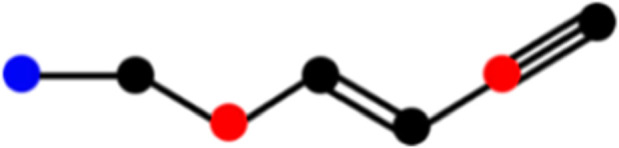
Roman domination of isomorphic molecular graph of 4-Hydroxy-2-butenenitrile (RDF: 2 = red, 1 = blue, 0 = black).

Figure 2 presents an example of Roman domination on the isomorphic molecular graph of 4-Hydroxy-2-butenenitrile G , where vertices with RDF values of 2, 1 and 0 are represented by red, blue and black colors respectively.

Definition 3.3 (Gutman and Polansky, 2012)

For an isomorphic molecular graph $G\left(V,E\right)$ , the adjacency matrix $A\left(G\right)=\left[a_{ij}\right]$ is a $\left|V\right|\times \left|V\right|$ matrix, in which each element $a_{ij}$ is given by

$$a_{ij}=\left\{\begin{array}{l} \left|E_{ij}\right|,\;if\;i\neq j\;\\ 0,\;if\;i=j\; \end{array}\right.$$

where $\left|V\right|$ is the total number of vertices of G and $\left|E_{ij}\right|$ is the number of bonds between the vertices i and j .

Definition 3.4 (Gutman, 1978)

Let $G\left(V,E\right)$ be an isomorphic molecular graph with adjacency matrix $A\left(G\right)$ of order $\left|V\right|$ . Let $\lambda_{1},\lambda_{2},\ldots ,\lambda_{\left|V\right|}$ be the eigenvalues of A . The graph energy of G is defined as the sum of the absolute values of the eigenvalues of $A\left(G\right)$ and it is denoted by $E\left(G\right)$ .That is,

$$E\left(G\right)=\sum_{i=1}^{\left|V\right|}\left|\lambda_{i}\right|$$

Definition 3.5 (Gutman and Zhou, 2006)

Let $G\left(V,E\right)$ be an isomorphic molecular graph. The Laplacian matrix of G , denoted by $L\left(G\right)=\left[l_{ij}\right]$ , is a $\left|V\right|\times \left|V\right|$ matrix, where each element $l_{ij}$ is given by

$$l_{ij}=\left\{\begin{array}{l} \deg\left(i\right),if\;i=j\\ -a_{ij},if\;i\neq j\; \end{array}\right.$$

where $\deg\left(i\right)$ is the number of bonds incident with vertex i and $a_{ij}$ is the $\left(i,j\right)$ -entry of the adjacency matrix.

Definition 3.6 (Gutman and Zhou, 2006)

Laplacian energy of an isomorphic molecular graph $G\left(V,E\right)$ is given by

$$E_{L}\left(G\right)=\left|\sum_{i=1}^{\left|V\right|}{\lambda_{i}}_{L}-\frac{2\left|E\right|}{\left|V\right|}\right|$$

where $\left|V\right|$ is the number of vertices, $\left|E\right|$ is the number of edges and ${\lambda_{i}}_{L}$ denotes the eigenvalues of the Laplacian matrix.

Definition 3.7 (Bozkurt et al., 2010)

Let $G\left(V,E\right)$ be an isomorphic molecular graph. The Randić matrix of G , denoted by $R\left(G\right)=\left[r_{ij}\right]$ , is a $\left|V\right|\times \left|V\right|$ matrix defined by

$$r_{ij}=\left\{\begin{array}{l} \frac{1}{\sqrt{\deg\left(i\right)\times \deg\left(j\right)}},\;if\;i\;and\;j\;are\;adjacent\\ 0,\;otherwise\; \end{array}\right.$$

Definition 3.8 (Bozkurt et al., 2010)

Randić energy of an isomorphic molecular graph $G\left(V,E\right)$ is given by

$$E_{R}\left(G\right)=\sum_{i=1}^{\left|V\right|}\left|\lambda_{i_{R}}\right|$$

where $\lambda_{i_{R}}$ denotes the eigenvalues of the Randić matrix.

Definition 3.9 (Hosamani et al., 2017)

Let $G\left(V,E\right)$ be an isomorphic molecular graph. The Harmonic matrix of G , denoted by $H\left(G\right)=\left[h_{ij}\right]$ , is a $\left|V\right|\times \left|V\right|$ matrix defined by

$$h_{ij}=\left\{\begin{array}{l} \frac{2}{\deg\left(i\right)+\deg\left(j\right)},\;if\;i\;and\;j\;are\;adjacent\\ 0,\;otherwise\; \end{array}\right.$$

Definition 3.10 (Hosamani et al., 2017)

Harmonic energy of an isomorphic molecular graph $G\left(V,E\right)$ is given by

$$E_{H}\left(G\right)=\sum_{i=1}^{\left|V\right|}\left|\lambda_{i_{H}}\right|$$

where $\lambda_{i_{H}}$ denotes the eigenvalues of the Harmonic matrix.

Definition 3.11 (Prakasha et al., 2024)

Let $G\left(V,E\right)$ be an isomorphic molecular graph. The Atom-bond connectivity matrix of G , denoted by $ABC\left(G\right)=\left[c_{ij}\right]$ , is a $\left|V\right|\times \left|V\right|$ matrix defined by

$$c_{ij}=\left\{\begin{array}{l} \sqrt{\frac{\deg\left(i\right)+\deg\left(j\right)-2}{\deg\left(i\right)\times \deg\left(j\right)}},\;if\;i\;and\;j\;are\;adjacent\\ 0,\;otherwise\; \end{array}\right.$$

Definition 3.12 (Prakasha et al., 2024)

Atom-bond connectivity energy of an isomorphic molecular graph $G\left(V,E\right)$ is given by

$$E_{ABC}\left(G\right)=\sum_{i=1}^{\left|V\right|}\left|\lambda_{i_{ABC}}\right|$$

where $\lambda_{i_{ABC}}$ denotes the eigenvalues of the Atom-bond connectivity matrix.

Definition 3.13 (Hosamani et al., 2017)

Let $G\left(V,E\right)$ be an isomorphic molecular graph. The Geometric-Arithmetic matrix of G , denoted by $GA\left(G\right)=\left[g_{ij}\right]$ , is a $\left|V\right|\times \left|V\right|$ matrix defined by

$$g_{ij}=\left\{\begin{array}{l} \frac{2\sqrt{\deg\left(i\right)\times \deg\left(j\right)}}{\deg\left(i\right)+\deg\left(j\right)},\;if\;i\;and\;j\;are\;adjacent\\ 0,\;otherwise\; \end{array}\right.$$

Definition 3.14 (Hosamani et al., 2017)

Geometric-Arithmetic energy of an isomorphic molecular graph $G\left(V,E\right)$ is given by

$$E_{GA}\left(G\right)=\sum_{i=1}^{\left|V\right|}\left|\lambda_{i_{GA}}\right|$$

where $\lambda_{i_{GA}}$ denotes the eigenvalues of the Geometric-Arithmetic matrix.

## 4 Roman domination-based graph energies

This section introduces several novel Roman domination-based matrices and their corresponding graph energy measures.

Definition 4.1

Let $G\left(V,E\right)$ be an isomorphic molecular graph. Then the roman domination-based adjacency matrix $A^{R}\left(G\right)=\left[a_{ij}^{R}\right]$ is a $\left|V\right|\times \left|V\right|$ matrix, in which each element $a_{ij}^{R}$ is defined as

$$a_{ij}^{R}=\left\{\begin{array}{l} f\left(i\right),\;if\;i=j\;\\ \left|E_{ij}\right|+1,\;if\;i\neq j\;and\;f\left(i\right)=2\;\\ \left|E_{ij}\right|,\;ifi\neq jandf\left(i\right)\in 0,1\; \end{array}\right.$$

where $f\left(i\right)$ is the RDF of i .

Definition 4.2

Let $G\left(V,E\right)$ be an isomorphic molecular graph with Roman domination-based adjacency matrix $A^{R}\left(G\right)$ of order $\left|V\right|$ . Let $\lambda_{1}^{R},\lambda_{2}^{R},\ldots ,\lambda_{\left|V\right|}^{R}$ be the eigenvalues of $A^{R}\left(G\right)$ . The Roman energy $E^{R}\left(G\right)$ of G is defined as the sum of the absolute values of the eigenvalues of $A^{R}\left(G\right)$ . That is,

$$E^{R}\left(G\right)=\sum_{i=1}^{\left|V\right|}\left|\lambda_{i}^{R}\right|$$

Definition 4.3

Let $G\left(V,E\right)$ be an isomorphic molecular graph. The Roman Laplacian matrix of G , denoted by $L^{R}\left(G\right)=\left[l_{ij}^{R}\right]$ , is a $\left|V\right|\times \left|V\right|$ matrix defined as

$$l_{ij}^{R}=\left\{\begin{array}{l} \deg\left(i\right),\;if\;i=j\\ -a_{ij}^{R},\;if\;i\neq j\; \end{array}\right.$$

where $\deg\left(i\right)$ is the number of bonds incident with vertex i and $a_{ij}^{R}$ is the $\left(i,j\right)$ -entry of the Roman domination-based adjacency matrix.

Definition 4.4

Roman Laplacian energy of an isomorphic molecular graph $G\left(V,E\right)$ is defined as

$$E_{L}^{R}\left(G\right)=\left|\sum_{i=1}^{\left|V\right|}\lambda_{i_{L}}^{R}-\frac{2\left|E\right|}{\left|V\right|}\right|$$

where $\lambda_{i_{L}}^{R}$ denotes the eigenvalues of the Roman Laplacian matrix.

Definition 4.5

Let $G\left(V,E\right)$ be an isomorphic molecular graph. The Roman Randić matrix of G , denoted by $R^{R}\left(G\right)=\left[r_{ij}^{R}\right]$ , is a $\left|V\right|\times \left|V\right|$ matrix defined as

$$r_{ij}^{R}=\left\{\begin{array}{l} f\left(i\right),\;if\;i=j\;\\ \frac{1}{\sqrt{\deg\left(i\right)\times \deg\left(j\right)}},\;if\;i\neq j\;and\;i\;is\;adjacent\;to\;j\\ 0,\;otherwise\; \end{array}\right.$$

Definition 4.6

Roman Randić energy of an isomorphic molecular graph $G\left(V,E\right)$ is defined as

$$E_{R}^{R}\left(G\right)=\sum_{i=1}^{\left|V\right|}\left|\lambda_{i_{R}}^{R}\right|$$

where $\lambda_{i_{R}}^{R}$ denotes the eigenvalues of the Roman Randić matrix.

Definition 4.7

Let $G\left(V,E\right)$ be an isomorphic molecular graph. The Roman Harmonic matrix of G , denoted by $H^{R}\left(G\right)=\left[h_{ij}^{R}\right]$ , is a $\left|V\right|\times \left|V\right|$ matrix defined as

$$h_{ij}^{R}=\left\{\begin{array}{l} f\left(i\right),\;if\;i=j\;\\ \frac{2}{\deg\left(i\right)+\deg\left(j\right)},\;if\;i\neq j\;and\;i\;is\;adjacent\;to\;j\\ 0,\;otherwise\; \end{array}\right.$$

Definition 4.8

Roman Harmonic energy of an isomorphic molecular graph $G\left(V,E\right)$ is defined as

$$E_{H}^{R}\left(G\right)=\sum_{i=1}^{\left|V\right|}\left|\lambda_{i_{H}}^{R}\right|$$

where $\lambda_{i_{H}}^{R}$ denotes the eigenvalues of the Roman Harmonic matrix.

Definition 4.9

Let $G\left(V,E\right)$ be an isomorphic molecular graph. The Roman Atom-bond connectivity matrix of G , denoted by $ABC^{R}\left(G\right)=\left[c_{ij}^{R}\right]$ , is a $\left|V\right|\times \left|V\right|$ matrix defined by

$$c_{ij}^{R}=\left\{\begin{array}{l} f\left(i\right),\;if\;i=j\;\\ \sqrt{\frac{\deg\left(i\right)+\deg\left(j\right)-2}{\deg\left(i\right)\times \deg\left(j\right)}},\;if\;i\neq j\;and\;i\;is\;adjacent\;to\;j\\ 0,\;otherwise\; \end{array}\right.$$

Definition 4.10

Roman Atom-bond connectivity energy of an isomorphic molecular graph $G\left(V,E\right)$ is given by

$$E_{ABC}^{R}\left(G\right)=\sum_{i=1}^{\left|V\right|}\left|\lambda_{i_{ABC}}^{R}\right|$$

where $\lambda_{i_{ABC}}^{R}$ denotes the eigenvalues of the Roman Atom-bond connectivity matrix.

Definition 4.11

Let $G\left(V,E\right)$ be an isomorphic molecular graph. The Roman Geometric-Arithmetic matrix of G , denoted by $GA^{R}\left(G\right)=\left[g_{ij}^{R}\right]$ , is a $\left|V\right|\times \left|V\right|$ matrix defined as

$$g_{ij}^{R}=\left\{\begin{array}{l} f\left(i\right),\;if\;i=j\;\\ \frac{2\sqrt{\deg\left(i\right)\times \deg\left(j\right)}}{\deg\left(i\right)+\deg\left(j\right)},\;if\;i\neq j\;and\;i\;is\;adjacent\;to\;j\\ 0,\;otherwise\; \end{array}\right.$$

Definition 4.12

Roman Geometric-Arithmetic energy of an isomorphic molecular graph $G\left(V,E\right)$ is defined as

$$E_{GA}^{R}\left(G\right)=\sum_{i=1}^{\left|V\right|}\left|\lambda_{i_{GA}}^{R}\right|$$

where $\lambda_{i_{GA}}^{R}$ denotes the eigenvalues of the Roman Geometric-Arithmetic matrix.

## 5 Methodology

A structured methodology was adopted to investigate the physicochemical properties of herbal anti-Alzheimer’s phytochemicals through Quantitative Structure-Property Relationship (QSPR) graph modeling, utilizing both classical and Roman graph energies derived from the isomorphic molecular graphs of the compounds. The methodology comprises the following steps:

1. Data Acquisition and Construction of Isomorphic Molecular Graph

Potential anti-Alzheimer’s phytochemicals were selected based on their reported neuroprotective efficacy. The molecular structures of these compounds were retrieved from the publicly accessible PubChem database (https://pubchem.ncbi.nlm.nih.gov). Each compound was subsequently represented as an isomorphic molecular graph, where atoms and chemical bonds correspond to vertices and edges respectively.

2. Computation of Classical and Roman Graph Energies

Classical graph energies including, Laplacian, Randic, Harmonic, ABC, and Geometric-Arithmetic energies, and Roman graph energies including Roman Laplacian, Roman Randic, Roman Harmonic, Roman ABC, and Roman Geometric-Arithmetic energies were computed for the constructed isomorphic molecular graphs using theoretical formulations and Python programming. NumPy and SciPy were the python libraries utilized in the computations.

3. QSPR Analysis

QSPR analysis was conducted to examine the relationship between the computed graph energies and the physicochemical properties of the anti-Alzheimer’s compounds using linear, quadratic and cubic regression models. The models were developed with the Statistical Package for the Social Sciences (SPSS) software, enabling quantitative evaluation of model strength and statistical significance.

4. Statistical Evaluation and Model Validation

The predictive performance of the computed graph energies was assessed through multiple statistical measures, including:

- Correlation coefficient ( r ): Indicates the degree of association between predicted and observed values.
- Significance tests ( p ): Ensures the statistical validity of the correlations.
- Standard error values ( S.E. ): Evaluates model accuracy and precision.
- Y-randomization test: Verifies that the observed correlations are not due to random chance.
- Leave-One-Out Cross Validation (LOOCV): Tests the internal stability and reliability of the model.
- External validation through relative error analysis: Confirms external predictive performance on unseen data.

The Roman graph energies demonstrating the highest predictive accuracy and statistical significance were identified as key molecular descriptors, providing a reliable and interpretable model for understanding the structural and physicochemical behavior of anti-Alzheimer’s phytochemicals.

## 6 Computation of classical and Roman energies of anti-Alzheimer’s herbal compounds

The Roman graph energies defined in Section 4 provide a mathematical basis for quantifying both the structural connectivity and the strategic atom importance within isomorphic molecular graphs. In particular, the integration of the Roman domination function into classical and spectral matrices enables the resulting energies to capture not only bond multiplicity and degree-based relationships but also hierarchical dominance patterns among atoms. In this section, the Roman domination number ( $\gamma_{R}$ ), classical graph energies ( $E,E_{L},E_{R},E_{H},E_{ABC}$ and $E_{GA}$ ) and Roman energies ( $E^{R},E_{L}^{R},E_{R}^{R},E_{H}^{R},E_{ABC}^{R}$ and $E_{GA}^{R}$ ) are computed for 21 anti-Alzheimer’s herbal compounds.

Theorem 1

Let $G_{1}\left(V,E\right)$ be the isomorphic molecular graph of the herbal compound Huperzine A with the atom set V and bond set E . Then the graph energies of $G_{1}$ are given by $E\left(G_{1}\right)=34.3620,E_{L}\left(G_{1}\right)=55.3636,E_{R}\left(G_{1}\right)=10.4966,E_{H}\left(G_{1}\right)=9.5596,E_{ABC}\left(G_{1}\right)=19.2852$ and $E_{GA}\left(G_{1}\right)=25.2630$ .

**FIGURE 3:**
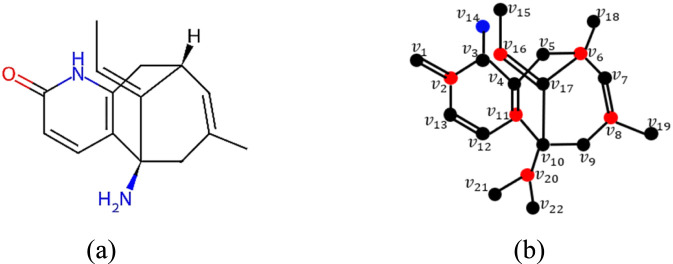
**(a)** Chemical structure and **(b)** Roman domination of isomorphic molecular graph of Huperzine A.

Proof:

Let $G_{1}\left(V,E\right)$ be the isomorphic molecular graph of the herbal compound Huperzine A with $\left|V\right|=22$ and $\left|E\right|=29$ . The chemical structure and its corresponding isomorphic molecular graph of Huperzine A are depicted in Figure 3. Let the atoms of $G_{1}$ be denoted as $v_{1},v_{2},\ldots ,v_{22}$ , as shown in Figure 3b.

The adjacency matrix $A\left(G_{1}\right)$ of $G_{1}$ is obtained as

$$\left[\begin{array}{cccccccccccccccccccccc} \;0 & 2 & 0 & 0 & 0 & 0 & 0 & 0 & 0 & 0 & 0 & 0 & 0 & 0 & 0 & 0 & 0 & 0 & 0 & 0 & 0 & 0\;\\ \;2 & 0 & 1 & 0 & 0 & 0 & 0 & 0 & 0 & 0 & 0 & 0 & 1 & 0 & 0 & 0 & 0 & 0 & 0 & 0 & 0 & 0\;\\ \;0 & 1 & 0 & 1 & 0 & 0 & 0 & 0 & 0 & 0 & 0 & 0 & 0 & 1 & 0 & 0 & 0 & 0 & 0 & 0 & 0 & 0\;\\ \;0 & 0 & 1 & 0 & 1 & 0 & 0 & 0 & 0 & 0 & 2 & 0 & 0 & 0 & 0 & 0 & 0 & 0 & 0 & 0 & 0 & 0\;\\ \;0 & 0 & 0 & 1 & 0 & 1 & 0 & 0 & 0 & 0 & 0 & 0 & 0 & 0 & 0 & 0 & 0 & 0 & 0 & 0 & 0 & 0\;\\ \;0 & 0 & 0 & 0 & 1 & 0 & 1 & 0 & 0 & 0 & 0 & 0 & 0 & 0 & 0 & 0 & 1 & 1 & 0 & 0 & 0 & 0\;\\ \;0 & 0 & 0 & 0 & 0 & 1 & 0 & 2 & 0 & 0 & 0 & 0 & 0 & 0 & 0 & 0 & 0 & 0 & 0 & 0 & 0 & 0\;\\ \;0 & 0 & 0 & 0 & 0 & 0 & 2 & 0 & 1 & 0 & 0 & 0 & 0 & 0 & 0 & 0 & 0 & 0 & 1 & 0 & 0 & 0\;\\ \;0 & 0 & 0 & 0 & 0 & 0 & 0 & 1 & 0 & 1 & 0 & 0 & 0 & 0 & 0 & 0 & 0 & 0 & 0 & 0 & 0 & 0\;\\ \;0 & 0 & 0 & 0 & 0 & 0 & 0 & 0 & 1 & 0 & 1 & 0 & 0 & 0 & 0 & 0 & 1 & 0 & 0 & 1 & 0 & 0\;\\ \;0 & 0 & 0 & 2 & 0 & 0 & 0 & 0 & 0 & 1 & 0 & 1 & 0 & 0 & 0 & 0 & 0 & 0 & 0 & 0 & 0 & 0\;\\ \;0 & 0 & 0 & 0 & 0 & 0 & 0 & 0 & 0 & 0 & 1 & 0 & 2 & 0 & 0 & 0 & 0 & 0 & 0 & 0 & 0 & 0\;\\ \;0 & 1 & 0 & 0 & 0 & 0 & 0 & 0 & 0 & 0 & 0 & 2 & 0 & 0 & 0 & 0 & 0 & 0 & 0 & 0 & 0 & 0\;\\ \;0 & 0 & 1 & 0 & 0 & 0 & 0 & 0 & 0 & 0 & 0 & 0 & 0 & 0 & 0 & 0 & 0 & 0 & 0 & 0 & 0 & 0\;\\ \;0 & 0 & 0 & 0 & 0 & 0 & 0 & 0 & 0 & 0 & 0 & 0 & 0 & 0 & 0 & 1 & 0 & 0 & 0 & 0 & 0 & 0\;\\ \;0 & 0 & 0 & 0 & 0 & 0 & 0 & 0 & 0 & 0 & 0 & 0 & 0 & 0 & 1 & 0 & 2 & 0 & 0 & 0 & 0 & 0\;\\ \;0 & 0 & 0 & 0 & 0 & 1 & 0 & 0 & 0 & 1 & 0 & 0 & 0 & 0 & 0 & 2 & 0 & 0 & 0 & 0 & 0 & 0\;\\ \;0 & 0 & 0 & 0 & 0 & 1 & 0 & 0 & 0 & 0 & 0 & 0 & 0 & 0 & 0 & 0 & 0 & 0 & 0 & 0 & 0 & 0\;\\ \;0 & 0 & 0 & 0 & 0 & 0 & 0 & 1 & 0 & 0 & 0 & 0 & 0 & 0 & 0 & 0 & 0 & 0 & 0 & 0 & 0 & 0\;\\ \;0 & 0 & 0 & 0 & 0 & 0 & 0 & 0 & 0 & 1 & 0 & 0 & 0 & 0 & 0 & 0 & 0 & 0 & 0 & 0 & 1 & 1\;\\ \;0 & 0 & 0 & 0 & 0 & 0 & 0 & 0 & 0 & 0 & 0 & 0 & 0 & 0 & 0 & 0 & 0 & 0 & 0 & 1 & 0 & 0\;\\ \;0 & 0 & 0 & 0 & 0 & 0 & 0 & 0 & 0 & 0 & 0 & 0 & 0 & 0 & 0 & 0 & 0 & 0 & 0 & 1 & 0 & 0\; \end{array}\right]$$

The characteristic equation of $A\left(G_{1}\right)$ is $\left|A\left(G_{1}\right)-\lambda I\right|=0$ , where I is the unit matrix of order 22. That is,

$$\lambda^{22}-\left(1.75745637\times 10^{-15}\right)\lambda^{21}-39\lambda^{20}+\left(3.83427322\times 10^{-14}\right)\lambda^{19}+630\lambda^{18}-\left(5.56526893\times 10^{-13}\right)\lambda^{17}-5491\lambda^{16}+\left(1.71520693\times 10^{-12}\right)\lambda^{15}+28171\lambda^{14}-\left(3.10553370\times 10^{-12}\right)\lambda^{13}-86959\lambda^{12}-\left(4.52993115\times 10^{-11}\right)\lambda^{11}+158108\lambda^{10}+\left(1.19403637\times 10^{-10}\right)\lambda^{9}-158194\lambda^{8}-\left(4.66456864\times 10^{-11}\right)\lambda^{7}+75767\lambda^{6}+\left(1.59839470\times 10^{-11}\right)\lambda^{5}-12918\lambda^{4}-\left(2.44156254\times 10^{-13}\right)\lambda^{3}-\left(7.24824964\times 10^{-30}\right)\lambda^{2}+\left(1.21946898\times 10^{-62}\right)\lambda +\left(2.17816785\times 10^{-101}\right)=0$$

Now, the eigenvalues ${\left\{\lambda_{i}\right\}}_{i=1}^{22}$ of $A\left(G_{1}\right)$ are computed as −3.22168250, 3.22168250, −2.86380483, 2.86380483, −2.42094968, −2.25231345, −1.88326844, −1.76307891, 2.42094968, 2.25231345, 1.88326844, 1.76307891, −1.31844167, −0.851087705, −0.606369954, 1.31844167, 0.851087705, 0.606369954, ​(−9.45023434 × 10^−18^ + 2.17207266 × 10^−17^i​), (−9.45023434 × 10^−18^−2.17207266 × 10^−17^i), 1.68243416 × 10^−33^ and −1.78615903 × 10^−39^.

Hence, the graph energy $E\left(G_{1}\right)$ of $G_{1}$ is

$$E\left(G_{1}\right)=\sum_{i=1}^{\left|V\right|}\left|\lambda_{i}\right|=\sum_{i=1}^{22}\left|\lambda_{i}\right|$$

$$=|-3.22168250|+|3.22168250|+|-2.86380483|+|2.86380483|+|-2.42094968|+|-2.25231345|+|-1.88326844|+|-1.76307891|+|2.42094968|+|2.25231345|+|1.88326844|+|1.76307891|+|-1.31844167|+|-0.851087705|+|-0.606369954|+|1.31844167|+|0.851087705|+|0.606369954\;|+\left|-9.45023434\times 10^{-18}+2.17207266\times 10^{-17}i\right|+\left|-9.45023434\times 10^{-18}-2.17207266\times 10^{-17}i\right|+|1.68243416\times 10^{-33}|+|-1.78615903\times 10^{-39}\left|=34.3620\;\right.$$

Similarly, the Laplacian matrix $L\left(G_{1}\right)$ of $G_{1}$ is obtained as

$$\left[\begin{array}{cccccccccccccccccccccc} \;2 & -2 & \;0 & \;0 & \;0 & \;0 & \;0 & \;0 & \;0 & \;0 & \;0 & \;0 & \;0 & \;0 & \;0 & \;0 & \;0 & \;0 & \;0 & \;0 & \;0 & \;0\;\\ -2 & \;4 & -1 & \;0 & \;0 & \;0 & \;0 & \;0 & \;0 & \;0 & \;0 & \;0 & -1 & \;0 & \;0 & \;0 & \;0 & \;0 & \;0 & \;0 & \;0 & \;0\;\\ \;0 & -1 & \;3 & -1 & \;0 & \;0 & \;0 & \;0 & \;0 & \;0 & \;0 & \;0 & \;0 & -1 & \;0 & \;0 & \;0 & \;0 & \;0 & \;0 & \;0 & \;0\;\\ \;0 & \;0 & -1 & \;4 & -1 & \;0 & \;0 & \;0 & \;0 & \;0 & -2 & \;0 & \;0 & \;0 & \;0 & \;0 & \;0 & \;0 & \;0 & \;0 & \;0 & \;0\;\\ \;0 & \;0 & \;0 & -1 & \;2 & -1 & \;0 & \;0 & \;0 & \;0 & \;0 & \;0 & \;0 & \;0 & \;0 & \;0 & \;0 & \;0 & \;0 & \;0 & \;0 & \;0\;\\ \;0 & \;0 & \;0 & \;0 & -1 & \;4 & -1 & \;0 & \;0 & \;0 & \;0 & \;0 & \;0 & \;0 & \;0 & \;0 & -1 & -1 & \;0 & \;0 & \;0 & \;0\;\\ \;0 & \;0 & \;0 & \;0 & \;0 & -1 & \;3 & -2 & \;0 & \;0 & \;0 & \;0 & \;0 & \;0 & \;0 & \;0 & \;0 & \;0 & \;0 & \;0 & \;0 & \;0\;\\ \;0 & \;0 & \;0 & \;0 & \;0 & \;0 & -2 & \;4 & -1 & \;0 & \;0 & \;0 & \;0 & \;0 & \;0 & \;0 & \;0 & \;0 & -1 & \;0 & \;0 & \;0\;\\ \;0 & \;0 & \;0 & \;0 & \;0 & \;0 & \;0 & -1 & \;2 & -1 & \;0 & \;0 & \;0 & \;0 & \;0 & \;0 & \;0 & \;0 & \;0 & \;0 & \;0 & \;0\;\\ \;0 & \;0 & \;0 & \;0 & \;0 & \;0 & \;0 & \;0 & -1 & \;4 & -1 & \;0 & \;0 & \;0 & \;0 & \;0 & -1 & \;0 & \;0 & -1 & \;0 & \;0\;\\ \;0 & \;0 & \;0 & -2 & \;0 & \;0 & \;0 & \;0 & \;0 & -1 & \;4 & -1 & \;0 & \;0 & \;0 & \;0 & \;0 & \;0 & \;0 & \;0 & \;0 & \;0\;\\ \;0 & \;0 & \;0 & \;0 & \;0 & \;0 & \;0 & \;0 & \;0 & \;0 & -1 & \;3 & -2 & \;0 & \;0 & \;0 & \;0 & \;0 & \;0 & \;0 & \;0 & \;0\;\\ \;0 & -1 & \;0 & \;0 & \;0 & \;0 & \;0 & \;0 & \;0 & \;0 & \;0 & -2 & \;3 & \;0 & \;0 & \;0 & \;0 & \;0 & \;0 & \;0 & \;0 & \;0\;\\ \;0 & \;0 & -1 & \;0 & \;0 & \;0 & \;0 & \;0 & \;0 & \;0 & \;0 & \;0 & \;0 & \;1 & \;0 & \;0 & \;0 & \;0 & \;0 & \;0 & \;0 & \;0\;\\ \;0 & \;0 & \;0 & \;0 & \;0 & \;0 & \;0 & \;0 & \;0 & \;0 & \;0 & \;0 & \;0 & \;0 & \;1 & -1 & \;0 & \;0 & \;0 & \;0 & \;0 & \;0\;\\ \;0 & \;0 & \;0 & \;0 & \;0 & \;0 & \;0 & \;0 & \;0 & \;0 & \;0 & \;0 & \;0 & \;0 & -1 & \;3 & -2 & \;0 & \;0 & \;0 & \;0 & \;0\;\\ \;0 & \;0 & \;0 & \;0 & \;0 & -1 & \;0 & \;0 & \;0 & -1 & \;0 & \;0 & \;0 & \;0 & \;0 & -2 & \;4 & \;0 & \;0 & \;0 & \;0 & \;0\;\\ \;0 & \;0 & \;0 & \;0 & \;0 & -1 & \;0 & \;0 & \;0 & \;0 & \;0 & \;0 & \;0 & \;0 & \;0 & \;0 & \;0 & \;1 & \;0 & \;0 & \;0 & \;0\;\\ \;0 & \;0 & \;0 & \;0 & \;0 & \;0 & \;0 & -1 & \;0 & \;0 & \;0 & \;0 & \;0 & \;0 & \;0 & \;0 & \;0 & \;0 & \;1 & \;0 & \;0 & \;0\;\\ \;0 & \;0 & \;0 & \;0 & \;0 & \;0 & \;0 & \;0 & \;0 & -1 & \;0 & \;0 & \;0 & \;0 & \;0 & \;0 & \;0 & \;0 & \;0 & \;3 & -1 & -1\;\\ \;0 & \;0 & \;0 & \;0 & \;0 & \;0 & \;0 & \;0 & \;0 & \;0 & \;0 & \;0 & \;0 & \;0 & \;0 & \;0 & \;0 & \;0 & \;0 & -1 & \;1 & \;0\;\\ \;0 & \;0 & \;0 & \;0 & \;0 & \;0 & \;0 & \;0 & \;0 & \;0 & \;0 & \;0 & \;0 & \;0 & \;0 & \;0 & \;0 & \;0 & \;0 & -1 & \;0 & \;1\; \end{array}\right]$$

The characteristic equation of $L\left(G_{1}\right)$ is $\left|L\left(G_{1}\right)-\lambda I\right|=0$ , where I is the unit matrix of order 22. That is,

$$\lambda^{22}-58\lambda^{21}+1551\lambda^{20}-25386\lambda^{19}+284755\lambda^{18}-2322934\lambda^{17}+14275283\lambda^{16}-67559562\lambda^{15}+249672537\lambda^{14}-726496146\lambda^{13}+1671245190\lambda^{12}-3041159280\lambda^{11}+4366013530\lambda^{10}-4916017360\lambda^{9}+4299930840\lambda^{8}-2880690370\lambda^{7}+1448608970\lambda^{6}-531177794\lambda^{5}+136056563\lambda^{4}-22745186\lambda^{3}+2196274\lambda^{2}-91432\lambda +\left(3.98590417\times 10^{-11}\right)=0$$

The eigenvalues ${\left\{\lambda_{i_{L}}\right\}}_{i=1}^{22}$ of $L\left(G_{1}\right)$ are 6.88478479, 6.35915857, 5.87817418, 5.75183678, 5.05688787, 4.78859112, 4.28246298, 3.39370494, 3.07416394, 2.44193559, 1.88110879, 1.77487523, 1.34213456, 1.22273060, 4.35941921 × 10^−16^, 0.895980521, 0.151978808, 0.234847803, 0.643389696, 0.589366431, 0.351886823 and 1.

The corresponding Laplacian energy $E_{L}\left(G_{1}\right)$ is

$$E_{L}\left(G_{1}\right)=\left|\sum_{i=1}^{\left|V\right|}\lambda_{i_{L}}-\frac{2\left|E\right|}{\left|V\right|}\right|=\left|\sum_{i=1}^{22}\lambda_{i_{L}}-\frac{2\left(29\right)}{22}\right|$$

$$=\left|\left[6.88478479+6.35915857+5.87817418+5.75183678+5.05688787+4.78859112+4.28246298+3.39370494+3.07416394+2.44193559+1.88110879+1.77487523+1.34213456+1.22273060+\left(4.35941921\times 10^{-16}\right)+0.895980521+0.151978808+0.234847803+0.643389696+0.589366431+0.351886823+1\right]-\left(\frac{2\left(29\right)}{22}\right)\right|=\left|58-\left(\frac{58}{22}\right)\right|=55.3636$$

The Randić matrix $R\left(G_{1}\right)$ of $G_{1}$ is obtained as

$$\left[\begin{array}{cccccccccccccccccccccc} \;0 & \;0.35 & \;0 & \;0 & \;0 & \;0 & \;0 & \;0 & \;0 & \;0 & \;0 & \;0 & \;0 & \;0 & \;0 & \;0 & \;0 & \;0 & \;0 & \;0 & \;0 & \;0\;\\ \;0.35 & \;0 & \;0.29 & \;0 & \;0 & \;0 & \;0 & \;0 & \;0 & \;0 & \;0 & \;0 & \;0.29 & \;0 & \;0 & \;0 & \;0 & \;0 & \;0 & \;0 & \;0 & \;0\\ \;0 & \;0.29 & \;0 & \;0.29 & \;0 & \;0 & \;0 & \;0 & \;0 & \;0 & \;0 & \;0 & \;0 & \;0.58 & \;0 & \;0 & \;0 & \;0 & \;0 & \;0 & \;0 & \;0\\ \;0 & \;0 & \;0.29 & \;0 & \;0.35 & \;0 & \;0 & \;0 & \;0 & \;0 & \;0.25 & \;0 & \;0 & \;0 & \;0 & \;0 & \;0 & \;0 & \;0 & \;0 & \;0 & \;0\\ \;0 & \;0 & \;0 & \;0.35 & \;0 & \;0.35 & \;0 & \;0 & \;0 & \;0 & \;0 & \;0 & \;0 & \;0 & \;0 & \;0 & \;0 & \;0 & \;0 & \;0 & \;0 & \;0\\ \;0 & \;0 & \;0 & \;0 & \;0.35 & \;0 & \;0.29 & \;0 & \;0 & \;0 & \;0 & \;0 & \;0 & \;0 & \;0 & \;0 & \;0.25 & \;0.50 & \;0 & \;0 & \;0 & \;0\\ \;0 & \;0 & \;0 & \;0 & \;0 & \;0.29 & \;0 & \;0.29 & \;0 & \;0 & \;0 & \;0 & \;0 & \;0 & \;0 & \;0 & \;0 & \;0 & \;0 & \;0 & \;0 & \;0\\ \;0 & \;0 & \;0 & \;0 & \;0 & \;0 & \;0.29 & \;0 & \;0.35 & \;0 & \;0 & \;0 & \;0 & \;0 & \;0 & \;0 & \;0 & \;0 & \;0.50 & \;0 & \;0 & \;0\\ \;0 & \;0 & \;0 & \;0 & \;0 & \;0 & \;0 & \;0.35 & \;0 & \;0.35 & \;0 & \;0 & \;0 & \;0 & \;0 & \;0 & \;0 & \;0 & \;0 & \;0 & \;0 & \;0\\ \;0 & \;0 & \;0 & \;0 & \;0 & \;0 & \;0 & \;0 & \;0.35 & \;0 & \;0.25 & \;0 & \;0 & \;0 & \;0 & \;0 & \;0.25 & \;0 & \;0 & \;0.29 & \;0 & \;0\\ \;0 & \;0 & \;0 & \;0.25 & \;0 & \;0 & \;0 & \;0 & \;0 & \;0.25 & \;0 & \;0.29 & \;0 & \;0 & \;0 & \;0 & \;0 & \;0 & \;0 & \;0 & \;0 & \;0\\ \;0 & \;0 & \;0 & \;0 & \;0 & \;0 & \;0 & \;0 & \;0 & \;0 & \;0.29 & \;0 & \;0.33 & \;0 & \;0 & \;0 & \;0 & \;0 & \;0 & \;0 & \;0 & \;0\\ \;0 & \;0.29 & \;0 & \;0 & \;0 & \;0 & \;0 & \;0 & \;0 & \;0 & \;0 & \;0.33 & \;0 & \;0 & \;0 & \;0 & \;0 & \;0 & \;0 & \;0 & \;0 & \;0\\ \;0 & \;0 & \;0.58 & \;0 & \;0 & \;0 & \;0 & \;0 & \;0 & \;0 & \;0 & \;0 & \;0 & \;0 & \;0 & \;0 & \;0 & \;0 & \;0 & \;0 & \;0 & \;0\\ \;0 & \;0 & \;0 & \;0 & \;0 & \;0 & \;0 & \;0 & \;0 & \;0 & \;0 & \;0 & \;0 & \;0 & \;0 & \;0.58 & \;0 & \;0 & \;0 & \;0 & \;0 & \;0\\ \;0 & \;0 & \;0 & \;0 & \;0 & \;0 & \;0 & \;0 & \;0 & \;0 & \;0 & \;0 & \;0 & \;0 & \;0.58 & \;0 & \;0.29 & \;0 & \;0 & \;0 & \;0 & \;0\\ \;0 & \;0 & \;0 & \;0 & \;0 & \;0.25 & \;0 & \;0 & \;0 & \;0.25 & \;0 & \;0 & \;0 & \;0 & \;0 & \;0.29 & \;0 & \;0 & \;0 & \;0 & \;0 & \;0\\ \;0 & \;0 & \;0 & \;0 & \;0 & \;0.50 & \;0 & \;0 & \;0 & \;0 & \;0 & \;0 & \;0 & \;0 & \;0 & \;0 & \;0 & \;0 & \;0 & \;0 & \;0 & \;0\\ \;0 & \;0 & \;0 & \;0 & \;0 & \;0 & \;0 & \;0.50 & \;0 & \;0 & \;0 & \;0 & \;0 & \;0 & \;0 & \;0 & \;0 & \;0 & \;0 & \;0 & \;0 & \;0\\ \;0 & \;0 & \;0 & \;0 & \;0 & \;0 & \;0 & \;0 & \;0 & \;0.29 & \;0 & \;0 & \;0 & \;0 & \;0 & \;0 & \;0 & \;0 & \;0 & \;0 & \;0.58 & \;0.58\\ \;0 & \;0 & \;0 & \;0 & \;0 & \;0 & \;0 & \;0 & \;0 & \;0 & \;0 & \;0 & \;0 & \;0 & \;0 & \;0 & \;0 & \;0 & \;0 & \;0.58 & \;0 & \;0\\ \;0 & \;0 & \;0 & \;0 & \;0 & \;0 & \;0 & \;0 & \;0 & \;0 & \;0 & \;0 & \;0 & \;0 & \;0 & \;0 & \;0 & \;0 & \;0 & \;0.58 & \;0 & \;0 \end{array}\right]$$

The characteristic equation of $R\left(G_{1}\right)$ is $\left|R\left(G_{1}\right)-\lambda I\right|=0$ , where I is the unit matrix of order 22. That is,

$$\lambda^{22}-\left(1.81022563\times 10^{-15}\right)\lambda^{21}-3.48611111\lambda^{20}+\left(1.03606488\times 10^{-14}\right)\lambda^{19}+5.12340856\lambda^{18}-\left(2.58683361\times 10^{-14}\right)\lambda^{17}-4.14134235\lambda^{16}-\left(1.60768400\times 10^{-14}\right)\lambda^{15}+2.01350911\lambda^{14}-\left(2.28789127\times 10^{-15}\right)\lambda^{13}-0.604601071\lambda^{12}+\left(2.31838336\times 10^{-15}\right)\lambda^{11}+0.110653285\lambda^{10}+\left(2.66809266\times 10^{-17}\right)\lambda^{9}-\left(1.16928582\times 10^{-2}\right)\lambda^{8}-\left(4.69901450\times 10^{-19}\right)\lambda^{7}+\left(6.31879223\times 10^{-4}\right)\lambda^{6}-\left(1.01521125\times 10^{-19}\right)\lambda^{5}-\left(1.29563655\times 10^{-5}\right)\lambda^{4}-\left(2.80406292\times 10^{-22}\right)\lambda^{3}+\left(1.00557007\times 10^{-40}\right)\lambda^{2}+\left(1.54932601\times 10^{-73}\right)\lambda +0=0$$

The eigenvalues ${\left\{\lambda_{i_{R}}\right\}}_{i=1}^{22}$ of $R\left(G_{1}\right)$ are −0.908485608, −0.815451090, −0.753219975, −0.664961047, −0.621203066, −0.506456697, −0.443479161, −0.312720323, −0.222332197, 0.908485608, 0.815451090, 0.753219975, 0.664961047, 0.621203066, 0.222332197, 0.312720323, 0.506456697, 0.443479161, −1.54074396 × 10^−33^, −2.19952161 × 10^−17^, 3.52858770 × 10^−19^ and 0

The corresponding Randić energy $E_{R}\left(G_{1}\right)$ is

$$E_{R}\left(G_{1}\right)=\sum_{i=1}^{\left|V\right|}\left|\lambda_{i_{R}}\right|=\sum_{i=1}^{22}\left|\lambda_{i_{R}}\right|$$

$$=\left|-0.908485608\right|+\left|-0.815451090\right|+\left|-0.753219975\right|+\left|-0.664961047\right|+\left|-0.621203066\right|+\left|-0.506456697\right|+\left|-0.443479161\right|+\left|-0.312720323\right|+\left|-0.222332197\right|+\left|0.908485608\right|+\left|0.815451090\right|+\left|0.753219975\right|+\left|0.664961047\right|+\left|0.621203066\right|+\left|0.222332197\right|+\left|0.312720323\right|+\left|0.506456697\right|+\left|0.443479161\right|+\left|-1.54074396\times 10^{-33}\right|+\left|-2.19952161\times 10^{-17}\right|+\left|3.52858770\times 10^{-19}\right|+\left|0\right|=10.4966$$

Similarly, the harmonic matrix $H\left(G_{1}\right)$ of $G_{1}$ is obtained as

$$\left[\begin{array}{cccccccccccccccccccccc} \;0 & \;0.35 & \;0 & \;0 & \;0 & \;0 & \;0 & \;0 & \;0 & \;0 & \;0 & \;0 & \;0 & \;0 & \;0 & \;0 & \;0 & \;0 & \;0 & \;0 & \;0 & \;0\;\\ \;0.35 & \;0 & \;0.29 & \;0 & \;0 & \;0 & \;0 & \;0 & \;0 & \;0 & \;0 & \;0 & \;0.29 & \;0 & \;0 & \;0 & \;0 & \;0 & \;0 & \;0 & \;0 & \;0\\ \;0 & \;0.29 & \;0 & \;0.29 & \;0 & \;0 & \;0 & \;0 & \;0 & \;0 & \;0 & \;0 & \;0 & \;0.50 & \;0 & \;0 & \;0 & \;0 & \;0 & \;0 & \;0 & \;0\\ \;0 & \;0 & \;0.29 & \;0 & \;0.33 & \;0 & \;0 & \;0 & \;0 & \;0 & \;0.25 & \;0 & \;0 & \;0 & \;0 & \;0 & \;0 & \;0 & \;0 & \;0 & \;0 & \;0\\ \;0 & \;0 & \;0 & \;0.33 & \;0 & \;0.33 & \;0 & \;0 & \;0 & \;0 & \;0 & \;0 & \;0 & \;0 & \;0 & \;0 & \;0 & \;0 & \;0 & \;0 & \;0 & \;0\\ \;0 & \;0 & \;0 & \;0 & \;0.33 & \;0 & \;0.29 & \;0 & \;0 & \;0 & \;0 & \;0 & \;0 & \;0 & \;0 & \;0 & \;0.25 & \;0.40 & \;0 & \;0 & \;0 & \;0\\ \;0 & \;0 & \;0 & \;0 & \;0 & \;0.29 & \;0 & \;0.29 & \;0 & \;0 & \;0 & \;0 & \;0 & \;0 & \;0 & \;0 & \;0 & \;0 & \;0 & \;0 & \;0 & \;0\\ \;0 & \;0 & \;0 & \;0 & \;0 & \;0 & \;0.29 & \;0 & \;0.33 & \;0 & \;0 & \;0 & \;0 & \;0 & \;0 & \;0 & \;0 & \;0 & \;0.40 & \;0 & \;0 & \;0\\ \;0 & \;0 & \;0 & \;0 & \;0 & \;0 & \;0 & \;0.33 & \;0 & \;0.33 & \;0 & \;0 & \;0 & \;0 & \;0 & \;0 & \;0 & \;0 & \;0 & \;0 & \;0 & \;0\\ \;0 & \;0 & \;0 & \;0 & \;0 & \;0 & \;0 & \;0 & \;0.33 & \;0 & \;0.25 & \;0 & \;0 & \;0 & \;0 & \;0 & \;0.25 & \;0 & \;0 & \;0.29 & \;0 & \;0\\ \;0 & \;0 & \;0 & \;0.25 & \;0 & \;0 & \;0 & \;0 & \;0 & \;0.25 & \;0 & \;0.29 & \;0 & \;0 & \;0 & \;0 & \;0 & \;0 & \;0 & \;0 & \;0 & \;0\\ \;0 & \;0 & \;0 & \;0 & \;0 & \;0 & \;0 & \;0 & \;0 & \;0 & \;0.29 & \;0 & \;0.33 & \;0 & \;0 & \;0 & \;0 & \;0 & \;0 & \;0 & \;0 & \;0\\ \;0 & \;0.29 & \;0 & \;0 & \;0 & \;0 & \;0 & \;0 & \;0 & \;0 & \;0 & \;0.33 & \;0 & \;0 & \;0 & \;0 & \;0 & \;0 & \;0 & \;0 & \;0 & \;0\\ \;0 & \;0 & \;0.50 & \;0 & \;0 & \;0 & \;0 & \;0 & \;0 & \;0 & \;0 & \;0 & \;0 & \;0 & \;0 & \;0 & \;0 & \;0 & \;0 & \;0 & \;0 & \;0\\ \;0 & \;0 & \;0 & \;0 & \;0 & \;0 & \;0 & \;0 & \;0 & \;0 & \;0 & \;0 & \;0 & \;0 & \;0 & \;0.50 & \;0 & \;0 & \;0 & \;0 & \;0 & \;0\\ \;0 & \;0 & \;0 & \;0 & \;0 & \;0 & \;0 & \;0 & \;0 & \;0 & \;0 & \;0 & \;0 & \;0 & \;0.50 & \;0 & \;0.29 & \;0 & \;0 & \;0 & \;0 & \;0\\ \;0 & \;0 & \;0 & \;0 & \;0 & \;0.25 & \;0 & \;0 & \;0 & \;0.25 & \;0 & \;0 & \;0 & \;0 & \;0 & \;0.29 & \;0 & \;0 & \;0 & \;0 & \;0 & \;0\\ \;0 & \;0 & \;0 & \;0 & \;0 & \;0.40 & \;0 & \;0 & \;0 & \;0 & \;0 & \;0 & \;0 & \;0 & \;0 & \;0 & \;0 & \;0 & \;0 & \;0 & \;0 & \;0\\ \;0 & \;0 & \;0 & \;0 & \;0 & \;0 & \;0 & \;0.40 & \;0 & \;0 & \;0 & \;0 & \;0 & \;0 & \;0 & \;0 & \;0 & \;0 & \;0 & \;0 & \;0 & \;0\\ \;0 & \;0 & \;0 & \;0 & \;0 & \;0 & \;0 & \;0 & \;0 & \;0.29 & \;0 & \;0 & \;0 & \;0 & \;0 & \;0 & \;0 & \;0 & \;0 & \;0 & \;0.50 & \;0.50\\ \;0 & \;0 & \;0 & \;0 & \;0 & \;0 & \;0 & \;0 & \;0 & \;0 & \;0 & \;0 & \;0 & \;0 & \;0 & \;0 & \;0 & \;0 & \;0 & \;0.50 & \;0 & \;0\\ \;0 & \;0 & \;0 & \;0 & \;0 & \;0 & \;0 & \;0 & \;0 & \;0 & \;0 & \;0 & \;0 & \;0 & \;0 & \;0 & \;0 & \;0 & \;0 & \;0.50 & \;0 & \;0 \end{array}\right]$$

The characteristic equation of $H\left(G_{1}\right)$ is $\left|H\left(G_{1}\right)-\lambda I\right|=0$ , where I is the unit matrix of order 22. That is,

$$\lambda^{22}+\left(3.12474908\times 10^{-15}\right)\lambda^{21}-2.88972789\lambda^{20}-\left(7.81815692\times 10^{-15}\;\right)\lambda^{19}+3.51938148\lambda^{18}+\left(1.33794250\times 10^{-14}\right)\lambda^{17}-2.35817339\lambda^{16}-\left(1.26945671\times 10^{-14}\right)\lambda^{15}+0.951404041\lambda^{14}-\left(1.42794891\times 10^{-15}\right)\lambda^{13}-0.237456295\lambda^{12}+\left(1.76582350\times 10^{-16}\right)\lambda^{11}+0.0361837626\lambda^{10}+\left(4.56862596\times 10^{-17}\right)\lambda^{9}-0.00318405536\lambda^{8}-\left(1.84027525\times 10^{-18}\right)\lambda^{7}+\left(1.42773684\times 10^{-4}\right)\lambda^{6}-\left(1.80266200\times 10^{-20}\right)\lambda^{5}-\left(2.41270721\times 10^{-6}\right)\lambda^{4}-\left(3.89039754\times 10^{-23}\right)\lambda^{3}+\left(9.55111219\times 10^{-41}\right)\lambda^{2}+\left(2.94316367\times 10^{-73}\right)\lambda -\left(1.57327680\times 10^{-123}\right)=0$$

The eigenvalues ${\left\{\lambda_{i_{H}}\right\}}_{i=1}^{22}$ of $H\left(G_{1}\right)$ are −0.832107140, −0.743531733, −0.690959869, −0.586425446, −0.547457484, −0.420175763, −0.476460052, −0.282647751, −0.200011284, 0.832107140, 0.743531733, 0.690959869, 0.200011284, 0.282647751, 0.586425446, 0.547457484, 0.420175763, 0.476460052, −3.08148791 × 10^−33^, −1.82891110 × 10^−17^, 2.16449571 × 10^−18^ and 5.34552942 × 10^−51^.

Hence, the harmonic energy $E_{H}\left(G_{1}\right)$ is

$$E_{H}\left(G_{1}\right)=\sum_{i=1}^{\left|V\right|}\left|\lambda_{i_{H}}\right|=\sum_{i=1}^{22}\left|\lambda_{i_{H}}\right|$$

$$=\left|-0.832107140\right|+\left|-0.743531733\right|+\left|-0.690959869\right|+\left|-0.586425446\right|+\left|-0.547457484\right|+\left|-0.420175763\right|+\left|-0.476460052\right|+\left|-0.282647751\right|+\left|-0.200011284\right|+\left|0.832107140\right|+\left|0.743531733\right|+\left|0.690959869\right|+\left|0.200011284\right|+\left|0.282647751\right|+\left|0.586425446\right|+\left|0.547457484\right|+\left|0.420175763\right|+\left|0.476460052\right|+\left|-3.08148791\times 10^{-33}\right|+\left|-1.82891110\times 10^{-17}\right|+\left|2.16449571\times 10^{-18}\right|+\left|5.34552942\times 10^{-51}\right|=9.5596$$

Atom-bond connectivity matrix $ABC\left(G_{1}\right)$ of $G_{1}$ is obtained as

$$\left[\begin{array}{cccccccccccccccccccccc} \;0 & \;0.71 & \;0 & \;0 & \;0 & \;0 & \;0 & \;0 & \;0 & \;0 & \;0 & \;0 & \;0 & \;0 & \;0 & \;0 & \;0 & \;0 & \;0 & \;0 & \;0 & \;0\;\\ \;0.71 & \;0 & \;0.65 & \;0 & \;0 & \;0 & \;0 & \;0 & \;0 & \;0 & \;0 & \;0 & \;0.65 & \;0 & \;0 & \;0 & \;0 & \;0 & \;0 & \;0 & \;0 & \;0\\ \;0 & \;0.65 & \;0 & \;0.65 & \;0 & \;0 & \;0 & \;0 & \;0 & \;0 & \;0 & \;0 & \;0 & \;0.82 & \;0 & \;0 & \;0 & \;0 & \;0 & \;0 & \;0 & \;0\\ \;0 & \;0 & \;0.65 & \;0 & \;0.71 & \;0 & \;0 & \;0 & \;0 & \;0 & \;0.61 & \;0 & \;0 & \;0 & \;0 & \;0 & \;0 & \;0 & \;0 & \;0 & \;0 & \;0\\ \;0 & \;0 & \;0 & \;0.71 & \;0 & \;0.71 & \;0 & \;0 & \;0 & \;0 & \;0 & \;0 & \;0 & \;0 & \;0 & \;0 & \;0 & \;0 & \;0 & \;0 & \;0 & \;0\\ \;0 & \;0 & \;0 & \;0 & \;0.71 & \;0 & \;0.65 & \;0 & \;0 & \;0 & \;0 & \;0 & \;0 & \;0 & \;0 & \;0 & \;0.61 & \;0.87 & \;0 & \;0 & \;0 & \;0\\ \;0 & \;0 & \;0 & \;0 & \;0 & \;0.65 & \;0 & \;0.65 & \;0 & \;0 & \;0 & \;0 & \;0 & \;0 & \;0 & \;0 & \;0 & \;0 & \;0 & \;0 & \;0 & \;0\\ \;0 & \;0 & \;0 & \;0 & \;0 & \;0 & \;0.65 & \;0 & \;0.71 & \;0 & \;0 & \;0 & \;0 & \;0 & \;0 & \;0 & \;0 & \;0 & \;0.87 & \;0 & \;0 & \;0\\ \;0 & \;0 & \;0 & \;0 & \;0 & \;0 & \;0 & \;0.71 & \;0 & \;0.71 & \;0 & \;0 & \;0 & \;0 & \;0 & \;0 & \;0 & \;0 & \;0 & \;0 & \;0 & \;0\\ \;0 & \;0 & \;0 & \;0 & \;0 & \;0 & \;0 & \;0 & \;0.71 & \;0 & \;0.61 & \;0 & \;0 & \;0 & \;0 & \;0 & \;0.61 & \;0 & \;0 & \;0.65 & \;0 & \;0\\ \;0 & \;0 & \;0 & \;0.61 & \;0 & \;0 & \;0 & \;0 & \;0 & \;0.61 & \;0 & \;0.65 & \;0 & \;0 & \;0 & \;0 & \;0 & \;0 & \;0 & \;0 & \;0 & \;0\\ \;0 & \;0 & \;0 & \;0 & \;0 & \;0 & \;0 & \;0 & \;0 & \;0 & \;0.65 & \;0 & \;0.67 & \;0 & \;0 & \;0 & \;0 & \;0 & \;0 & \;0 & \;0 & \;0\\ \;0 & \;0.65 & \;0 & \;0 & \;0 & \;0 & \;0 & \;0 & \;0 & \;0 & \;0 & \;0.67 & \;0 & \;0 & \;0 & \;0 & \;0 & \;0 & \;0 & \;0 & \;0 & \;0\\ \;0 & \;0 & \;0.82 & \;0 & \;0 & \;0 & \;0 & \;0 & \;0 & \;0 & \;0 & \;0 & \;0 & \;0 & \;0 & \;0 & \;0 & \;0 & \;0 & \;0 & \;0 & \;0\\ \;0 & \;0 & \;0 & \;0 & \;0 & \;0 & \;0 & \;0 & \;0 & \;0 & \;0 & \;0 & \;0 & \;0 & \;0 & \;0.82 & \;0 & \;0 & \;0 & \;0 & \;0 & \;0\\ \;0 & \;0 & \;0 & \;0 & \;0 & \;0 & \;0 & \;0 & \;0 & \;0 & \;0 & \;0 & \;0 & \;0 & \;0.82 & \;0 & \;0.65 & \;0 & \;0 & \;0 & \;0 & \;0\\ \;0 & \;0 & \;0 & \;0 & \;0 & \;0.61 & \;0 & \;0 & \;0 & \;0.61 & \;0 & \;0 & \;0 & \;0 & \;0 & \;0.65 & \;0 & \;0 & \;0 & \;0 & \;0 & \;0\\ \;0 & \;0 & \;0 & \;0 & \;0 & \;0.87 & \;0 & \;0 & \;0 & \;0 & \;0 & \;0 & \;0 & \;0 & \;0 & \;0 & \;0 & \;0 & \;0 & \;0 & \;0 & \;0\\ \;0 & \;0 & \;0 & \;0 & \;0 & \;0 & \;0 & \;0.87 & \;0 & \;0 & \;0 & \;0 & \;0 & \;0 & \;0 & \;0 & \;0 & \;0 & \;0 & \;0 & \;0 & \;0\\ \;0 & \;0 & \;0 & \;0 & \;0 & \;0 & \;0 & \;0 & \;0 & \;0.65 & \;0 & \;0 & \;0 & \;0 & \;0 & \;0 & \;0 & \;0 & \;0 & \;0 & \;0.82 & \;0.82\\ \;0 & \;0 & \;0 & \;0 & \;0 & \;0 & \;0 & \;0 & \;0 & \;0 & \;0 & \;0 & \;0 & \;0 & \;0 & \;0 & \;0 & \;0 & \;0 & \;0.82 & \;0 & \;0\\ \;0 & \;0 & \;0 & \;0 & \;0 & \;0 & \;0 & \;0 & \;0 & \;0 & \;0 & \;0 & \;0 & \;0 & \;0 & \;0 & \;0 & \;0 & \;0 & \;0.82 & \;0 & \;0 \end{array}\right]$$

The characteristic equation of $ABC\left(G_{1}\right)$ is $\left|ABC\left(G_{1}\right)-\lambda I\right|=0$ , where I is the unit matrix of order 22. That is,

$$\lambda^{22}+\left(1.77479446\times 10^{-15}\right)\lambda^{21}-11.9444444\lambda^{20}-\left(4.42223034\times 10^{-15}\right)\lambda^{19}+59.6313657\lambda^{18}+\left(2.30833222\times 10^{-13}\right)\lambda^{17}-162.613879\lambda^{16}+\left(5.18950262\times 10^{-13}\right)\lambda^{15}+265.210284\lambda^{14}+\left(1.54856880\times 10^{-12}\right)\lambda^{13}-265.730702\lambda^{12}+\left(8.95699019\times 10^{-13}\right)\lambda^{11}+161.219498\lambda^{10}+\left(4.68706320\times 10^{-13}\right)\lambda^{9}-55.7862717\lambda^{8}+\left(7.82468603\times 10^{-14}\right)\lambda^{7}+9.61063123\lambda^{6}+\left(1.53929678\times 10^{-15}\right)\lambda^{5}-0.597149884\lambda^{4}+\left(9.32974659\times 10^{-19}\right)\lambda^{3}-\left(1.03064934\times 10^{-36}\right)\lambda^{2}-\left(1.02537607\times 10^{-70}\right)\lambda +0=0$$

The eigenvalues ${\left\{\lambda_{i_{ABC}}\right\}}_{i=1}^{22}$ of ABC are −1.77925631, 1.77925631, −1.47585081, −1.39622420, −1.22175204, −1.02184588, −0.955125701, −0.871067683, −0.557427565, −0.364029180, 1.47585081, 1.39622420, 1.22175204, 1.02184588, 0.955125701, 0.871067683, 0.557427565, 0.364029180, 7.81189684 × 10^−19^, 7.81189684 × 10^−19^, −9.94883540 × 10^−35^ and 0.

Thus, the Atom-bond connectivity energy $E_{ABC}\left(G_{1}\right)$ is

$$E_{ABC}\left(G_{1}\right)=\sum_{i=1}^{\left|V\right|}\left|\lambda_{i_{ABC}}\right|=\sum_{i=1}^{22}\left|\lambda_{i_{ABC}}\right|$$

$$=|-1.77925631|+|1.77925631|+|-1.47585081|+|-1.39622420|+|-1.22175204|+|-1.02184588|+|-0.955125701|+|-0.871067683|+|-0.557427565|+|-0.364029180|+|1.47585081|+|1.39622420|+|1.22175204|+|1.02184588|+|0.955125701|+|0.871067683|+|0.557427565|+|0.364029180|+|7.81189684\times 10^{-19}|+|7.81189684\times 10^{-19}|+|-9.94883540\times 10^{-35}|+|0|=19.2852$$

Geometric-Arithmetic matrix $GA\left(G_{1}\right)$ is obtained as

$$\left[\begin{array}{cccccccccccccccccccccc} \;0 & \;0.94 & \;0 & \;0 & \;0 & \;0 & \;0 & \;0 & \;0 & \;0 & \;0 & \;0 & \;0 & \;0 & \;0 & \;0 & \;0 & \;0 & \;0 & \;0 & \;0 & \;0\;\\ \;0.94 & \;0 & \;0.99 & \;0 & \;0 & \;0 & \;0 & \;0 & \;0 & \;0 & \;0 & \;0 & \;0.99 & \;0 & \;0 & \;0 & \;0 & \;0 & \;0 & \;0 & \;0 & \;0\\ \;0 & \;0.99 & \;0 & \;0.99 & \;0 & \;0 & \;0 & \;0 & \;0 & \;0 & \;0 & \;0 & \;0 & \;0.87 & \;0 & \;0 & \;0 & \;0 & \;0 & \;0 & \;0 & \;0\\ \;0 & \;0 & \;0.99 & \;0 & \;0.94 & \;0 & \;0 & \;0 & \;0 & \;0 & \;1 & \;0 & \;0 & \;0 & \;0 & \;0 & \;0 & \;0 & \;0 & \;0 & \;0 & \;0\\ \;0 & \;0 & \;0 & \;0.94 & \;0 & \;0.94 & \;0 & \;0 & \;0 & \;0 & \;0 & \;0 & \;0 & \;0 & \;0 & \;0 & \;0 & \;0 & \;0 & \;0 & \;0 & \;0\\ \;0 & \;0 & \;0 & \;0 & \;0.94 & \;0 & \;0.98 & \;0 & \;0 & \;0 & \;0 & \;0 & \;0 & \;0 & \;0 & \;0 & \;1 & \;0.80 & \;0 & \;0 & \;0 & \;0\\ \;0 & \;0 & \;0 & \;0 & \;0 & \;0.99 & \;0 & \;0.99 & \;0 & \;0 & \;0 & \;0 & \;0 & \;0 & \;0 & \;0 & \;0 & \;0 & \;0 & \;0 & \;0 & \;0\\ \;0 & \;0 & \;0 & \;0 & \;0 & \;0 & \;0.99 & \;0 & \;0.94 & \;0 & \;0 & \;0 & \;0 & \;0 & \;0 & \;0 & \;0 & \;0 & \;0.80 & \;0 & \;0 & \;0\\ \;0 & \;0 & \;0 & \;0 & \;0 & \;0 & \;0 & \;0.94 & \;0 & \;0.94 & \;0 & \;0 & \;0 & \;0 & \;0 & \;0 & \;0 & \;0 & \;0 & \;0 & \;0 & \;0\\ \;0 & \;0 & \;0 & \;0 & \;0 & \;0 & \;0 & \;0 & \;0.94 & \;0 & \;1 & \;0 & \;0 & \;0 & \;0 & \;0 & \;1 & \;0 & \;0 & \;0.99 & \;0 & \;0\\ \;0 & \;0 & \;0 & \;1 & \;0 & \;0 & \;0 & \;0 & \;0 & \;1 & \;0 & \;0.99 & \;0 & \;0 & \;0 & \;0 & \;0 & \;0 & \;0 & \;0 & \;0 & \;0\\ \;0 & \;0 & \;0 & \;0 & \;0 & \;0 & \;0 & \;0 & \;0 & \;0 & \;0.99 & \;0 & \;1 & \;0 & \;0 & \;0 & \;0 & \;0 & \;0 & \;0 & \;0 & \;0\\ \;0 & \;0.99 & \;0 & \;0 & \;0 & \;0 & \;0 & \;0 & \;0 & \;0 & \;0 & \;1 & \;0 & \;0 & \;0 & \;0 & \;0 & \;0 & \;0 & \;0 & \;0 & \;0\\ \;0 & \;0 & \;0.87 & \;0 & \;0 & \;0 & \;0 & \;0 & \;0 & \;0 & \;0 & \;0 & \;0 & \;0 & \;0 & \;0 & \;0 & \;0 & \;0 & \;0 & \;0 & \;0\\ \;0 & \;0 & \;0 & \;0 & \;0 & \;0 & \;0 & \;0 & \;0 & \;0 & \;0 & \;0 & \;0 & \;0 & \;0 & \;0.87 & \;0 & \;0 & \;0 & \;0 & \;0 & \;0\\ \;0 & \;0 & \;0 & \;0 & \;0 & \;0 & \;0 & \;0 & \;0 & \;0 & \;0 & \;0 & \;0 & \;0 & \;0.87 & \;0 & \;0.99 & \;0 & \;0 & \;0 & \;0 & \;0\\ \;0 & \;0 & \;0 & \;0 & \;0 & \;1 & \;0 & \;0 & \;0 & \;1 & \;0 & \;0 & \;0 & \;0 & \;0 & \;0.99 & \;0 & \;0 & \;0 & \;0 & \;0 & \;0\\ \;0 & \;0 & \;0 & \;0 & \;0 & \;0.80 & \;0 & \;0 & \;0 & \;0 & \;0 & \;0 & \;0 & \;0 & \;0 & \;0 & \;0 & \;0 & \;0 & \;0 & \;0 & \;0\\ \;0 & \;0 & \;0 & \;0 & \;0 & \;0 & \;0 & \;0.80 & \;0 & \;0 & \;0 & \;0 & \;0 & \;0 & \;0 & \;0 & \;0 & \;0 & \;0 & \;0 & \;0 & \;0\\ \;0 & \;0 & \;0 & \;0 & \;0 & \;0 & \;0 & \;0 & \;0 & \;0.99 & \;0 & \;0 & \;0 & \;0 & \;0 & \;0 & \;0 & \;0 & \;0 & \;0 & \;0.87 & \;0.87\\ \;0 & \;0 & \;0 & \;0 & \;0 & \;0 & \;0 & \;0 & \;0 & \;0 & \;0 & \;0 & \;0 & \;0 & \;0 & \;0 & \;0 & \;0 & \;0 & \;0.87 & \;0 & \;0\\ \;0 & \;0 & \;0 & \;0 & \;0 & \;0 & \;0 & \;0 & \;0 & \;0 & \;0 & \;0 & \;0 & \;0 & \;0 & \;0 & \;0 & \;0 & \;0 & \;0.87 & \;0 & \;0 \end{array}\right]$$

The characteristic equation of $GA\left(G_{1}\right)$ is $\left|GA\left(G_{1}\right)-\lambda I\right|=0$ , where I is the unit matrix of order 22. That is,

$$\lambda^{22}+\left(2.16384308\times 10^{-14}\right)\lambda^{21}-21.5611791\lambda^{20}-\left(3.04718497\times 10^{-13}\right)\lambda^{19}+189.291278\lambda^{18}+\left(2.19691269\times 10^{-12}\right)\lambda^{17}-885.549178\lambda^{16}-\left(6.12173511\times 10^{-12}\right)\lambda^{15}+2418.37056\lambda^{14}+\left(1.01122841\times 10^{-11}\right)\lambda^{13}-3954.31543\lambda^{12}-\left(1.29092606\times 10^{-11}\right)\lambda^{11}+3794.18876\lambda^{10}+\left(8.35464655\times 10^{-12}\right)\lambda^{9}-1985.06930\lambda^{8}-\left(2.36225694\times 10^{-12}\right)\lambda^{7}+480.147546\lambda^{6}+\left(2.34574038\times 10^{-13}\right)\lambda^{5}-38.0372159\lambda^{4}-\left(1.69619336\times 10^{-15}\right)\lambda^{3}+\left(1.11977994\times 10^{-31}\right)\lambda^{2}-\left(6.90117667\times 10^{-64}\right)\lambda +0=0$$

The eigenvalues ${\left\{\lambda_{i_{GA}}\right\}}_{i=1}^{22}$ of $GA\left(G_{1}\right)$ are −2.57896966, 2.57896966, −2.09810363, 2.09810363, −1.79736577, −1.50651896, −1.35457707, −1.23112119, −1.06060488, 1.79736577, −0.620997295, −0.383241265, 1.50651896, 1.35457707, 1.23112119, 1.06060488, 0.620997295, 0.383241265, 6.16297582 × 10^−33^, −8.09568750 × 10^−17^, 3.63638806 × 10^−17^ and 0.

The corresponding Geometric-Arithmetic energy $E_{GA}\left(G_{1}\right)$ is

$$E_{GA}\left(G_{1}\right)=\sum_{i=1}^{\left|V\right|}\left|\lambda_{i_{GA}}\right|=\sum_{i=1}^{22}\left|\lambda_{i_{GA}}\right|$$

$$=|-2.57896966|+|2.57896966|+|-2.09810363|+|2.09810363|+|-1.79736577|+|-1.50651896|+|-1.35457707|+|-1.23112119|+|-1.06060488|+|1.79736577|+|-0.620997295|+|-0.383241265|+|1.50651896|+|1.35457707|+|1.23112119|+|1.06060488|+|0.620997295|+|0.383241265|+|6.16297582\times 10^{-33}|+|-8.09568750\times 10^{-17}|+|3.63638806\times 10^{-17}|+|0|=25.2630$$

Hence the proof.

Theorem 2

Let $G_{1}\left(V,E\right)$ be the isomorphic molecular graph of the compound Huperzine A with the atom set V and bond set E . Then the Roman domination number and the Roman energies of $G_{1}$ are given by $\gamma_{R}\left(G_{1}\right)=13,E^{R}\left(G_{1}\right)=45.1296,E_{L}^{R}\left(G_{1}\right)=73.3636,E_{R}^{R}\left(G_{1}\right)=17.9872,E_{H}^{R}\left(G_{1}\right)=17.4217,E_{ABC}^{R}\left(G_{1}\right)=24.6301$ and $E_{GA}^{R}\left(G_{1}\right)=29.7845$ .

Proof:

Consider the isomorphic molecular graph $G_{1}\left(V,E\right)$ of the compound Huperzine A, where the atoms are labelled $v_{1},v_{2},\ldots ,v_{22}$ as depicted in Figure 3b. In the same figure, the atoms are colored red, blue and black to indicate Roman domination function (RDF) values of 2, 1 and 0 respectively.

Since the maximum degree in $G_{1}$ is 4, an RDF value of 2 is first assigned to the atoms with degree 4. Assigning $f\left(v_{2}\right)=2$ dominates $v_{1},v_{3}$ and $v_{13}$ . Furthermore, assigning $f\left(v_{6}\right)=f\left(v_{8}\right)=f\left(v_{11}\right)=f\left(v_{16}\right)=f\left(v_{20}\right)=2$ ensures domination of all the remaining undominated vertices except $v_{14}$ , which is assigned $f\left(v_{14}\right)=1$ . Hence, the vertices in $V\backslash \left\{v_{2},v_{6},v_{8},v_{11},v_{16},v_{20}\right\}$ receive the RDF value 0.

Thus, the weight of f is

$$\sum_{v\in V}f\left(v\right)=\left(6\times 2\right)+1=13$$

Therefore, $\gamma_{R}\left(G_{1}\right)=13$ .

Now, the Roman domination-based adjacency matrix $A^{R}\left(G_{1}\right)$ is obtained as

$$\left[\begin{array}{cccccccccccccccccccccc} \;0 & 3 & 0 & 0 & 0 & 0 & 0 & 0 & 0 & 0 & 0 & 0 & 0 & 0 & 0 & 0 & 0 & 0 & 0 & 0 & 0 & 0\;\\ \;2 & 2 & 1 & 0 & 0 & 0 & 0 & 0 & 0 & 0 & 0 & 0 & 1 & 0 & 0 & 0 & 0 & 0 & 0 & 0 & 0 & 0\;\\ \;0 & 2 & 0 & 1 & 0 & 0 & 0 & 0 & 0 & 0 & 0 & 0 & 0 & 1 & 0 & 0 & 0 & 0 & 0 & 0 & 0 & 0\;\\ \;0 & 0 & 1 & 0 & 1 & 0 & 0 & 0 & 0 & 0 & 3 & 0 & 0 & 0 & 0 & 0 & 0 & 0 & 0 & 0 & 0 & 0\;\\ \;0 & 0 & 0 & 1 & 0 & 2 & 0 & 0 & 0 & 0 & 0 & 0 & 0 & 0 & 0 & 0 & 0 & 0 & 0 & 0 & 0 & 0\;\\ \;0 & 0 & 0 & 0 & 1 & 2 & 1 & 0 & 0 & 0 & 0 & 0 & 0 & 0 & 0 & 0 & 1 & 1 & 0 & 0 & 0 & 0\;\\ \;0 & 0 & 0 & 0 & 0 & 2 & 0 & 3 & 0 & 0 & 0 & 0 & 0 & 0 & 0 & 0 & 0 & 0 & 0 & 0 & 0 & 0\;\\ \;0 & 0 & 0 & 0 & 0 & 0 & 2 & 2 & 1 & 0 & 0 & 0 & 0 & 0 & 0 & 0 & 0 & 0 & 1 & 0 & 0 & 0\;\\ \;0 & 0 & 0 & 0 & 0 & 0 & 0 & 2 & 0 & 1 & 0 & 0 & 0 & 0 & 0 & 0 & 0 & 0 & 0 & 0 & 0 & 0\;\\ \;0 & 0 & 0 & 0 & 0 & 0 & 0 & 0 & 1 & 0 & 2 & 0 & 0 & 0 & 0 & 0 & 1 & 0 & 0 & 2 & 0 & 0\;\\ \;0 & 0 & 0 & 2 & 0 & 0 & 0 & 0 & 0 & 1 & 2 & 1 & 0 & 0 & 0 & 0 & 0 & 0 & 0 & 0 & 0 & 0\;\\ \;0 & 0 & 0 & 0 & 0 & 0 & 0 & 0 & 0 & 0 & 2 & 0 & 2 & 0 & 0 & 0 & 0 & 0 & 0 & 0 & 0 & 0\;\\ \;0 & 2 & 0 & 0 & 0 & 0 & 0 & 0 & 0 & 0 & 0 & 2 & 0 & 0 & 0 & 0 & 0 & 0 & 0 & 0 & 0 & 0\;\\ \;0 & 0 & 1 & 0 & 0 & 0 & 0 & 0 & 0 & 0 & 0 & 0 & 0 & 1 & 0 & 0 & 0 & 0 & 0 & 0 & 0 & 0\;\\ \;0 & 0 & 0 & 0 & 0 & 0 & 0 & 0 & 0 & 0 & 0 & 0 & 0 & 0 & 0 & 2 & 0 & 0 & 0 & 0 & 0 & 0\;\\ \;0 & 0 & 0 & 0 & 0 & 0 & 0 & 0 & 0 & 0 & 0 & 0 & 0 & 0 & 1 & 2 & 2 & 0 & 0 & 0 & 0 & 0\;\\ \;0 & 0 & 0 & 0 & 0 & 2 & 0 & 0 & 0 & 1 & 0 & 0 & 0 & 0 & 0 & 3 & 0 & 0 & 0 & 0 & 0 & 0\;\\ \;0 & 0 & 0 & 0 & 0 & 2 & 0 & 0 & 0 & 0 & 0 & 0 & 0 & 0 & 0 & 0 & 0 & 0 & 0 & 0 & 0 & 0\;\\ \;0 & 0 & 0 & 0 & 0 & 0 & 0 & 2 & 0 & 0 & 0 & 0 & 0 & 0 & 0 & 0 & 0 & 0 & 0 & 0 & 0 & 0\;\\ \;0 & 0 & 0 & 0 & 0 & 0 & 0 & 0 & 0 & 1 & 0 & 0 & 0 & 0 & 0 & 0 & 0 & 0 & 0 & 2 & 1 & 1\;\\ \;0 & 0 & 0 & 0 & 0 & 0 & 0 & 0 & 0 & 0 & 0 & 0 & 0 & 0 & 0 & 0 & 0 & 0 & 0 & 2 & 0 & 0\;\\ \;0 & 0 & 0 & 0 & 0 & 0 & 0 & 0 & 0 & 0 & 0 & 0 & 0 & 0 & 0 & 0 & 0 & 0 & 0 & 2 & 0 & 0\; \end{array}\right]$$

The characteristic equation of $A^{R}\left(G_{1}\right)$ is $\left|A^{R}\left(G_{1}\right)-\lambda I\right|=0$ , where I is the unit matrix of order 22. That is,

$$\lambda^{22}-13\lambda^{21}+11\lambda^{20}+468\lambda^{19}-1301\lambda^{18}-6970\lambda^{17}+24906\lambda^{16}+58412\lambda^{15}-223070\lambda^{14}-320264\lambda^{13}+1068982\lambda^{12}+1201236\lambda^{11}-2692488\lambda^{10}-2775208\lambda^{9}+3159032\lambda^{8}+3216480\lambda^{7}-1222368\lambda^{6}-1352384\lambda^{5}-61440\lambda^{4}+52992\lambda^{3}+\left(1.02430114\times 10^{-11}\right)\lambda^{2}+\left(3.78958860\times 10^{-28}\right)\lambda -\left(2.91939286\times 10^{-61}\right)=0$$

Now, the eigenvalues ${\left\{\lambda_{i}^{R}\right\}}_{i=1}^{22}$ of $A^{R}\left(G_{1}\right)$ are 5.00197952, 4.65503255, 4.24336745, 4.11235793, 3.54682450, 3.12725603, −3.33264201, 1.92083543, 1.38241044, −2.89119702, 0.903825166, −2.27140484, −2.02129249, −1.64560765, −1.85525292, −1.10685842, −0.641833443, −0.298729731, 0.170929512, −4.98562614 × 10^−17^, −1.43437281 × 10^−16^ and 7.70371978 × 10^−34^​.

Hence, the Roman energy $E^{R}\left(G_{1}\right)$ is

$$E^{R}\left(G_{1}\right)=\sum_{i=1}^{\left|V\right|}\left|\lambda_{i}^{R}\right|=\sum_{i=1}^{22}\left|\lambda_{i}^{R}\right|$$

=∣5.00197952∣+∣4.65503255∣+∣4.24336745∣+∣4.11235793∣+∣3.54682450∣+∣3.12725603∣+∣−3.33264201∣+∣1.92083543∣+∣1.38241044∣+∣−2.89119702∣+∣0.903825166∣+∣−2.27140484∣+∣−2.02129249∣+∣−1.64560765∣+∣−1.85525292∣+∣−1.10685842∣+∣−0.641833443∣+∣−0.298729731∣+∣0.170929512∣+∣−4.98562614×10−17∣+∣−1.43437281×10−16∣+∣7.70371978×10−34∣=45.1296

Similarly, the Roman Laplacian matrix is obtained as

$$\left[\begin{array}{cccccccccccccccccccccc} \;3 & -3 & \;0 & \;0 & \;0 & \;0 & \;0 & \;0 & \;0 & \;0 & \;0 & \;0 & \;0 & \;0 & \;0 & \;0 & \;0 & \;0 & \;0 & \;0 & \;0 & \;0\;\\ -2 & \;4 & -1 & \;0 & \;0 & \;0 & \;0 & \;0 & \;0 & \;0 & \;0 & \;0 & -1 & \;0 & \;0 & \;0 & \;0 & \;0 & \;0 & \;0 & \;0 & \;0\;\\ \;0 & -2 & \;4 & -1 & \;0 & \;0 & \;0 & \;0 & \;0 & \;0 & \;0 & \;0 & \;0 & -1 & \;0 & \;0 & \;0 & \;0 & \;0 & \;0 & \;0 & \;0\;\\ \;0 & \;0 & -1 & \;5 & -1 & \;0 & \;0 & \;0 & \;0 & \;0 & -3 & \;0 & \;0 & \;0 & \;0 & \;0 & \;0 & \;0 & \;0 & \;0 & \;0 & \;0\;\\ \;0 & \;0 & \;0 & -1 & \;3 & -2 & \;0 & \;0 & \;0 & \;0 & \;0 & \;0 & \;0 & \;0 & \;0 & \;0 & \;0 & \;0 & \;0 & \;0 & \;0 & \;0\;\\ \;0 & \;0 & \;0 & \;0 & -1 & \;4 & -1 & \;0 & \;0 & \;0 & \;0 & \;0 & \;0 & \;0 & \;0 & \;0 & -1 & -1 & \;0 & \;0 & \;0 & \;0\;\\ \;0 & \;0 & \;0 & \;0 & \;0 & -2 & \;5 & -3 & \;0 & \;0 & \;0 & \;0 & \;0 & \;0 & \;0 & \;0 & \;0 & \;0 & \;0 & \;0 & \;0 & \;0\;\\ \;0 & \;0 & \;0 & \;0 & \;0 & \;0 & -2 & \;4 & -1 & \;0 & \;0 & \;0 & \;0 & \;0 & \;0 & \;0 & \;0 & \;0 & -1 & \;0 & \;0 & \;0\;\\ \;0 & \;0 & \;0 & \;0 & \;0 & \;0 & \;0 & -2 & \;3 & -1 & \;0 & \;0 & \;0 & \;0 & \;0 & \;0 & \;0 & \;0 & \;0 & \;0 & \;0 & \;0\;\\ \;0 & \;0 & \;0 & \;0 & \;0 & \;0 & \;0 & \;0 & -1 & \;6 & -2 & \;0 & \;0 & \;0 & \;0 & \;0 & -1 & \;0 & \;0 & -2 & \;0 & \;0\;\\ \;0 & \;0 & \;0 & -2 & \;0 & \;0 & \;0 & \;0 & \;0 & -1 & \;4 & -1 & \;0 & \;0 & \;0 & \;0 & \;0 & \;0 & \;0 & \;0 & \;0 & \;0\;\\ \;0 & \;0 & \;0 & \;0 & \;0 & \;0 & \;0 & \;0 & \;0 & \;0 & -2 & \;4 & -2 & \;0 & \;0 & \;0 & \;0 & \;0 & \;0 & \;0 & \;0 & \;0\;\\ \;0 & -2 & \;0 & \;0 & \;0 & \;0 & \;0 & \;0 & \;0 & \;0 & \;0 & -2 & \;4 & \;0 & \;0 & \;0 & \;0 & \;0 & \;0 & \;0 & \;0 & \;0\;\\ \;0 & \;0 & -1 & \;0 & \;0 & \;0 & \;0 & \;0 & \;0 & \;0 & \;0 & \;0 & \;0 & \;1 & \;0 & \;0 & \;0 & \;0 & \;0 & \;0 & \;0 & \;0\;\\ \;0 & \;0 & \;0 & \;0 & \;0 & \;0 & \;0 & \;0 & \;0 & \;0 & \;0 & \;0 & \;0 & \;0 & \;2 & -2 & \;0 & \;0 & \;0 & \;0 & \;0 & \;0\;\\ \;0 & \;0 & \;0 & \;0 & \;0 & \;0 & \;0 & \;0 & \;0 & \;0 & \;0 & \;0 & \;0 & \;0 & -1 & \;3 & -2 & \;0 & \;0 & \;0 & \;0 & \;0\;\\ \;0 & \;0 & \;0 & \;0 & \;0 & -2 & \;0 & \;0 & \;0 & -1 & \;0 & \;0 & \;0 & \;0 & \;0 & -3 & \;6 & \;0 & \;0 & \;0 & \;0 & \;0\;\\ \;0 & \;0 & \;0 & \;0 & \;0 & -2 & \;0 & \;0 & \;0 & \;0 & \;0 & \;0 & \;0 & \;0 & \;0 & \;0 & \;0 & \;2 & \;0 & \;0 & \;0 & \;0\;\\ \;0 & \;0 & \;0 & \;0 & \;0 & \;0 & \;0 & -2 & \;0 & \;0 & \;0 & \;0 & \;0 & \;0 & \;0 & \;0 & \;0 & \;0 & \;2 & \;0 & \;0 & \;0\;\\ \;0 & \;0 & \;0 & \;0 & \;0 & \;0 & \;0 & \;0 & \;0 & -1 & \;0 & \;0 & \;0 & \;0 & \;0 & \;0 & \;0 & \;0 & \;0 & \;3 & -1 & -1\\ \;0 & \;0 & \;0 & \;0 & \;0 & \;0 & \;0 & \;0 & \;0 & \;0 & \;0 & \;0 & \;0 & \;0 & \;0 & \;0 & \;0 & \;0 & \;0 & -2 & \;2 & \;0\;\\ \;0 & \;0 & \;0 & \;0 & \;0 & \;0 & \;0 & \;0 & \;0 & \;0 & \;0 & \;0 & \;0 & \;0 & \;0 & \;0 & \;0 & \;0 & \;0 & -2 & \;0 & \;2\; \end{array}\right]$$

The characteristic equation of $L^{R}\left(G_{1}\right)$ is $\left|L^{R}\left(G_{1}\right)-\lambda I\right|=0$ , where I is the unit matrix of order 22. That is,

$$\lambda^{22}-76\lambda^{21}+2677\lambda^{20}-58047\lambda^{19}+868033\lambda^{18}-9504611\lambda^{17}+78971155\lambda^{16}-509182024\lambda^{15}+2583936620\lambda^{14}-10406895500\lambda^{13}+33395563500\lambda^{12}-85397809400\lambda^{11}+\left(1.73431896\times 10^{11}\right)\lambda^{10}-\left(2.77780466\times 10^{11}\right)\lambda^{9}+\left(3.47037333\times 10^{11}\right)\lambda^{8}-\left(3.32837945\times 10^{11}\right)\lambda^{7}+\left(2.39635339\times 10^{11}\right)\lambda^{6}-\left(1.25488638\times 10^{11}\right)\lambda^{5}+\left(4.56410733\times 10^{10}\right)\lambda^{4}-\left(1.07277581\times 10^{10}\right)\lambda^{3}+\left(1.43408819\times 10^{9}\right)\lambda^{2}-\left(8.08007680\times 10^{7}\right)\lambda -\left(1.34358995\times 10^{-7}\right)=0$$

The eigenvalues ${\left\{\lambda_{i_{L}}^{R}\right\}}_{i=1}^{22}$ of $L^{R}\left(G_{1}\right)$ are 8.71188669, 7.90287273, 7.46619932, 7.00646362, 6.47066545, 5.78247993, 5.08008995, −1.66284304 × 10^−15^, 0.179634985, 0.312968381, 0.526054399, 0.816748345, 0.929690725, 1.16174888, 4.17886192, 3.82914814, 3.49859993, 2.90766712, 2.69306926, 2.20458601, 2.34056421 and 2.00000000.

Hence, the Roman Laplacian energy $E_{L}^{R}\left(G_{1}\right)$ is

$$E_{L}^{R}\left(G_{1}\right)=\left|\sum_{i=1}^{\left|V\right|}\lambda_{i_{L}}^{R}-\frac{2\left|E\right|}{\left|V\right|}\right|=\left|\sum_{i=1}^{22}\lambda_{i_{L}}^{R}-\frac{2\left(29\right)}{22}\right|$$

$$=\left|\left[8.71188669+7.90287273+7.46619932+7.00646362+6.47066545+5.78247993+5.08008995-1.66284304\times 10^{-15}+0.179634985+0.312968381+0.526054399+0.816748345+0.929690725+1.16174888+4.17886192+3.82914814+3.49859993+2.90766712+2.69306926+2.20458601+2.34056421+2.00000000\right]-\left(\frac{2\left(29\right)}{22}\right)\right|=\left|76-\left(\frac{58}{22}\right)\right|=73.3636$$

The Roman Randić matrix $R^{R}\left(G_{1}\right)$ is obtained as

$$\left[\begin{array}{cccccccccccccccccccccc} \;0 & \;0.35 & \;0 & \;0 & \;0 & \;0 & \;0 & \;0 & \;0 & \;0 & \;0 & \;0 & \;0 & \;0 & \;0 & \;0 & \;0 & \;0 & \;0 & \;0 & \;0 & \;0\;\\ \;0.35 & \;2 & \;0.29 & \;0 & \;0 & \;0 & \;0 & \;0 & \;0 & \;0 & \;0 & \;0 & \;0.29 & \;0 & \;0 & \;0 & \;0 & \;0 & \;0 & \;0 & \;0 & \;0\\ \;0 & \;0.29 & \;0 & \;0.29 & \;0 & \;0 & \;0 & \;0 & \;0 & \;0 & \;0 & \;0 & \;0 & \;0.58 & \;0 & \;0 & \;0 & \;0 & \;0 & \;0 & \;0 & \;0\\ \;0 & \;0 & \;0.29 & \;0 & \;0.35 & \;0 & \;0 & \;0 & \;0 & \;0 & \;0.25 & \;0 & \;0 & \;0 & \;0 & \;0 & \;0 & \;0 & \;0 & \;0 & \;0 & \;0\\ \;0 & \;0 & \;0 & \;0.35 & \;0 & \;0.35 & \;0 & \;0 & \;0 & \;0 & \;0 & \;0 & \;0 & \;0 & \;0 & \;0 & \;0 & \;0 & \;0 & \;0 & \;0 & \;0\\ \;0 & \;0 & \;0 & \;0 & \;0.35 & \;2 & \;0.29 & \;0 & \;0 & \;0 & \;0 & \;0 & \;0 & \;0 & \;0 & \;0 & \;0.25 & \;0.50 & \;0 & \;0 & \;0 & \;0\\ \;0 & \;0 & \;0 & \;0 & \;0 & \;0.29 & \;0 & \;0.29 & \;0 & \;0 & \;0 & \;0 & \;0 & \;0 & \;0 & \;0 & \;0 & \;0 & \;0 & \;0 & \;0 & \;0\\ \;0 & \;0 & \;0 & \;0 & \;0 & \;0 & \;0.29 & \;2 & \;0.35 & \;0 & \;0 & \;0 & \;0 & \;0 & \;0 & \;0 & \;0 & \;0 & \;0.50 & \;0 & \;0 & \;0\\ \;0 & \;0 & \;0 & \;0 & \;0 & \;0 & \;0 & \;0.35 & \;0 & \;0.35 & \;0 & \;0 & \;0 & \;0 & \;0 & \;0 & \;0 & \;0 & \;0 & \;0 & \;0 & \;0\\ \;0 & \;0 & \;0 & \;0 & \;0 & \;0 & \;0 & \;0 & \;0.35 & \;0 & \;0.25 & \;0 & \;0 & \;0 & \;0 & \;0 & \;0.25 & \;0 & \;0 & \;0.29 & \;0 & \;0\\ \;0 & \;0 & \;0 & \;0.25 & \;0 & \;0 & \;0 & \;0 & \;0 & \;0.25 & \;2 & \;0.29 & \;0 & \;0 & \;0 & \;0 & \;0 & \;0 & \;0 & \;0 & \;0 & \;0\\ \;0 & \;0 & \;0 & \;0 & \;0 & \;0 & \;0 & \;0 & \;0 & \;0 & \;0.29 & \;0 & \;0.33 & \;0 & \;0 & \;0 & \;0 & \;0 & \;0 & \;0 & \;0 & \;0\\ \;0 & \;0.29 & \;0 & \;0 & \;0 & \;0 & \;0 & \;0 & \;0 & \;0 & \;0 & \;0.33 & \;0 & \;0 & \;0 & \;0 & \;0 & \;0 & \;0 & \;0 & \;0 & \;0\\ \;0 & \;0 & \;0.58 & \;0 & \;0 & \;0 & \;0 & \;0 & \;0 & \;0 & \;0 & \;0 & \;0 & \;1 & \;0 & \;0 & \;0 & \;0 & \;0 & \;0 & \;0 & \;0\\ \;0 & \;0 & \;0 & \;0 & \;0 & \;0 & \;0 & \;0 & \;0 & \;0 & \;0 & \;0 & \;0 & \;0 & \;0 & \;0.58 & \;0 & \;0 & \;0 & \;0 & \;0 & \;0\\ \;0 & \;0 & \;0 & \;0 & \;0 & \;0 & \;0 & \;0 & \;0 & \;0 & \;0 & \;0 & \;0 & \;0 & \;0.58 & \;2 & \;0.29 & \;0 & \;0 & \;0 & \;0 & \;0\\ \;0 & \;0 & \;0 & \;0 & \;0 & \;0.25 & \;0 & \;0 & \;0 & \;0.25 & \;0 & \;0 & \;0 & \;0 & \;0 & \;0.29 & \;0 & \;0 & \;0 & \;0 & \;0 & \;0\\ \;0 & \;0 & \;0 & \;0 & \;0 & \;0.50 & \;0 & \;0 & \;0 & \;0 & \;0 & \;0 & \;0 & \;0 & \;0 & \;0 & \;0 & \;0 & \;0 & \;0 & \;0 & \;0\\ \;0 & \;0 & \;0 & \;0 & \;0 & \;0 & \;0 & \;0.50 & \;0 & \;0 & \;0 & \;0 & \;0 & \;0 & \;0 & \;0 & \;0 & \;0 & \;0 & \;0 & \;0 & \;0\\ \;0 & \;0 & \;0 & \;0 & \;0 & \;0 & \;0 & \;0 & \;0 & \;0.29 & \;0 & \;0 & \;0 & \;0 & \;0 & \;0 & \;0 & \;0 & \;0 & \;2 & \;0.58 & \;0.58\\ \;0 & \;0 & \;0 & \;0 & \;0 & \;0 & \;0 & \;0 & \;0 & \;0 & \;0 & \;0 & \;0 & \;0 & \;0 & \;0 & \;0 & \;0 & \;0 & \;0.58 & \;0 & \;0\\ \;0 & \;0 & \;0 & \;0 & \;0 & \;0 & \;0 & \;0 & \;0 & \;0 & \;0 & \;0 & \;0 & \;0 & \;0 & \;0 & \;0 & \;0 & \;0 & \;0.58 & \;0 & \;0 \end{array}\right]$$

The characteristic equation of $R^{R}\left(G_{1}\right)$ is $\left|R^{R}\left(G_{1}\right)-\lambda I\right|=0$ , where I is the unit matrix of order 22. That is,

$$\lambda^{22}-13\lambda^{21}+68.5138889\lambda^{20}-180.305556\lambda^{19}+216.331742\lambda^{18}+0.170283565\lambda^{17}-253.962812\lambda^{16}+134.684046\lambda^{15}+123.374355\lambda^{14}-76.3486117\lambda^{13}-42.6177555\lambda^{12}+13.3593265\lambda^{11}+8.18735049\lambda^{10}-0.44141793\lambda^{9}-0.700788647\lambda^{8}-0.0707425299\lambda^{7}+0.0170778607\lambda^{6}+\left(4.17328927\times 10^{-3}\right)\lambda^{5}+\left(3.11392325\times 10^{-4}\right)\lambda^{4}+\left(7.53520448\times 10^{-6}\right)\lambda^{3}-\left(4.83171544\times 10^{-22}\right)\lambda^{2}-\left(2.76972942\times 10^{-38}\right)\lambda -\left(3.70268946\times 10^{-86}\right)=0$$

The eigenvalues ${\left\{\lambda_{i_{R}}^{R}\right\}}_{i=1}^{22}$ of $R^{R}\left(G_{1}\right)$ are 2.32896489, 2.26621949, 2.09484018, 2.14641802, 2.16685294, 2.19908915, 1.25973871, 0.395646561, 0.359895558, 0.275951838, −0.567070822, −0.509522386, −0.368296016, −0.050779806, −0.285816933, −0.242892081, −0.117375768, −0.156443512, −0.195420025, 1.00643910 × 10^−16^, −3.65220210 × 10^−17^ and −1.33684158 × 10^−48^

Thus, the Roman Randić energy $E_{R}^{R}\left(G_{1}\right)$ is

$$E_{R}^{R}\left(G_{1}\right)=\sum_{i=1}^{\left|V\right|}\left|\lambda_{i_{R}}^{R}\right|=\sum_{i=1}^{22}\left|\lambda_{i_{R}}^{R}\right|$$

$$=|2.32896489|+|2.26621949|+|2.09484018|+|2.14641802|+|2.16685294|+|2.19908915|+|1.25973871|+|0.395646561|+|0.359895558|+|0.275951838|+|-0.567070822|+|-0.509522386|+|-0.368296016|+|-0.050779806|+|-0.285816933|+|-0.242892081|+|-0.117375768|+|-0.156443512|+|-0.195420025|+|1.00643910\times 10^{-16}|+|-3.65220210\times 10^{-17}|+|-1.33684158\times 10^{-48}|=17.9872$$

Similarly, the Roman Harmonic matrix $H^{R}\left(G_{1}\right)$ is obtained as

$$\left[\begin{array}{cccccccccccccccccccccc} \;0 & \;0.35 & \;0 & \;0 & \;0 & \;0 & \;0 & \;0 & \;0 & \;0 & \;0 & \;0 & \;0 & \;0 & \;0 & \;0 & \;0 & \;0 & \;0 & \;0 & \;0 & \;0\;\\ \;0.35 & \;2 & \;0.29 & \;0 & \;0 & \;0 & \;0 & \;0 & \;0 & \;0 & \;0 & \;0 & \;0.29 & \;0 & \;0 & \;0 & \;0 & \;0 & \;0 & \;0 & \;0 & \;0\\ \;0 & \;0.29 & \;0 & \;0.29 & \;0 & \;0 & \;0 & \;0 & \;0 & \;0 & \;0 & \;0 & \;0 & \;0.50 & \;0 & \;0 & \;0 & \;0 & \;0 & \;0 & \;0 & \;0\\ \;0 & \;0 & \;0.29 & \;0 & \;0.33 & \;0 & \;0 & \;0 & \;0 & \;0 & \;0.25 & \;0 & \;0 & \;0 & \;0 & \;0 & \;0 & \;0 & \;0 & \;0 & \;0 & \;0\\ \;0 & \;0 & \;0 & \;0.33 & \;0 & \;0.33 & \;0 & \;0 & \;0 & \;0 & \;0 & \;0 & \;0 & \;0 & \;0 & \;0 & \;0 & \;0 & \;0 & \;0 & \;0 & \;0\\ \;0 & \;0 & \;0 & \;0 & \;0.33 & \;2 & \;0.29 & \;0 & \;0 & \;0 & \;0 & \;0 & \;0 & \;0 & \;0 & \;0 & \;0.25 & \;0.40 & \;0 & \;0 & \;0 & \;0\\ \;0 & \;0 & \;0 & \;0 & \;0 & \;0.29 & \;0 & \;0.29 & \;0 & \;0 & \;0 & \;0 & \;0 & \;0 & \;0 & \;0 & \;0 & \;0 & \;0 & \;0 & \;0 & \;0\\ \;0 & \;0 & \;0 & \;0 & \;0 & \;0 & \;0.29 & \;2 & \;0.33 & \;0 & \;0 & \;0 & \;0 & \;0 & \;0 & \;0 & \;0 & \;0 & \;0.40 & \;0 & \;0 & \;0\\ \;0 & \;0 & \;0 & \;0 & \;0 & \;0 & \;0 & \;0.33 & \;0 & \;0.33 & \;0 & \;0 & \;0 & \;0 & \;0 & \;0 & \;0 & \;0 & \;0 & \;0 & \;0 & \;0\\ \;0 & \;0 & \;0 & \;0 & \;0 & \;0 & \;0 & \;0 & \;0.33 & \;0 & \;0.25 & \;0 & \;0 & \;0 & \;0 & \;0 & \;0.25 & \;0 & \;0 & \;0.29 & \;0 & \;0\\ \;0 & \;0 & \;0 & \;0.25 & \;0 & \;0 & \;0 & \;0 & \;0 & \;0.25 & \;2 & \;0.29 & \;0 & \;0 & \;0 & \;0 & \;0 & \;0 & \;0 & \;0 & \;0 & \;0\\ \;0 & \;0 & \;0 & \;0 & \;0 & \;0 & \;0 & \;0 & \;0 & \;0 & \;0.29 & \;0 & \;0.33 & \;0 & \;0 & \;0 & \;0 & \;0 & \;0 & \;0 & \;0 & \;0\\ \;0 & \;0.29 & \;0 & \;0 & \;0 & \;0 & \;0 & \;0 & \;0 & \;0 & \;0 & \;0.33 & \;0 & \;0 & \;0 & \;0 & \;0 & \;0 & \;0 & \;0 & \;0 & \;0\\ \;0 & \;0 & \;0.50 & \;0 & \;0 & \;0 & \;0 & \;0 & \;0 & \;0 & \;0 & \;0 & \;0 & \;1 & \;0 & \;0 & \;0 & \;0 & \;0 & \;0 & \;0 & \;0\\ \;0 & \;0 & \;0 & \;0 & \;0 & \;0 & \;0 & \;0 & \;0 & \;0 & \;0 & \;0 & \;0 & \;0 & \;0 & \;0.50 & \;0 & \;0 & \;0 & \;0 & \;0 & \;0\\ \;0 & \;0 & \;0 & \;0 & \;0 & \;0 & \;0 & \;0 & \;0 & \;0 & \;0 & \;0 & \;0 & \;0 & \;0.50 & \;2 & \;0.29 & \;0 & \;0 & \;0 & \;0 & \;0\\ \;0 & \;0 & \;0 & \;0 & \;0 & \;0.25 & \;0 & \;0 & \;0 & \;0.25 & \;0 & \;0 & \;0 & \;0 & \;0 & \;0.29 & \;0 & \;0 & \;0 & \;0 & \;0 & \;0\\ \;0 & \;0 & \;0 & \;0 & \;0 & \;0.40 & \;0 & \;0 & \;0 & \;0 & \;0 & \;0 & \;0 & \;0 & \;0 & \;0 & \;0 & \;0 & \;0 & \;0 & \;0 & \;0\\ \;0 & \;0 & \;0 & \;0 & \;0 & \;0 & \;0 & \;0.40 & \;0 & \;0 & \;0 & \;0 & \;0 & \;0 & \;0 & \;0 & \;0 & \;0 & \;0 & \;0 & \;0 & \;0\\ \;0 & \;0 & \;0 & \;0 & \;0 & \;0 & \;0 & \;0 & \;0 & \;0.29 & \;0 & \;0 & \;0 & \;0 & \;0 & \;0 & \;0 & \;0 & \;0 & \;2 & \;0.50 & \;0.50\\ \;0 & \;0 & \;0 & \;0 & \;0 & \;0 & \;0 & \;0 & \;0 & \;0 & \;0 & \;0 & \;0 & \;0 & \;0 & \;0 & \;0 & \;0 & \;0 & \;0.50 & \;0 & \;0\\ \;0 & \;0 & \;0 & \;0 & \;0 & \;0 & \;0 & \;0 & \;0 & \;0 & \;0 & \;0 & \;0 & \;0 & \;0 & \;0 & \;0 & \;0 & \;0 & \;0.50 & \;0 & \;0 \end{array}\right]$$

The characteristic equation of $H^{R}\left(G_{1}\right)$ is $\left|H^{R}\left(G_{1}\right)-\lambda I\right|=0$ , where I is the unit matrix of order 22. That is,

$$\lambda^{22}-13\lambda^{21}+69.1102721\lambda^{20}-187.008061\lambda^{19}+246.028735\lambda^{18}-62.1478601\lambda^{17}-203.924439\lambda^{16}+160.037639\lambda^{15}+64.7657396\lambda^{14}-73.1084426\lambda^{13}-17.7171908\lambda^{12}+12.6502404\lambda^{11}+3.38968170\lambda^{10}-0.780712562\lambda^{9}-0.306360974\lambda^{8}-\left(3.93618005\times 10^{-3}\right)\lambda^{7}+\left(8.80093471\times 10^{-3}\right)\lambda^{6}+\left(1.28224132\times 10^{-3}\right)\lambda^{5}+\left(7.01541082\times 10^{-5}\right)\lambda^{4}+\left(1.31566694\times 10^{-6}\right)\lambda^{3}-\left(6.38634078\times 10^{-24}\right)\lambda^{2}-\left(6.05857299\times 10^{-39}\right)\lambda +0=0$$

The eigenvalues ${\left\{\lambda_{i_{H}}^{R}\right\}}_{i=1}^{22}$ of $H^{R}\left(G_{1}\right)$ are 2.26621801, 2.22317239, 2.09184158, 2.13731344, 2.12511292, 2.15826628, 1.20486131, 0.381006799, 0.350094262, 0.272946948, −0.534739512, −0.480749173, −0.365871874, −0.0423106343, −0.225119620, −0.196839452, −0.0852716745, −0.123089003, −0.156843002, −6.54760926 × 10^−17^, 7.03301636 × 10^−17^ and 0.

Hence, the Roman Harmonic energy $E_{H}^{R}\left(G_{1}\right)$ is

$$E_{H}^{R}\left(G_{1}\right)=\sum_{i=1}^{\left|V\right|}\left|\lambda_{i_{H}}^{R}\right|=\sum_{i=1}^{22}\left|\lambda_{i_{H}}^{R}\right|$$

$$=|2.26621801|+|2.22317239|+|2.09184158|+|2.13731344|+|2.12511292|+|2.15826628|+|1.20486131|+|0.381006799|+|0.350094262|+|0.272946948|+|-0.534739512|+|-0.480749173|+|-0.365871874|+|-0.0423106343|+|-0.225119620|+|-0.196839452|+|-0.0852716745|+|-0.123089003|+|-0.156843002|+|-6.54760926\times 10^{-17}|+|7.03301636\times 10^{-17}|+|0|=17.4217$$

The Roman Atom-bond connectivity matrix $ABC^{R}\left(G_{1}\right)$ is obtained as

$$\left[\begin{array}{cccccccccccccccccccccc} \;0 & \;0.71 & \;0 & \;0 & \;0 & \;0 & \;0 & \;0 & \;0 & \;0 & \;0 & \;0 & \;0 & \;0 & \;0 & \;0 & \;0 & \;0 & \;0 & \;0 & \;0 & \;0\;\\ \;0.71 & \;2 & \;0.65 & \;0 & \;0 & \;0 & \;0 & \;0 & \;0 & \;0 & \;0 & \;0 & \;0.65 & \;0 & \;0 & \;0 & \;0 & \;0 & \;0 & \;0 & \;0 & \;0\\ \;0 & \;0.65 & \;0 & \;0.65 & \;0 & \;0 & \;0 & \;0 & \;0 & \;0 & \;0 & \;0 & \;0 & \;0.82 & \;0 & \;0 & \;0 & \;0 & \;0 & \;0 & \;0 & \;0\\ \;0 & \;0 & \;0.65 & \;0 & \;0.71 & \;0 & \;0 & \;0 & \;0 & \;0 & \;0.61 & \;0 & \;0 & \;0 & \;0 & \;0 & \;0 & \;0 & \;0 & \;0 & \;0 & \;0\\ \;0 & \;0 & \;0 & \;0.71 & \;0 & \;0.71 & \;0 & \;0 & \;0 & \;0 & \;0 & \;0 & \;0 & \;0 & \;0 & \;0 & \;0 & \;0 & \;0 & \;0 & \;0 & \;0\\ \;0 & \;0 & \;0 & \;0 & \;0.71 & \;2 & \;0.65 & \;0 & \;0 & \;0 & \;0 & \;0 & \;0 & \;0 & \;0 & \;0 & \;0.61 & \;0.87 & \;0 & \;0 & \;0 & \;0\\ \;0 & \;0 & \;0 & \;0 & \;0 & \;0.65 & \;0 & \;0.65 & \;0 & \;0 & \;0 & \;0 & \;0 & \;0 & \;0 & \;0 & \;0 & \;0 & \;0 & \;0 & \;0 & \;0\\ \;0 & \;0 & \;0 & \;0 & \;0 & \;0 & \;0.65 & \;2 & \;0.71 & \;0 & \;0 & \;0 & \;0 & \;0 & \;0 & \;0 & \;0 & \;0 & \;0.87 & \;0 & \;0 & \;0\\ \;0 & \;0 & \;0 & \;0 & \;0 & \;0 & \;0 & \;0.71 & \;0 & \;0.71 & \;0 & \;0 & \;0 & \;0 & \;0 & \;0 & \;0 & \;0 & \;0 & \;0 & \;0 & \;0\\ \;0 & \;0 & \;0 & \;0 & \;0 & \;0 & \;0 & \;0 & \;0.71 & \;0 & \;0.61 & \;0 & \;0 & \;0 & \;0 & \;0 & \;0.61 & \;0 & \;0 & \;0.65 & \;0 & \;0\\ \;0 & \;0 & \;0 & \;0.61 & \;0 & \;0 & \;0 & \;0 & \;0 & \;0.61 & \;2 & \;0.65 & \;0 & \;0 & \;0 & \;0 & \;0 & \;0 & \;0 & \;0 & \;0 & \;0\\ \;0 & \;0 & \;0 & \;0 & \;0 & \;0 & \;0 & \;0 & \;0 & \;0 & \;0.65 & \;0 & \;0.67 & \;0 & \;0 & \;0 & \;0 & \;0 & \;0 & \;0 & \;0 & \;0\\ \;0 & \;0.65 & \;0 & \;0 & \;0 & \;0 & \;0 & \;0 & \;0 & \;0 & \;0 & \;0.67 & \;0 & \;0 & \;0 & \;0 & \;0 & \;0 & \;0 & \;0 & \;0 & \;0\\ \;0 & \;0 & \;0.82 & \;0 & \;0 & \;0 & \;0 & \;0 & \;0 & \;0 & \;0 & \;0 & \;0 & \;1 & \;0 & \;0 & \;0 & \;0 & \;0 & \;0 & \;0 & \;0\\ \;0 & \;0 & \;0 & \;0 & \;0 & \;0 & \;0 & \;0 & \;0 & \;0 & \;0 & \;0 & \;0 & \;0 & \;0 & \;0.82 & \;0 & \;0 & \;0 & \;0 & \;0 & \;0\\ \;0 & \;0 & \;0 & \;0 & \;0 & \;0 & \;0 & \;0 & \;0 & \;0 & \;0 & \;0 & \;0 & \;0 & \;0.82 & \;2 & \;0.65 & \;0 & \;0 & \;0 & \;0 & \;0\\ \;0 & \;0 & \;0 & \;0 & \;0 & \;0.61 & \;0 & \;0 & \;0 & \;0.61 & \;0 & \;0 & \;0 & \;0 & \;0 & \;0.65 & \;0 & \;0 & \;0 & \;0 & \;0 & \;0\\ \;0 & \;0 & \;0 & \;0 & \;0 & \;0.87 & \;0 & \;0 & \;0 & \;0 & \;0 & \;0 & \;0 & \;0 & \;0 & \;0 & \;0 & \;0 & \;0 & \;0 & \;0 & \;0\\ \;0 & \;0 & \;0 & \;0 & \;0 & \;0 & \;0 & \;0.87 & \;0 & \;0 & \;0 & \;0 & \;0 & \;0 & \;0 & \;0 & \;0 & \;0 & \;0 & \;0 & \;0 & \;0\\ \;0 & \;0 & \;0 & \;0 & \;0 & \;0 & \;0 & \;0 & \;0 & \;0.65 & \;0 & \;0 & \;0 & \;0 & \;0 & \;0 & \;0 & \;0 & \;0 & \;2 & \;0.82 & \;0.82\\ \;0 & \;0 & \;0 & \;0 & \;0 & \;0 & \;0 & \;0 & \;0 & \;0 & \;0 & \;0 & \;0 & \;0 & \;0 & \;0 & \;0 & \;0 & \;0 & \;0.82 & \;0 & \;0\\ \;0 & \;0 & \;0 & \;0 & \;0 & \;0 & \;0 & \;0 & \;0 & \;0 & \;0 & \;0 & \;0 & \;0 & \;0 & \;0 & \;0 & \;0 & \;0 & \;0.82 & \;0 & \;0 \end{array}\right]$$

The characteristic equation of $ABC^{R}\left(G_{1}\right)$ is $\left|ABC^{R}\left(G_{1}\right)-\lambda I\right|=0$ , where I is the unit matrix of order 22. That is,

$$\lambda^{22}-13\lambda^{21}+60.05555556\lambda^{20}-83.47222222\lambda^{19}-193.451968\lambda^{18}+663.464699\lambda^{17}-34.4877212\lambda^{16}-1613.58418\lambda^{15}+740.764567\lambda^{14}+2104.25422\lambda^{13}-963.137266\lambda^{12}-1694.80927\lambda^{11}+400.651166\lambda^{10}+779.181624\lambda^{9}+6.34378928\lambda^{8}-167.718852\lambda^{7}-32.2084202\lambda^{6}+9.97779959\lambda^{5}+3.51284669\lambda^{4}+0.263888889\lambda^{3}+\left(7.89996078\times 10^{-17}\right)\lambda^{2}+\left(5.69122937\times 10^{-33}\right)\lambda +\left(2.93891698\times 10^{-65}\right)=0$$

The eigenvalues ${\left\{\lambda_{i_{ABC}}^{R}\right\}}_{i=1}^{22}$ of $ABC^{R}\left(G_{1}\right)$ are 2.90381715, 2.71556661, 2.60127176, 2.57429276, 2.39331410, 2.38708605, 1.45335523, −1.33310272, 0.761775276, 0.658711836, 0.365875884, −1.13409826, −0.832854551, −0.133135441, −0.274429204, −0.389579658, −0.480360620, −0.573261747, −0.664244460, −1.20728736 × 10^−16^, −1.78638199 × 10^−16^, and−5.16394048 × 10^−33^.

The Roman Atom-bond connectivity energy $E_{ABC}^{R}\left(G_{1}\right)$ is

$$E_{ABC}^{R}\left(G_{1}\right)=\sum_{i=1}^{\left|V\right|}\left|\lambda_{i_{ABC}}^{R}\right|=\sum_{i=1}^{22}\left|\lambda_{i_{ABC}}^{R}\right|$$

=∣2.90381715∣+∣2.71556661∣+∣2.60127176∣+∣2.57429276∣+∣2.39331410∣+∣2.38708605∣+∣1.45335523∣+∣−1.3331027∣+∣0.76177528∣+∣0.65871184∣+∣0.365875884∣+∣−1.13409826∣+∣−0.832854551∣+∣−0.133135441∣+∣−0.274429204∣+∣−0.389579658∣+∣−0.480360620∣+∣−0.573261747∣+∣−0.664244460∣+∣−1.20728736×10−16∣+∣−1.78638199×10−16∣+∣−5.16394048×10−33∣=24.6301

Likewise, the Roman Geometric-Arithmetic matrix $GA^{R}\left(G_{1}\right)$ is obtained as

$$\left[\begin{array}{cccccccccccccccccccccc} \;0 & \;0.94 & \;0 & \;0 & \;0 & \;0 & \;0 & \;0 & \;0 & \;0 & \;0 & \;0 & \;0 & \;0 & \;0 & \;0 & \;0 & \;0 & \;0 & \;0 & \;0 & \;0\;\\ \;0.94 & \;2 & \;0.99 & \;0 & \;0 & \;0 & \;0 & \;0 & \;0 & \;0 & \;0 & \;0 & \;0.99 & \;0 & \;0 & \;0 & \;0 & \;0 & \;0 & \;0 & \;0 & \;0\\ \;0 & \;0.99 & \;0 & \;0.99 & \;0 & \;0 & \;0 & \;0 & \;0 & \;0 & \;0 & \;0 & \;0 & \;0.87 & \;0 & \;0 & \;0 & \;0 & \;0 & \;0 & \;0 & \;0\\ \;0 & \;0 & \;0.99 & \;0 & \;0.94 & \;0 & \;0 & \;0 & \;0 & \;0 & \;1 & \;0 & \;0 & \;0 & \;0 & \;0 & \;0 & \;0 & \;0 & \;0 & \;0 & \;0\\ \;0 & \;0 & \;0 & \;0.94 & \;0 & \;0.94 & \;0 & \;0 & \;0 & \;0 & \;0 & \;0 & \;0 & \;0 & \;0 & \;0 & \;0 & \;0 & \;0 & \;0 & \;0 & \;0\\ \;0 & \;0 & \;0 & \;0 & \;0.94 & \;2 & \;0.98 & \;0 & \;0 & \;0 & \;0 & \;0 & \;0 & \;0 & \;0 & \;0 & \;1 & \;0.80 & \;0 & \;0 & \;0 & \;0\\ \;0 & \;0 & \;0 & \;0 & \;0 & \;0.99 & \;0 & \;0.99 & \;0 & \;0 & \;0 & \;0 & \;0 & \;0 & \;0 & \;0 & \;0 & \;0 & \;0 & \;0 & \;0 & \;0\\ \;0 & \;0 & \;0 & \;0 & \;0 & \;0 & \;0.99 & \;2 & \;0.94 & \;0 & \;0 & \;0 & \;0 & \;0 & \;0 & \;0 & \;0 & \;0 & \;0.80 & \;0 & \;0 & \;0\\ \;0 & \;0 & \;0 & \;0 & \;0 & \;0 & \;0 & \;0.94 & \;0 & \;0.94 & \;0 & \;0 & \;0 & \;0 & \;0 & \;0 & \;0 & \;0 & \;0 & \;0 & \;0 & \;0\\ \;0 & \;0 & \;0 & \;0 & \;0 & \;0 & \;0 & \;0 & \;0.94 & \;0 & \;1 & \;0 & \;0 & \;0 & \;0 & \;0 & \;1 & \;0 & \;0 & \;0.99 & \;0 & \;0\\ \;0 & \;0 & \;0 & \;1 & \;0 & \;0 & \;0 & \;0 & \;0 & \;1 & \;2 & \;0.99 & \;0 & \;0 & \;0 & \;0 & \;0 & \;0 & \;0 & \;0 & \;0 & \;0\\ \;0 & \;0 & \;0 & \;0 & \;0 & \;0 & \;0 & \;0 & \;0 & \;0 & \;0.99 & \;0 & \;1 & \;0 & \;0 & \;0 & \;0 & \;0 & \;0 & \;0 & \;0 & \;0\\ \;0 & \;0.99 & \;0 & \;0 & \;0 & \;0 & \;0 & \;0 & \;0 & \;0 & \;0 & \;1 & \;0 & \;0 & \;0 & \;0 & \;0 & \;0 & \;0 & \;0 & \;0 & \;0\\ \;0 & \;0 & \;0.87 & \;0 & \;0 & \;0 & \;0 & \;0 & \;0 & \;0 & \;0 & \;0 & \;0 & \;1 & \;0 & \;0 & \;0 & \;0 & \;0 & \;0 & \;0 & \;0\\ \;0 & \;0 & \;0 & \;0 & \;0 & \;0 & \;0 & \;0 & \;0 & \;0 & \;0 & \;0 & \;0 & \;0 & \;0 & \;0.87 & \;0 & \;0 & \;0 & \;0 & \;0 & \;0\\ \;0 & \;0 & \;0 & \;0 & \;0 & \;0 & \;0 & \;0 & \;0 & \;0 & \;0 & \;0 & \;0 & \;0 & \;0.87 & \;2 & \;0.99 & \;0 & \;0 & \;0 & \;0 & \;0\\ \;0 & \;0 & \;0 & \;0 & \;0 & \;1 & \;0 & \;0 & \;0 & \;1 & \;0 & \;0 & \;0 & \;0 & \;0 & \;0.99 & \;0 & \;0 & \;0 & \;0 & \;0 & \;0\\ \;0 & \;0 & \;0 & \;0 & \;0 & \;0.80 & \;0 & \;0 & \;0 & \;0 & \;0 & \;0 & \;0 & \;0 & \;0 & \;0 & \;0 & \;0 & \;0 & \;0 & \;0 & \;0\\ \;0 & \;0 & \;0 & \;0 & \;0 & \;0 & \;0 & \;0.80 & \;0 & \;0 & \;0 & \;0 & \;0 & \;0 & \;0 & \;0 & \;0 & \;0 & \;0 & \;0 & \;0 & \;0\\ \;0 & \;0 & \;0 & \;0 & \;0 & \;0 & \;0 & \;0 & \;0 & \;0.99 & \;0 & \;0 & \;0 & \;0 & \;0 & \;0 & \;0 & \;0 & \;0 & \;2 & \;0.87 & \;0.87\\ \;0 & \;0 & \;0 & \;0 & \;0 & \;0 & \;0 & \;0 & \;0 & \;0 & \;0 & \;0 & \;0 & \;0 & \;0 & \;0 & \;0 & \;0 & \;0 & \;0.87 & \;0 & \;0\\ \;0 & \;0 & \;0 & \;0 & \;0 & \;0 & \;0 & \;0 & \;0 & \;0 & \;0 & \;0 & \;0 & \;0 & \;0 & \;0 & \;0 & \;0 & \;0 & \;0.87 & \;0 & \;0 \end{array}\right]$$

The characteristic equation of $GA^{R}\left(G_{1}\right)$ is $\left|GA^{R}\left(G_{1}\right)-\lambda I\right|=0$ , where I is the unit matrix of order 22. That is,

$$\lambda^{22}-13\lambda^{21}+50.4388209\lambda^{20}+27.4377098\lambda^{19}-600.929811\lambda^{18}+779.92926\lambda^{17}+2394.63818\lambda^{16}-5106.13723\lambda^{15}-4561.92087\lambda^{14}+13313.9587\lambda^{13}+5495.38568\lambda^{12}-17577.9555\lambda^{11}-5794.92002\lambda^{10}+11689.9637\lambda^{9}+4496.19672\lambda^{8}-3264.70341\lambda^{7}-1629.41884\lambda^{6}+128.542648\lambda^{5}+132.335742\lambda^{4}+12.9255852\lambda^{3}-\left(1.01999353\times 10^{-15}\right)\lambda^{2}+\left(2.57482969\times 10^{-32}\right)\lambda +\left(3.96715328\times 10^{-65}\right)=0$$

The eigenvalues ${\left\{\lambda_{i_{GA}}^{R}\right\}}_{i=1}^{22}$ of $GA^{R}\left(G_{1}\right)$ are −2.08575025, 3.54501997, 3.18769045, 3.00624527, 2.79641682, 2.64943222, 2.47700180, 1.57539588, −1.67925677, 0.958350468, 0.862354795, −1.26885654, 0.334323419, −0.95761194, −0.794245516, −0.640351481, −0.528102676, −0.298429079, −0.139626840, 3.94563773 × 10^−17^, 3.94563773 × 10^−17^ and −1.54074396 × 10^−33^.

Thus, the Roman Geometric-Arithmetic energy is

$$E_{GA}^{R}\left(G_{1}\right)=\sum_{i=1}^{\left|V\right|}\left|\lambda_{i_{GA}}^{R}\right|=\sum_{i=1}^{22}\left|\lambda_{i_{GA}}^{R}\right|$$

$$=|-2.08575025|+|3.54501997|+|3.18769045|+|3.00624527|+|2.79641682|+|2.64943222|+|2.47700180|+|1.57539588|+|-1.67925677|+|0.958350468|+|0.862354795|+|-1.26885654|+|0.334323419|+|-0.95761194|+|-0.794245516|+|-0.640351481|+|-0.528102676|+|-0.298429079|+|-0.139626840|+|3.94563773\times 10^{-17}|+|3.94563773\times 10^{-17}|+|-1.54074396\times 10^{-33}|=29.7845$$

Hence the proof.

The Roman domination number ( $\gamma_{R}$ ), classical graph energies ( $E,E_{L},E_{R},E_{H},E_{ABC}$ and $E_{GA}$ ) and Roman energies ( $E^{R},E_{L}^{R},E_{R}^{R},E_{H}^{R},E_{ABC}^{R}$ and $E_{GA}^{R}$ ) of the remaining 20 anti-Alzheimer’s herbal compounds are computed using python programming and the results are presented in Tables 2–4. The calculated Roman domination numbers, classical graph energies and Roman graph energies will serve as the foundation for the subsequent QSPR analysis, providing deeper insights into the relationship between molecular structure and biological activity. The chemical structures and Roman domination representations of the corresponding isomorphic molecular graphs for the remaining 20 anti-Alzheimer’s herbal compounds are provided in the Supplementary Material.

**TABLE 2:** Order, size and Roman domination number of the isomorphic molecular graph of anti-Alzheimer’s herbal compounds.

Herbal compound	$\left|V\right|$	$\left|E\right|$	$\gamma_{R}$
Huperzine A	22	29	13
Curcumin	29	40	18
Ginkgolide	34	42	19
Bilobalide	27	33	16
Ginsenoside Rg1	70	76	46
Resveratrol	20	28	12
Epigallocatechin-3-gallate	41	54	28
Quercetin	27	37	18
Baicalein	23	33	15
Asiaticoside	82	91	54
Catalpol	34	38	22
Ferulic acid	16	21	10
Salvianolic acid	43	59	28
Schisandrin	32	40	22
Rhynchophylline	29	40	20
Glycyrrhizin	69	80	44
γ-linolenic acid	21	24	14
Crocin	82	94	56
Isorhamnetin	27	37	19
Allicin	9	11	6
Gingerol	23	27	15

**TABLE 3:** Classical graph energies of the isomorphic molecular graph of anti-Alzheimer’s herbal compounds.

Herbal compound	E	$E_{L}$	$E_{R}$	$E_{H}$	$E_{ABC}$	$E_{GA}$
Huperzine A	34.3620	55.3636	10.4966	9.5596	19.2852	25.2630
Curcumin	53.0028	77.2414	14.4753	13.9756	24.8656	35.9167
Ginkgolide	46.5167	81.5294	16.8219	15.3796	30.0210	38.7652
Bilobalide	37.1615	63.5556	13.1520	12.0350	23.3138	30.0474
Ginsenoside Rg1	87.8368	149.8286	41.0031	38.0700	63.0826	81.5509
Resveratrol	37.3044	53.2000	10.2312	9.9217	17.4673	25.5644
Epigallocatechin-3-gallate	68.4969	105.3659	22.5321	21.6360	36.0824	51.6532
Quercetin	47.2727	71.2593	14.2992	13.7406	23.5320	34.0513
Baicalein	42.5686	63.1304	11.4464	11.0766	20.1446	29.4721
Asiaticoside	106.8511	179.7805	48.6417	45.7908	74.2672	98.9473
Catalpol	44.4298	73.7647	19.9855	18.8917	30.3737	40.9799
Ferulic acid	28.3786	39.3750	8.5181	8.1955	13.6748	19.6166
Salvianolic acid	74.4780	115.2558	22.0338	21.0245	36.9210	52.5710
Schisandrin	49.3070	77.5000	18.3323	17.3121	28.0568	39.0014
Rhynchophylline	49.4599	77.2414	14.1963	13.4832	26.2338	36.5573
Glycyrrhizin	95.7952	157.6812	39.0162	36.3389	62.7377	82.2327
γ-linolenic acid	31.9342	45.7143	11.8707	11.5934	18.1137	25.2894
Crocin	116.5436	185.7073	47.5990	45.5355	72.3650	99.6338
Isorhamnetin	47.2092	71.2593	14.2992	13.7405	23.5320	34.0513
Allicin	15.5348	19.5556	4.2109	4.1003	7.3426	10.0843
Gingerol	35.0696	51.6522	13.2455	12.7965	19.9166	27.9585

**TABLE 4:** Roman energies of the isomorphic molecular graph of anti-Alzheimer’s herbal compounds.

Herbal compound	$E^{R}$	$E_{L}^{R}$	$E_{R}^{R}$	$E_{H}^{R}$	$E_{ABC}^{R}$	$E_{GA}^{R}$
Huperzine A	45.1296	73.3636	17.9872	17.4217	24.6301	29.7845
Curcumin	66.5497	99.2414	25.3791	25.0589	32.8265	41.7363
Ginkgolide	63.1427	110.5294	27.0789	26.2104	37.5207	45.2507
Bilobalide	51.4368	88.5556	21.6781	21.0097	29.4531	35.2526
Ginsenoside Rg1	124.9425	200.8286	66.1705	64.4381	83.4936	99.4874
Resveratrol	45.7874	66.2000	17.4145	17.2161	22.6181	29.3747
Epigallocatechin-3-gallate	89.4491	136.3659	39.1147	38.4836	48.8531	61.3853
Quercetin	60.9440	92.2593	24.7818	24.4071	31.4479	40.0196
Baicalein	54.2303	81.1304	20.7828	20.5382	26.9751	34.4137
Asiaticoside	148.5861	235.7805	78.3375	76.6201	98.2212	119.4579
Catalpol	62.6139	99.7647	31.9158	31.2474	40.0326	48.9767
Ferulic acid	35.3938	50.3750	14.3872	14.1667	18.0653	22.8620
Salvianolic acid	95.6913	147.2558	38.2904	37.6784	49.2907	62.3634
Schisandrin	65.9585	101.5000	30.6399	30.0145	37.8126	46.8755
Rhynchophylline	62.4522	96.2414	25.4212	25.0059	34.5461	43.3004
Glycyrrhizin	128.4360	203.6812	63.4952	61.9284	81.8867	98.8384
γ-linolenic acid	42.2565	58.7143	20.9834	20.7757	25.3555	30.9798
Crocin	158.8990	242.7073	79.1212	77.8324	97.6712	120.1361
Isorhamnetin	61.9874	93.2593	25.6695	25.3129	32.2697	40.6842
Allicin	20.2234	26.5556	7.8267	7.7753	9.8808	12.0996
Gingerol	46.8662	72.6521	24.3623	24.0791	28.7168	34.9153

## 7 QSPR analysis of physicochemical properties of Anti-Alzheimer’s herbal compounds

In this section, Quantitative Structure-Property Relationship (QSPR) analysis is conducted to examine the association between the calculated graph energies and physicochemical properties of anti-Alzheimer’s herbal compounds. The experimental values of boiling point (BP), enthalpy of vaporization (EV), molar volume (MV), molar refractivity (MR), polarizability (P), polar surface area (PSA), molecular weight (MW) and surface area (SA) are gathered from the ChemSpider database and are summarized in Table 5. To investigate the relationship between graph energies and physicochemical properties, three regression models, specifically linear, quadratic and cubic are employed in QSPR analysis. The general forms of these regression equations are given below:

$$P=c_{1}\left(E\right)+c_{2}$$

$$P=c_{1}{\left(E\right)}^{2}+c_{2}\left(E\right)+c_{3}$$

$$P=c_{1}{\left(E\right)}^{3}+c_{2}{\left(E\right)}^{2}+c_{3}\left(E\right)+c_{4}$$

where P is the physicochemical property, E is the graph energy and $c_{1},c_{2},c_{3}$ and $c_{4}$ are constants. The QSPR regression equations derived from classical and Roman energies are constructed using SPSS (Statistical Package for Social Sciences) software.

**TABLE 5:** Experimental values of the physicochemical properties of anti-Alzheimer’s herbal compounds.

Herbal compound	$BP\;\left({}^{\circ}C\right)$	EV $\left(kJ/mol\right)$	MV $\left(cm^{3}\right)$	MR $\left(cm^{3}\right)$	P $\left(cm^{3}\right)$	$PSA\left({Å}^{2}\right)$	MW $\left(g/mol\right)$	$SA\;\left({Å}^{2}\right)$
Huperzine A	505.0	77.5	201.8	71.5	28.3	55	242.322	106.411
Curcumin	591.4	91.5	287.9	104.0	41.2	93	368.385	156.532
Ginkgolide	710.1	118.7	260.2	92.7	36.7	129	408.403	166.165
Bilobalide	651.7	110.0	208.6	71.9	28.5	119	326.301	131.239
Ginsenoside Rg1	898.5	148.3	600.4	205.9	81.6	239	801.024	331.374
Resveratrol	449.1	73.5	167.9	69.3	27.5	61	228.247	98.911
Epigallocatechin-3-gallate	909.1	136.7	241.2	108.4	43.0	197	458.375	184.742
Quercetin	642.4	98.3	168.0	73.3	29.1	127	302.238	122.108
Baicalein	575.9	89.5	174.6	69.9	27.7	87	270.24	112.519
Asiaticoside	1017.5	168.3	664.2	236.2	93.6	315	959.133	391.529
Catalpol	675.6	113.5	209.8	79.2	31.4	162	362.331	142.355
Ferulic acid	372.3	65.3	147.5	52.3	20.7	67	194.186	81.065
Salvianolic acid	858.7	130.7	312.9	132.5	52.5	185	494.452	204.082
Schisandrin	576.7	90.8	381.0	118.6	47.0	76	432.513	183.337
Rhynchophylline	560.8	84.3	310.2	105.6	41.9	68	384.476	165.423
Glycyrrhizin	971.4	160.4	572.6	201.4	79.8	267	822.942	337.426
γ-linolenic acid	379.5	68.9	301.1	87.2	34.6	37	278.436	123.830
Crocin	1169.0	195.2	634.3	231.3	91.7	391	976.972	389.932
Isorhamnetin	599.4	92.5	193.5	78.1	31.0	116	316.265	128.792
Allicin	248.6	46.6	141.2	46.2	18.3	62	162.279	62.082
Gingerol	453.0	75.1	271.7	82.9	32.9	67	294.391	126.309

## 8 Results and discussion

**FIGURE 4:**
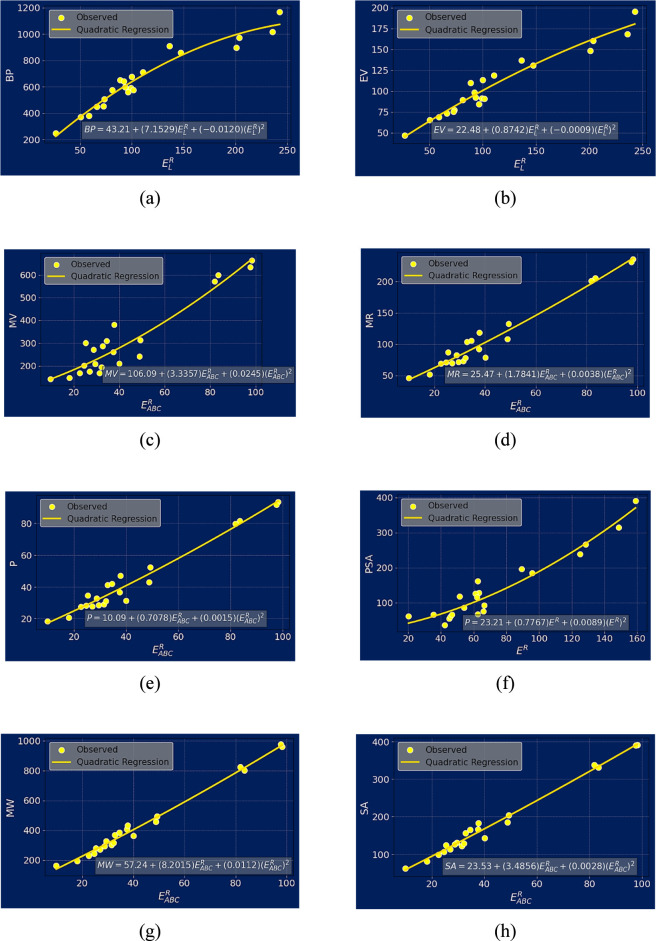
Scatter plots of the most significant quadratic QSPR regression equations **(a)** $E_{L}^{R}$ with BP , **(b)** $E_{L}^{R}$ with EV , **(c)** $E_{ABC}^{R}$ with MV , **(d)** $E_{ABC}^{R}$ with MR , **(e)** $E_{ABC}^{R}$ with P , **(f)** $E^{R}$ with PSA , **(g)** $E_{ABC}^{R}$ with MW and **(h)** $E_{ABC}^{R}$ with SA .

The QSPR modeling results indicate that both classical and Roman energies exhibit strong correlations $\left(r\geq 0.9\right)$ with the physicochemical properties of anti-Alzheimer’s herbal compounds, as shown in Table 6. The detailed comparative analysis presented in Table 6 indicates that the correlation coefficients associated with Roman energy descriptors are consistently greater than or equal to those of the classical graph energy descriptors across all three regression models. This consistent superiority of Roman energies underscores the significance of Roman domination functions as an advancement over classical graph energies, providing enhanced predictive power in QSPR analysis. Furthermore, among the three regression analysis, the quadratic regression provides the best fit, showing a higher correlation coefficient and a lower standard error than the linear and cubic regression models. Table 7 presents the most significant quadratic regression equations for all properties along with their statistical parameters (correlation coefficient $\left(r\right)$ , coefficient of determination $\left(r^{2}\right),$ standard error $\left(S.E.\right)$ , significance value $\left(p\right)$ ), while Figure 4 illustrates the corresponding scatter plots. These scatter plots further support the findings by exhibiting a strong agreement between the observed and predicted physicochemical property values, with data points closely distributed around the quadratic regression curve, thereby confirming the stability, consistency, and predictive robustness of the developed QSPR models. The most prominent Roman energies for exploring the physicochemical properties of anti-Alzheimer’s herbal compounds using the quadratic QSPR regression equations are given below.

-
$E_{ABC}^{R}$ exhibits excellent correlations with molar volume (0.948), molar refractivity (0.982), polarizability (0.982), molecular weight (0.995), and surface area (0.993).
-
$E_{L}^{R}$ shows the strongest correlations with boiling point (0.973) and enthalpy of vaporization (0.971).
-
$E^{R}$ yields a good correlation with polar surface area (0.962).

**TABLE 6:** Correlation coefficient of graph energies with the physicochemical properties of anti-Alzheimer’s herbal compounds.

Energy	Regression	BP	EV	MV	MR	P	PSA	MW	SA
E	Linear	0.950	0.953	0.900	0.962	0.962	0.946	0.973	0.971
	Quadratic	0.960	0.953	0.912	0.967	0.967	0.960	0.979	0.975
	Cubic	0.962	0.955	0.914	0.970	0.970	0.960	0.980	0.976
$E^{R}$	Linear	0.950	0.959	0.915	0.971	0.971	0.954	0.984	0.981
	Quadratic	0.966	0.962	0.925	0.975	0.975	0.962	0.988	0.984
	Cubic	0.968	0.963	0.927	0.976	0.976	0.962	0.988	0.984
$E_{L}$	Linear	0.956	0.963	0.913	0.969	0.969	0.949	0.984	0.981
	Quadratic	0.970	0.965	0.930	0.977	0.977	0.960	0.990	0.987
	Cubic	0.971	0.966	0.930	0.977	0.977	0.960	0.991	0.987
$E_{L}^{R}$	Linear	0.957	0.967	0.916	0.969	0.969	0.952	0.986	0.983
	Quadratic	0.973	0.971	0.933	0.977	0.977	0.961	0.992	0.989
	Cubic	0.974	0.971	0.933	0.977	0.977	0.961	0.993	0.989
$E_{R}$	Linear	0.907	0.935	0.945	0.978	0.978	0.945	0.992	0.990
	Quadratic	0.941	0.949	0.947	0.978	0.978	0.945	0.992	0.990
	Cubic	0.944	0.951	0.948	0.978	0.978	0.945	0.992	0.990
$E_{R}^{R}$	Linear	0.913	0.937	0.945	0.980	0.980	0.947	0.992	0.990
	Quadratic	0.941	0.949	0.947	0.980	0.981	0.948	0.993	0.990
	Cubic	0.944	0.951	0.948	0.981	0.981	0.948	0.993	0.990
$E_{H}$	Linear	0.907	0.935	0.944	0.978	0.978	0.948	0.992	0.989
	Quadratic	0.939	0.948	0.946	0.978	0.978	0.948	0.992	0.989
	Cubic	0.943	0.950	0.947	0.978	0.978	0.948	0.992	0.989
$E_{H}^{R}$	Linear	0.913	0.937	0.945	0.980	0.980	0.948	0.992	0.990
	Quadratic	0.940	0.948	0.946	0.980	0.980	0.949	0.992	0.990
	Cubic	0.943	0.950	0.947	0.981	0.981	0.949	0.992	0.990
$E_{ABC}$	Linear	0.921	0.944	0.942	0.979	0.979	0.945	0.994	0.992
	Quadratic	0.952	0.958	0.947	0.981	0.981	0.946	0.995	0.993
	Cubic	0.954	0.959	0.948	0.981	0.981	0.946	0.995	0.993
$E_{ABC}^{R}$	Linear	0.921	0.944	0.945	0.981	0.981	0.947	0.995	0.993
	Quadratic	0.952	0.958	0.948	0.982	0.982	0.948	0.995	0.993
	Cubic	0.954	0.959	0.948	0.982	0.982	0.948	0.995	0.993
$E_{GA}$	Linear	0.927	0.947	0.935	0.979	0.979	0.950	0.993	0.991
	Quadratic	0.955	0.958	0.940	0.980	0.980	0.952	0.994	0.991
	Cubic	0.956	0.959	0.941	0.981	0.981	0.952	0.994	0.991
$E_{GA}^{R}$	Linear	0.927	0.947	0.939	0.980	0.980	0.950	0.994	0.992
	Quadratic	0.955	0.958	0.944	0.981	0.981	0.952	0.994	0.992
	Cubic	0.956	0.959	0.944	0.982	0.982	0.952	0.995	0.992

**TABLE 7:** The most significant quadratic regression models for the physicochemical properties of anti-Alzheimer’s herbal compounds.

Property	Quadratic regression equation	r	$r^{2}$	S.E.	p
BP	$BP=43.2111+7.1529E_{L}^{R}-0.0120{\left(E_{L}^{R}\right)}^{2}$	0.973	0.947	57.568	0.000
EV	$EV=22.4812+0.8742E_{L}^{R}-0.0009{\left(E_{L}^{R}\right)}^{2}$	0.971	0.943	9.651	0.000
MV	$MV=106.0879+3.3357E_{ABC}^{R}+0.0245{\left(E_{ABC}^{R}\right)}^{2}$	0.948	0.899	55.934	0.000
MR	$MR=25.4700+1.7841E_{ABC}^{R}+0.0038{\left(E_{ABC}^{R}\right)}^{2}$	0.982	0.964	11.555	0.000
P	$P=10.0894+0.7078E_{ABC}^{R}+0.0015{\left(E_{ABC}^{R}\right)}^{2}$	0.982	0.964	4.576	0.000
PSA	$PSA=23.2101+0.7767E^{R}+0.0089{\left(E^{R}\right)}^{2}$	0.962	0.926	27.258	0.000
MW	$MW=57.2355+8.2015E_{ABC}^{R}+0.0112{\left(E_{ABC}^{R}\right)}^{2}$	0.995	0.990	25.491	0.000
SA	$SA=23.5287+3.4856E_{ABC}^{R}+0.0028{\left(E_{ABC}^{R}\right)}^{2}$	0.993	0.986	12.369	0.000

Thus, the results indicate the suitability of Roman energy descriptors for exploring the targeted physicochemical properties. In particular, $E_{ABC}^{R}$ ​ emerges as a highly effective descriptor for predicting the size-related and shape-related properties, including molar volume, molar refractivity, molecular weight and surface area. Similarly, $E_{L}^{R}$ ​ is identified as a reliable predictor for the thermodynamic properties such as boiling point and enthalpy of vaporization, while $E^{R}$ shows particular relevance for modeling the polar surface area. These findings suggest that the developed quadratic QSPR models can be selectively employed depending on the physicochemical property of interest, thereby enabling an efficient prediction of key properties of anti-Alzheimer’s herbal compounds. Consequently, the Roman energy descriptors provide a rational and robust basis for QSPR modeling.

## 9 Validation of QSPR models

This section presents the internal and external validation of the developed QSPR models to assess their reliability and predictive accuracy.

### 9.1 Internal validation using Y-randomnization

**FIGURE 5:**
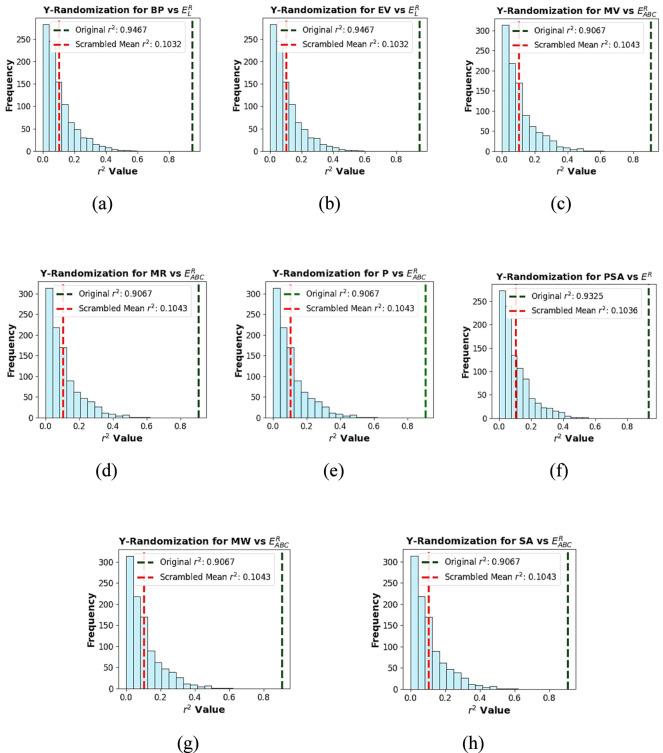
Y-randomization $r^{2}$ distribution plot showing the difference between the original $r^{2}$ and scrambled mean $r^{2}$ for **(a)** $E_{L}^{R}$ with BP , **(b)** $E_{L}^{R}$ with EV , **(c)** $E_{ABC}^{R}$ with MV , **(d)** $E_{ABC}^{R}$ with MR , **(e)** $E_{ABC}^{R}$ with P , **(f)** $E^{R}$ with PSA , **(g)** $E_{ABC}^{R}$ with MW and **(h)** $E_{ABC}^{R}$ with SA .

To ensure that the observed correlations are not due to chance, a Y-randomization test is performed. In this procedure, the dependent variable (Y, experimental value of the physicochemical property) is randomly permuted while keeping the independent variables (X, Roman energies) are kept fixed. For each randomized dataset, a quadratic regression model is constructed under the same conditions as the original model and the corresponding scrambled $r^{2}$ values are recorded. This process is repeated 1000 times, generating a distribution of scrambled $r^{2}$ scores that is then compared with the original regression performance. Figure 5 illustrates the difference between the original $r^{2}$ and scrambled $r^{2}$ scores over 1000 iterations.

The results revealed that the original $r^{2}$ values are consistently and substantially higher than the scrambled mean $r^{2}$ , with an average difference of about 0.8. Furthermore, the randomized models satisfy the accepted threshold scrambled $\left(r^{2}<0.2\right)$ , confirming that the predictive performance is not due to chance. Overall, the Y-randomization test demonstrates that the quadratic regression models are statistically valid, robust and capture genuine relationships between Roman energies and physicochemical properties.

### 9.2 Internal validation using leave-one-out cross validation (LOOCV)

The Leave-One-Out Cross-Validation (LOOCV) method is employed to evaluate the internal predictive performance of the quadratic regression models. In this approach, each sample is left out once, the model is trained on the remaining n−1 samples and the excluded point is predicted.

**FIGURE 6:**
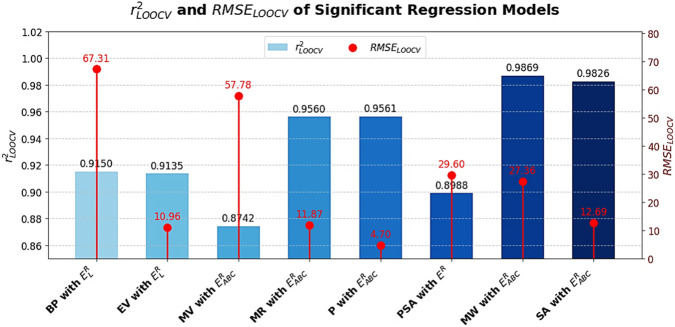
$r_{LOOCV}^{2}$ and $RMSE_{LOOCV}$ values of significant regression models.

The predictive squared correlation coefficient $\left(Q_{LOOCV}^{2}\right)$ and root mean squared error $\left(RMSE_{LOOCV}\right)$ are calculated as: $r_{LOOCV}^{2}=1-\frac{\sum_{i=1}^{n}{\left(y_{i}-y_{i,-i}^{pred}\right)}^{2}}{\sum_{i=1}^{n}{\left(y_{i}-\bar{y}\right)}^{2}}$ and $RMSE_{LOOCV}=\sqrt{\frac{1}{n}\sum_{i=1}^{n}\left(y_{i}-y_{i,-i}^{pred}\right)}$ , where $y_{i}$ is the actual observed value for sample i , $y_{i,-i}^{pred}$ is the predicted value for sample i from a model trained without sample i and $\bar{y}$ is the mean of all observed values. The LOOCV analysis confirms the strong internal predictive ability of the developed models with $r_{LOOCV}^{2}$ values ranging from 0.8742 to 0.9869 and consistently low $RMSE_{LOOCV}$ values, as shown in Figure 6. These results highlight the accuracy, stability and generalizability of the proposed Roman domination-based energies for QSPR modeling.

### 9.3 External validation using relative error (RE)

**FIGURE 7:**
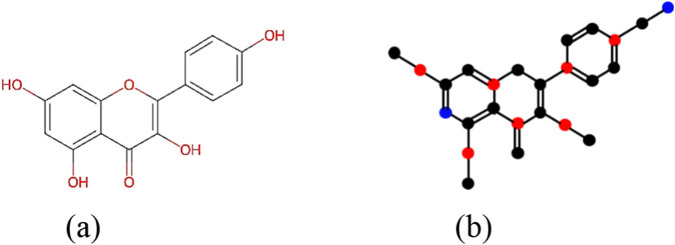
**(a)** Chemical structure and **(b)** Roman domination of the isomorphic molecular graph of Kaempferol.

The developed predictive models are externally validated by using the phytochemical compound Kaempferol as the test compound. The chemical structure and the Roman domination of the isomorphic molecular graph of Kaempferol are shown in Figure 7.

**FIGURE 8:**
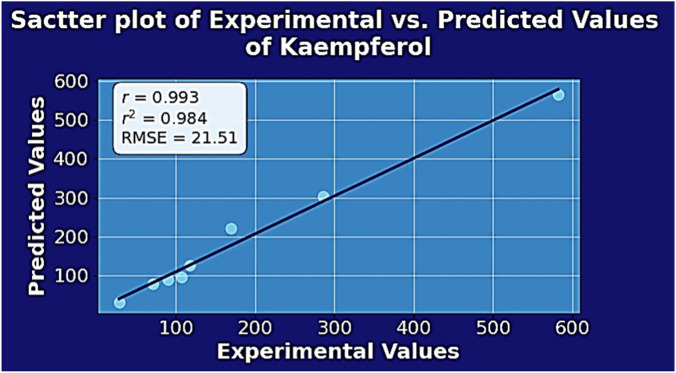
Scatter plot between the experimental vs. predicted values of physicochemical properties of Kaempferol.

Using the most prominent Roman energies ( $E^{R}=56.6967,E_{L}^{R}=85.2000$ and $E_{ABC}^{R}=28.8655$ ), the predicted physicochemical property values are calculated and compared with the experimental (observed) values. For external validation using Relative Error ( RE ), the interpretation depends on the magnitude of the error, indicating how far the predicted values deviate from the observed values. Mathematically, it is expressed as: $RE=\frac{\left|y^{pred}-y^{obs}\right|}{y^{obs}}\times 100\%$ , where $y^{pred}$ is the predicted value and $y^{obs}$ ​ is the observed value. Predictive accuracy is evaluated using relative error analysis, with the following criteria: <5% (excellent), 5%–10% (very good), 10%–15% (good), 15%–20% (moderate), 20%–35% (acceptable), and >35% (poor). Based on this classification, the boiling point (BP, 2.85%) and enthalpy of vaporization (EV, 0.14%) exhibits excellent predictive accuracy. The molar volume (MV, 31.44%) fell within the acceptable range. The molar refractivity (MR, 12.24%) and polarizability (P, 12.26%) are classified as good predictions. The polar surface area (PSA, 10.41%), molecular weight (MW, 5.96%) and surface area (SA, 7.81%) demonstrated very good predictive performance. Figure 8 illustrates the scatter plot between the experimental and predicted values of physicochemical properties of Kaempferol. Overall, the validation confirms the robustness of the model with strong correlation $\left(r=0.993\right)$ between predicted and experimental values, highlighting its reliability for QSPR analysis.

## 10 Conclusion

In this work, a novel graph molecular modeling based on Roman domination was proposed for the quantitative structure-property relationship (QSPR) analysis of anti-Alzheimer’s phytochemicals. Unlike classical graph energies such as adjacency and Laplacian energies, which capture only connectivity patterns, the newly introduced Roman domination-based energies encode hierarchical dominance among atoms, thereby offering a deeper structural perspective of molecular graphs.

The QSPR models were developed using these Roman energies through linear, quadratic and cubic regression equations. The results demonstrated superior performance compared to classical approaches, with quadratic regression showing the strongest correlations and the lowest standard error. Internal validation through Y-randomization and LOOCV confirmed the stability of the models, while external validation on Kaempferol ( r=0.993 ) further supported their predictive reliability. These findings underscore the robustness and generalizability of Roman domination-based energies.

Overall, this research establishes Roman energies as powerful molecular descriptors that significantly enhance the accuracy of QSPR modeling. Beyond Alzheimer’s related phytochemicals, the methodology has potential for broader applications in drug discovery, materials informatics and computational chemistry, paving the way for the integration of graph-theoretic principles with predictive modeling in cheminformatics.

## Data Availability

The chemical structures of the herbal compounds used in this research work are taken from the website https://pubchem.ncbi.nlm.nih.gov/ and physicochemical properties of the herbal compounds are taken from the website www.chemspider.com. The statistical analysis of the work is carried out by the SPSS software and python programming. The python code to calculate the classical and Roman energies are provided in the Supplementary Material.
