# Supplementary material for: Novel Roman domination-based graph energies for QSPR analysis of neuroprotective herbal compounds in Alzheimer’s disease treatment
Source: Front Chem. 2026 Mar 13;14:1731656. doi: 10.3389/fchem.2026.1731656 (PMC13022923; doi:10.3389/fchem.2026.1731656)
Supplement: Supplementary file 1 [file DataSheet1.pdf]

Supplementary Material

1 Supplementary Figures

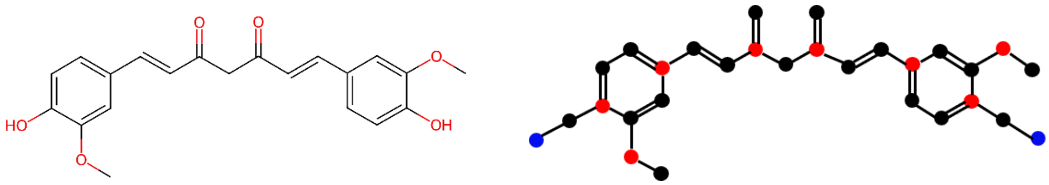

(a) (b)

Supplementary Figure 1. (a) Chemical structure and (b) Roman domination of the isomorphic molecular graph of Curcumin.

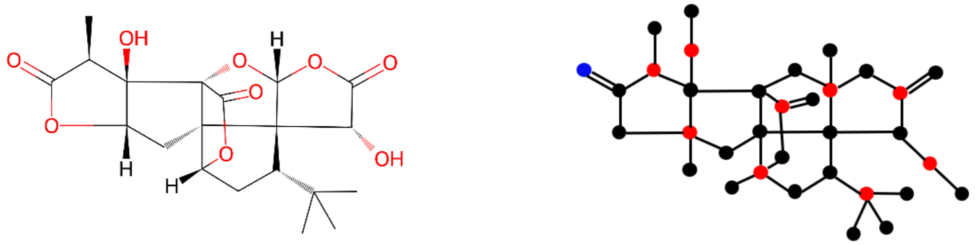

(a) (b)

Supplementary Figure 2. (a) Chemical structure and (b) Roman domination of the isomorphic molecular graph of Ginkgolide.

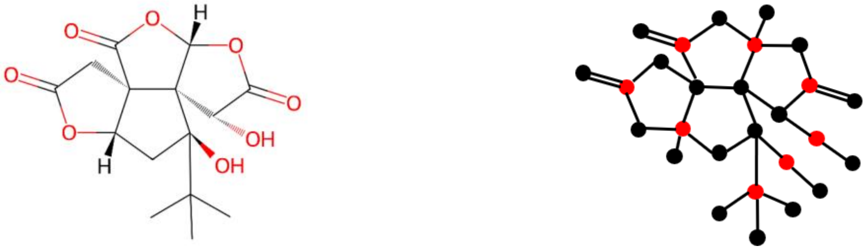

(a) (b)

Supplementary Figure 3. (a) Chemical structure and (b) Roman domination of the isomorphic molecular graph of Bilobalide.

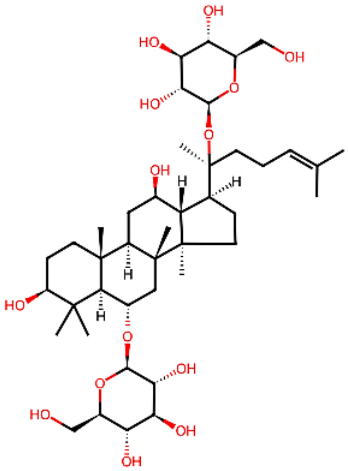

(a)

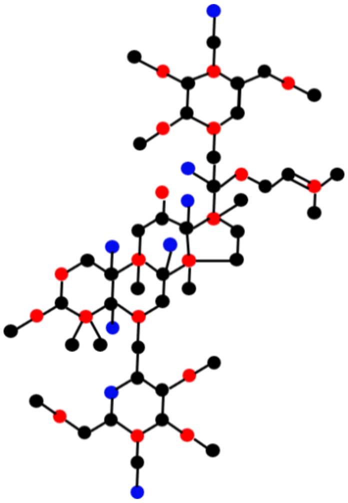

(b)

Supplementary Figure 4. (a) Chemical structure and (b) Roman domination of the isomorphic molecular graph of Ginsenoside Rg1.

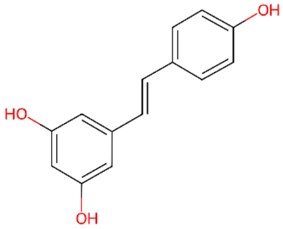

(a)

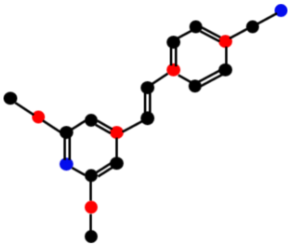

(b)

Supplementary Figure 5. (a) Chemical structure and (b) Roman domination of the isomorphic molecular graph of Resveratrol.

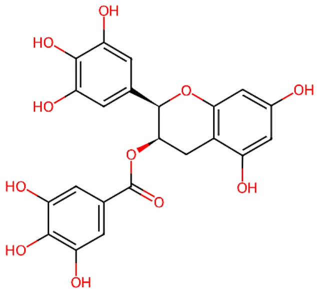

(a)

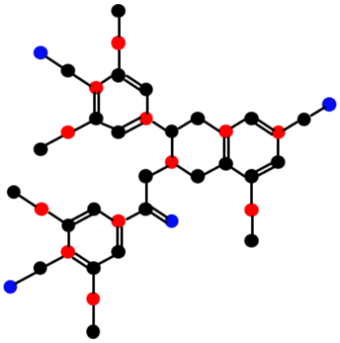

(b)

Supplementary Figure 6. (a) Chemical structure and (b) Roman domination of the isomorphic molecular graph of Epigallocatechin-3-gallate.

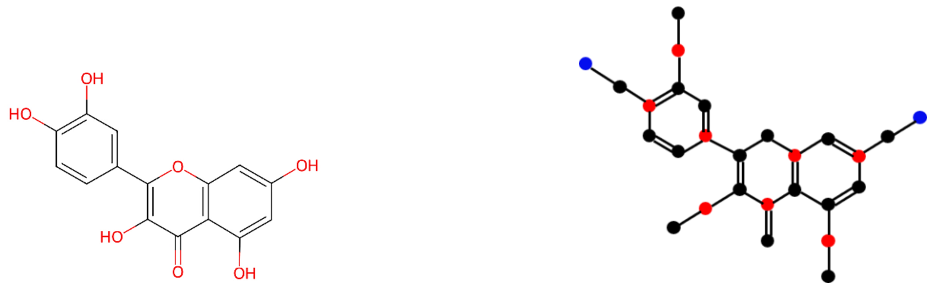

(a) (b)

Supplementary Figure 7. (a) Chemical structure and (b) Roman domination of the isomorphic molecular graph of Quercetin.

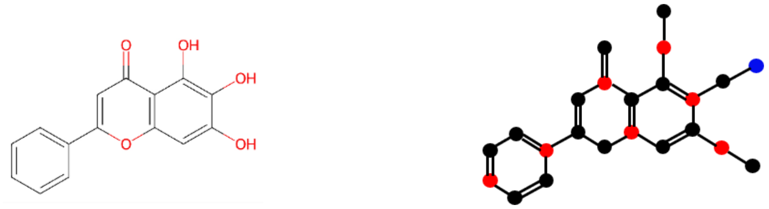

(a) (b)

Supplementary Figure 8. (a) Chemical structure and (b) Roman domination of the isomorphic molecular graph of Baicalein.

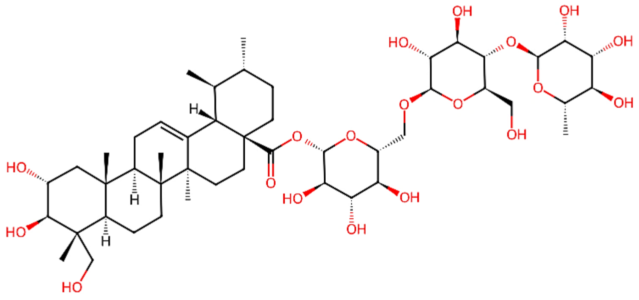

(a)

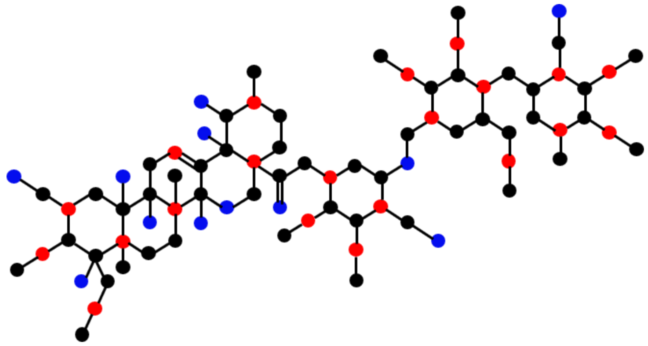

(b)

Supplementary Figure 9. (a) Chemical structure and (b) Roman domination of the isomorphic molecular graph of Asiaticoside.

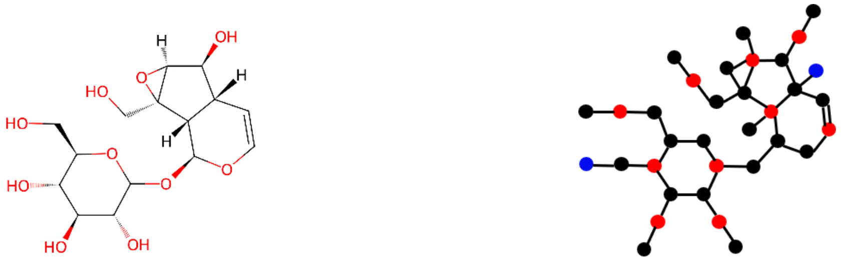

(a) (b)

Supplementary Figure 10. (a) Chemical structure and (b) Roman domination of the isomorphic molecular graph of Catalpol.

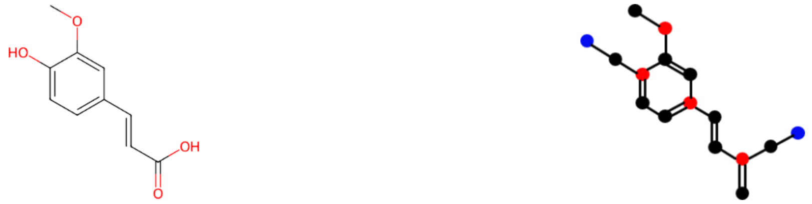

(a) (b)

Supplementary Figure 11. (a) Chemical structure and (b) Roman domination of the isomorphic molecular graph of Ferulic acid.

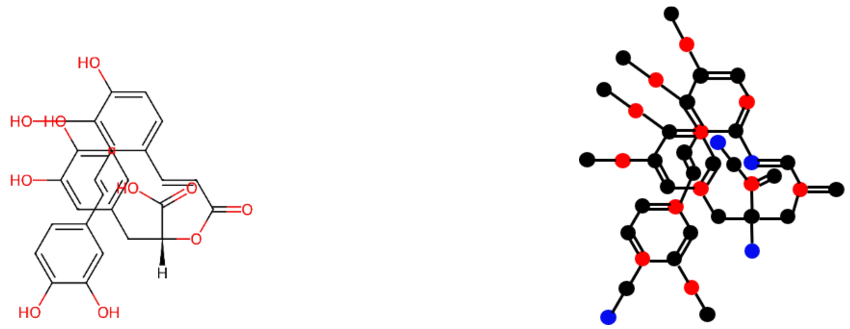

(a) (b)

Supplementary Figure 12. (a) Chemical structure and (b) Roman domination of the isomorphic molecular graph of Salvianolic acid.

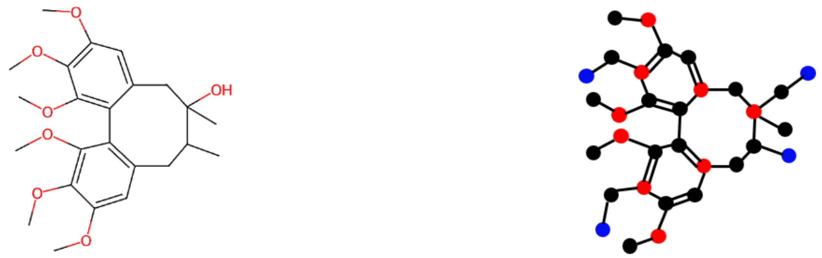

(a) (b)

Supplementary Figure 13. (a) Chemical structure and (b) Roman domination of the isomorphic molecular graph of Schisandrin.

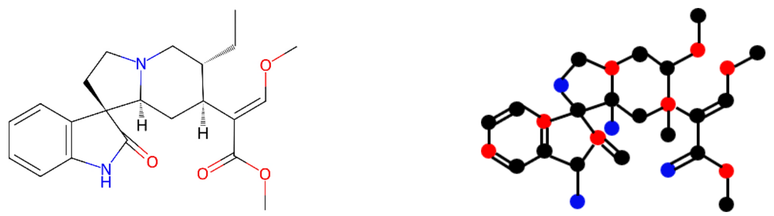

(a) (b)

Supplementary Figure 14. (a) Chemical structure and (b) Roman domination of the isomorphic molecular graph of Rhynchophylline.

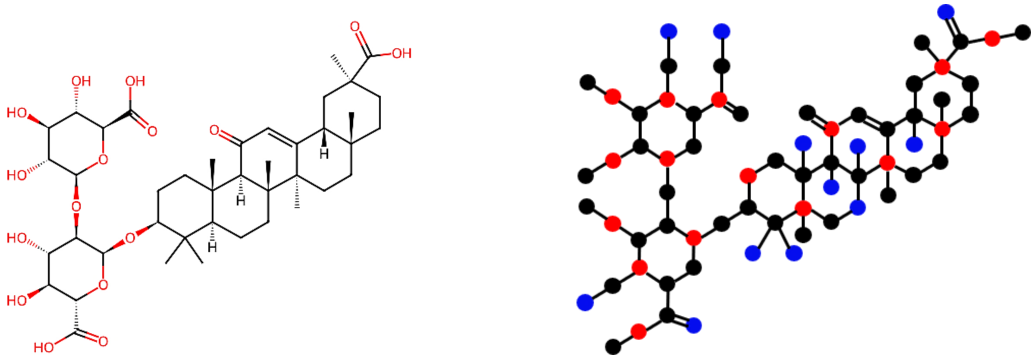

(a) (b)

Supplementary Figure 15. (a) Chemical structure and (b) Roman domination of the isomorphic molecular graph of Glycyrrhizin.

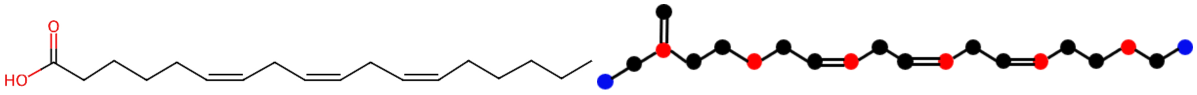

(a) (b)

Supplementary Figure 16. (a) Chemical structure and (b) Roman domination of the isomorphic molecular graph of γ-linolenic acid.

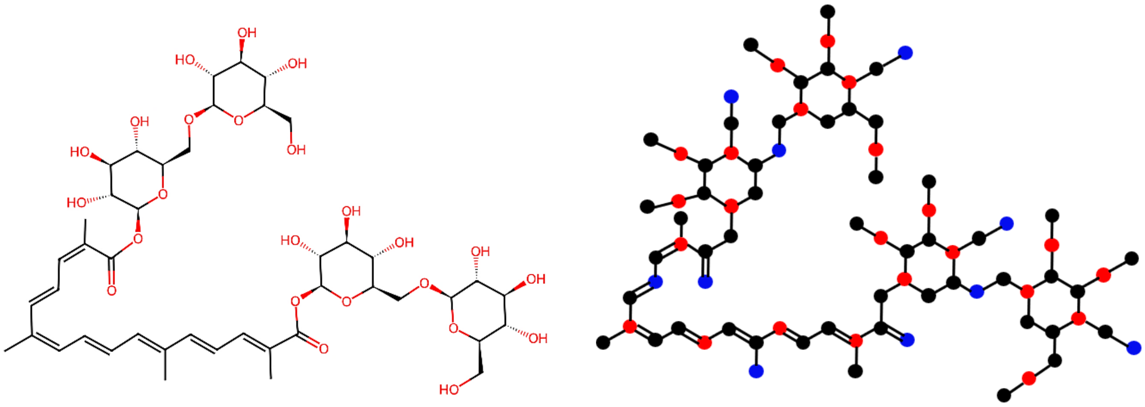

(a) (b)

Supplementary Figure 17. (a) Chemical structure and (b) Roman domination of the isomorphic molecular graph of Crocin.

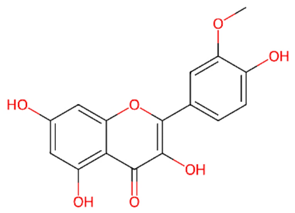

(a)

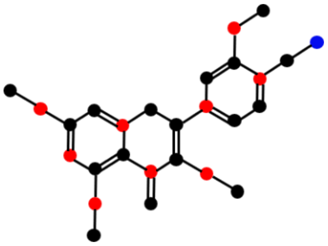

(b)

Supplementary Figure 18. (a) Chemical structure and (b) Roman domination of the isomorphic molecular graph of Isorhamnetin.

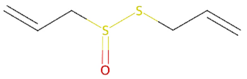

(a)

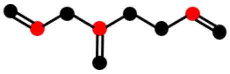

(b)

Supplementary Figure 19. (a) Chemical structure and (b) Roman domination of the isomorphic molecular graph of Allicin.

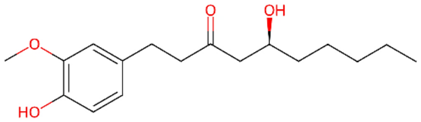

(a)

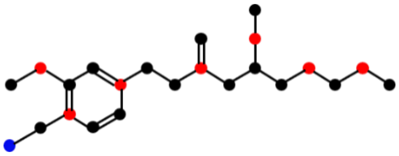

(b)

Supplementary Figure 20. (a) Chemical structure and (b) Roman domination of the isomorphic molecular graph of Gingerol.

2 Python code to find the classical and Roman matrices with their corresponding energies of the compound Huperzine A

# Note that the appropriate input values of number of atoms, bond list, RDF values should be entered correctly when computing graph energies for each compound.

```
import numpy as np

from scipy.linalg import eigvals


# Enter the number of atoms (n)

n = 22
```

# Enter the bond list

```
bonds = [
    (1,2), (1,2),  # Two bonds between v1 and v2
    (2,3),
    (3,4),
    (4,5),
    (5,6),
    (6,7),
    (7,8), (7,8),
    (8,9),
    (9,10),
    (10,11),
    (11,12),
    (12,13), (12,13),
    (13,2),
    (3,14),
    (15,16),
    (16,17), (16,17),
    (17,6),
    (17,10),
    (6,18),
    (8,19),
    (10,20),
    (20,21),
```

```
    (20,22),
    (4,11), (4,11)
]
```

# Defining count total bonds

```
def count_total_bonds(bonds):
    return len(bonds)
m = count_total_bonds(bonds)
```

# Enter Roman domination function (RDF) values of all the vertices

```
roman_domination = {1:0, 2:2, 3:0, 4:0, 5:0, 6:2, 7:0, 8:2, 9:0, 10:0, 11:2, 12:0, 13:0, 14:1, 15:0,
16:2, 17:0, 18:0, 19:0, 20:2, 21:0, 22:0}
```

# Classical matrices and energies

# 1. Adjacency matrix and Graph energy

```
def adjacency_matrix(n, bonds):
    AM = np.zeros((n, n), dtype=int)
    for u, v in bonds:
        AM[u-1, v-1] += 1
        AM[v-1, u-1] += 1  # Since the graph is undirected
    return AM
AM = adjacency_matrix(n, bonds)
print("Adjacency Matrix A(G):")
print(AM)
def graph_energy_and_characteristic_polynomialAM(AM):
```

```
    B = np.array(AM)
    if B.shape[0] != B.shape[1]:
        raise ValueError("The adjacency matrix must be square.")
    eigenvaluesAM = np.linalg.eigvals(B)
    print("Eigenvalues:", eigenvaluesAM)
    energyAM = np.sum(np.abs(eigenvaluesAM))
    char_polyAM = np.poly(B)
    print("Characteristic Polynomial Coefficients of AM:")
    print(char_polyAM)
    return energyAM, char_polyAM
energyAM, char_polyAM = graph_energy_and_characteristic_polynomialAM(AM)
print("Graph Energy from AM:", energyAM)
```

# 2. Laplacian matrix and Laplacian energy

```
def degree_matrix(adj):
    deg = np.sum(adj, axis=1)
    return np.diag(deg), deg
def laplacian_matrix(adj):
    D, _ = degree_matrix(adj)
    return D - adj
def laplacian_energy(adj):
    L = laplacian_matrix(adj)
    print("Laplacian Matrix:\n", laplacian_matrix(AM))
    eigenvalues = eigvals(L)
    energy = np.abs(np.sum(eigenvalues) - ((2 * m) / n))
```

```
    print("Eigenvalues of Laplacian Matrix:", np.real_if_close(eigenvalues))
    char_poly_L = np.poly(L)
    print("Char poly of Laplacian matrix:")
    print(char_poly_L)
    return np.real_if_close(energy)
print("Laplacian Matrix:\n", laplacian_matrix(AM))
print("Laplacian Energy:", laplacian_energy(AM))
```

# 3. Randic matrix and Randic energy

```
def randic_matrix(adj):
    n = adj.shape[0]
    _, deg = degree_matrix(adj)
    Ra = np.zeros((n, n))
    for i in range(n):
        for j in range(n):
            if adj[i][j] != 0:
                Ra[i][j] = 1 / np.sqrt(deg[i]*deg[j])
    return Ra
def randic_energy(adj):
    Ra = randic_matrix(adj)
    eigenvalues = eigvals(Ra)
    print("Eigenvalues of Randic Matrix:", np.real_if_close(eigenvalues))
    char_poly_Ra = np.poly(Ra)
    print("Char poly of Randix matrix:")
    print(char_poly_Ra)
```

```
    return np.sum(np.abs(np.real_if_close(eigenvalues)))
print("\nRandic Matrix:\n", randic_matrix(AM))
print("Randic Energy:", randic_energy(AM))
```

# 4. Harmonic Matrix and Harmonic energy

```
def degree_vector(adj):
    return np.sum(adj, axis=1)
def matrix_energy(matrix):
    eigenvalues = eigvals(matrix)
    return np.sum(np.abs(np.real_if_close(eigenvalues)))
def harmonic_matrix(adj):
    n = adj.shape[0]
    deg = degree_vector(adj)
    H = np.zeros((n, n))
    for i in range(n):
        for j in range(n):
            if adj[i][j] != 0 and deg[i] + deg[j] != 0:
                H[i][j] = 2 / (deg[i] + deg[j])
    return H
def harmonic_energy(adj):
    H = harmonic_matrix(adj)
    eigenvalues = eigvals(H)
    print("Eigenvalues of Harmonic Matrix:", np.real_if_close(eigenvalues))
    char_poly_H = np.poly(H)
```

```
    print("Char poly of Harmonic matrix:")
    print(char_poly_H)
    return np.sum(np.abs(np.real_if_close(eigenvalues)))
print("\n Harmonic Matrix:\n", harmonic_matrix(AM))
print(" Harmonic Energy:", harmonic_energy(AM))
```

# 5. ABC Matrix and ABC energy

```
def abc_matrix(adj):
    n = adj.shape[0]
    deg = degree_vector(adj)
    ABC = np.zeros((n, n))
    for i in range(n):
        for j in range(n):
            if adj[i][j] != 0 and deg[i]*deg[j] != 0:
                numerator = deg[i] + deg[j] - 2
                denominator = deg[i] * deg[j]
                if denominator != 0 and numerator >= 0:
                    ABC[i][j] = np.sqrt(numerator / denominator)
    return ABC
def abc_energy(adj):
    ABC = abc_matrix(adj)
    eigenvalues = eigvals(ABC)
    print("Eigenvalues of ABC Matrix:", np.real_if_close(eigenvalues))
    char_poly_ABC = np.poly(ABC)
    print("Char poly of ABC matrix:")
```

```
    print(char_poly_ABC)
    return np.sum(np.abs(np.real_if_close(eigenvalues)))
print("\n ABC Matrix:\n", abc_matrix(AM))
print("ABC Energy:", abc_energy(AM))
```

# 6. GA Matrix and GA energy

```
def ga_matrix(adj):
    n = adj.shape[0]
    deg = degree_vector(adj)
    GA = np.zeros((n, n))
    for i in range(n):
        for j in range(n):
            if adj[i][j] != 0 and deg[i] + deg[j] != 0:
                GA[i][j] = (2 * np.sqrt(deg[i] * deg[j])) / (deg[i] + deg[j])
    return GA
def ga_energy(adj):
    GA = ga_matrix(adj)
    eigenvalues = eigvals(GA)
    print("Eigenvalues of GA Matrix:", np.real_if_close(eigenvalues))
    char_poly_GA = np.poly(GA)
    print("Char poly of GA matrix:")
    print(char_poly_GA)
    return np.sum(np.abs(np.real_if_close(eigenvalues)))
print("\n GA Matrix:\n", ga_matrix(AM))
print(" GA Energy:", ga_energy(AM))
```

# Roman domination-based matrices and energies

# 1. Roman domination-based adjacency matrix (RA matrix) and Roman energy

```
def roman_adjacency_matrix(n, bonds, roman_domination):
    E_matrix = np.zeros((n, n), dtype=int)
    for u, v in bonds:
        E_matrix[u-1, v-1] += 1
        E_matrix[v-1, u-1] += 1  # Since the graph is undirected
    RA = np.zeros((n, n), dtype=int)
    for i in range(n):
        for j in range(n):
            if i == j:
                RA[i, j] = roman_domination[i+1]
            else:
                if E_matrix[i, j] > 0:
                    if roman_domination[j+1] == 2:
                        RA[i, j] = E_matrix[i, j] + 1
                    elif roman_domination[j+1] in [0,1]:
                        RA[i, j] = E_matrix[i, j]
                else:
                    RA[i, j] = 0
    return RA
RA = roman_adjacency_matrix(n, bonds, roman_domination)
print("Roman Adjacency Matrix RA(G):")
print(RA)
```

```
def graph_energy_and_characteristic_polynomial(RA):
    A = np.array(RA)
    if A.shape[0] != A.shape[1]:
        raise ValueError("The adjacency matrix must be square.")
    eigenvalues = np.linalg.eigvals(A)
    print("Eigenvalues:", eigenvalues)
    energy = np.sum(np.abs(eigenvalues))
    char_poly = np.poly(A)
    print("Characteristic Polynomial Coefficients:")
    print(char_poly)
    return energy, char_poly
energy, char_poly = graph_energy_and_characteristic_polynomial(RA)
print("Roman Energy:", energy)
```

# 2. Roman Laplacian matrix and Roman Laplacian energy

```
def degree_vector(adj):
    return np.sum(adj, axis=1)
def roman_laplacian_matrix(adj):
    D, _ = degree_matrix(adj)
    return D - adj
def roman_laplacian_energy(RA):
    RL = roman_laplacian_matrix(RA)
    print("\nRoman Laplacian Matrix:\n", roman_laplacian_matrix(RA))
    eigenvalues = eigvals(RL)
    energy = np.abs(np.sum(eigenvalues) - ((2 * m) / n))
```

```
    print("Eigenvalues of Roman Laplacian Matrix:", np.real_if_close(eigenvalues))
    char_poly_RL = np.poly(RL)
    print("Char poly of Roman laplacian matrix:")
    print(char_poly_RL)
    return np.real_if_close(energy)
roman_lap_energy = roman_laplacian_energy(RA)
print("Roman Laplacian Energy:", roman_lap_energy)
```

# 3. Roman Randic matrix and Roman Randic energy

```
def get_rdf_list(rdf_dict, n):
    return [rdf_dict[i+1] for i in range(n)]
def roman_randic_matrix(adj, rdf_dict):
    n = adj.shape[0]
    deg = degree_vector(adj)
    rdf = get_rdf_list(rdf_dict, n)
    Ra_R = np.zeros((n, n))
    for i in range(n):
        for j in range(n):
            if i == j:
                Ra_R[i][j] = rdf[i]
            elif adj[i][j] != 0:
                Ra_R[i][j] = 1 / np.sqrt(deg[i] * deg[j])
    return Ra_R
def roman_randic_energy(adj, rdf_dict):
    Ra_R = roman_randic_matrix(adj, rdf_dict)
```

```
    eigenvalues = eigvals(Ra_R)
    print("Eigenvalues of Roman Randic Matrix:", np.real_if_close(eigenvalues))
    char_poly_Ra_R = np.poly(Ra_R)
    print("Char poly of Roman Randic matrix:")
    print(char_poly_Ra_R)
    return np.sum(np.abs(np.real_if_close(eigenvalues)))
print("\nRoman Randic Matrix:\n", roman_randic_matrix(AM, roman_domination))
print("Roman Randic Energy:", roman_randic_energy(AM, roman_domination))
```

# 4. Roman Harmonic Matrix and Roman Harmonic energy

```
def roman_harmonic_matrix(adj, rdf_dict):
    n = adj.shape[0]
    deg = degree_vector(adj)
    rdf = get_rdf_list(rdf_dict, n)
    R_H = np.zeros((n, n))
    for i in range(n):
        for j in range(n):
            if i == j:
                R_H[i][j] = rdf[i]
            elif adj[i][j] != 0 and deg[i] + deg[j] != 0:
                R_H[i][j] = 2 / (deg[i] + deg[j])
    return R_H
def roman_harmonic_energy(adj, rdf_dict):
    R_H = roman_harmonic_matrix(adj, rdf_dict)
    eigenvalues = eigvals(R_H)
```

```
    print("Eigenvalues of Roman Harmonic Matrix:", np.real_if_close(eigenvalues))
    char_poly_R_H = np.poly(R_H)
    print("Char poly of Roman harmonic matrix:")
    print(char_poly_R_H)
    return np.sum(np.abs(np.real_if_close(eigenvalues)))
print("\nRoman Harmonic Matrix:\n", roman_harmonic_matrix(AM, roman_domination))
print("Roman Harmonic Energy:", roman_harmonic_energy(AM, roman_domination))
```

# 5. Roman ABC Matrix and Roman ABC energy

```
def roman_abc_matrix(adj, rdf_dict):
    n = adj.shape[0]
    deg = degree_vector(adj)
    rdf = get_rdf_list(rdf_dict, n)
    R_ABC = np.zeros((n, n))
    for i in range(n):
        for j in range(n):
            if i == j:
                R_ABC[i][j] = rdf[i]
            elif adj[i][j] != 0 and deg[i]*deg[j] != 0:
                numerator = deg[i] + deg[j] - 2
                denominator = deg[i] * deg[j]
                if denominator != 0 and numerator >= 0:
                    R_ABC[i][j] = np.sqrt(numerator / denominator)
    return R_ABC
def roman_abc_energy(adj, rdf_dict):
```

```
    R_ABC = roman_abc_matrix(adj, rdf_dict)
    eigenvalues = eigvals(R_ABC)
    print("Eigenvalues of Roman ABC Matrix:", np.real_if_close(eigenvalues))
    char_poly_R_ABC = np.poly(R_ABC)
    print("Char poly of Roman ABC matrix:")
    print(char_poly_R_ABC)
    return np.sum(np.abs(np.real_if_close(eigenvalues)))
print("\nRoman ABC Matrix:\n", roman_abc_matrix(AM, roman_domination))
print("Roman ABC Energy:", roman_abc_energy(AM, roman_domination))
```

# 6. Roman GA Matrix and Roman GA energy

```
def roman_ga_matrix(adj, rdf_dict):
    n = adj.shape[0]
    deg = degree_vector(adj)
    rdf = get_rdf_list(rdf_dict, n)
    R_GA = np.zeros((n, n))
    for i in range(n):
        for j in range(n):
            if i == j:
                R_GA[i][j] = rdf[i]
            elif adj[i][j] != 0 and deg[i] + deg[j] != 0:
                R_GA[i][j] = (2 * np.sqrt(deg[i] * deg[j])) / (deg[i] + deg[j])
    return R_GA
def roman_ga_energy(adj, rdf_dict):
    R_GA = roman_ga_matrix(adj, rdf_dict)
```

```
    eigenvalues = eigvals(R_GA)
    print("Eigenvalues of Roman GA Matrix:", np.real_if_close(eigenvalues))
    char_poly_R_GA = np.poly(R_GA)
    print("Char poly of Roman GA matrix:")
    print(char_poly_R_GA)
    return np.sum(np.abs(np.real_if_close(eigenvalues)))
print("\nRoman GA Matrix:\n", roman_ga_matrix(AM, roman_domination))
print("Roman GA Energy:", roman_ga_energy(AM, roman_domination))
```
